# Supplementary figures and images for: Integrated Transcriptome and Metabolome Analysis Reveals the Molecular Mechanism of Rust Resistance in Resistant (Youkang) and Susceptive (Tengjiao) Zanthoxylum armatum Cultivars (part 2 of 2)
Source: Int J Mol Sci. 2023 Sep 29;24(19):14761. doi: 10.3390/ijms241914761 (PMC10573174; doi:10.3390/ijms241914761)

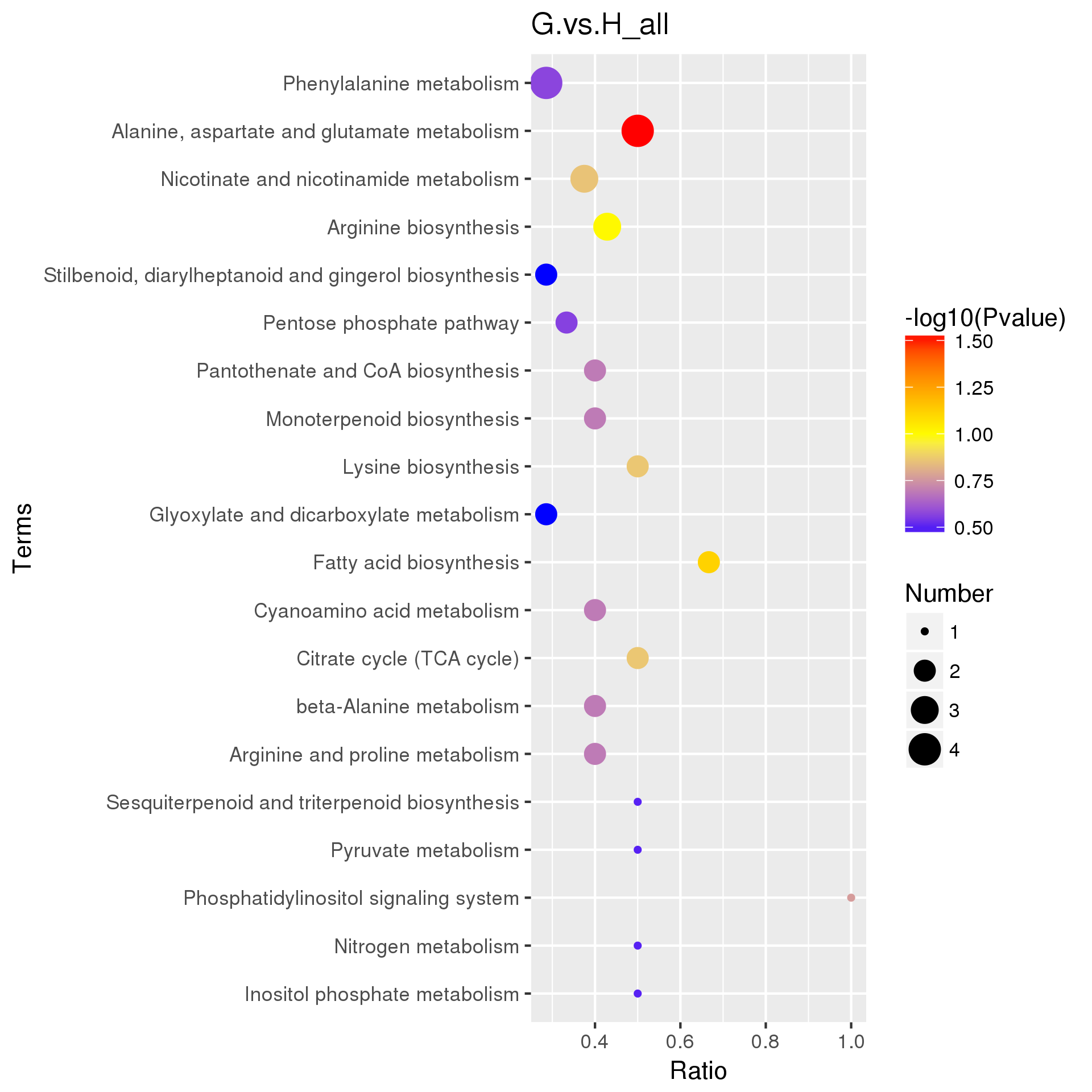

Supplement: Supplementary file 1 [file ijms-24-14761-s001.zip › Figure 8/G.vs.H_all.KEGG_Enrich.scatterplot.png]

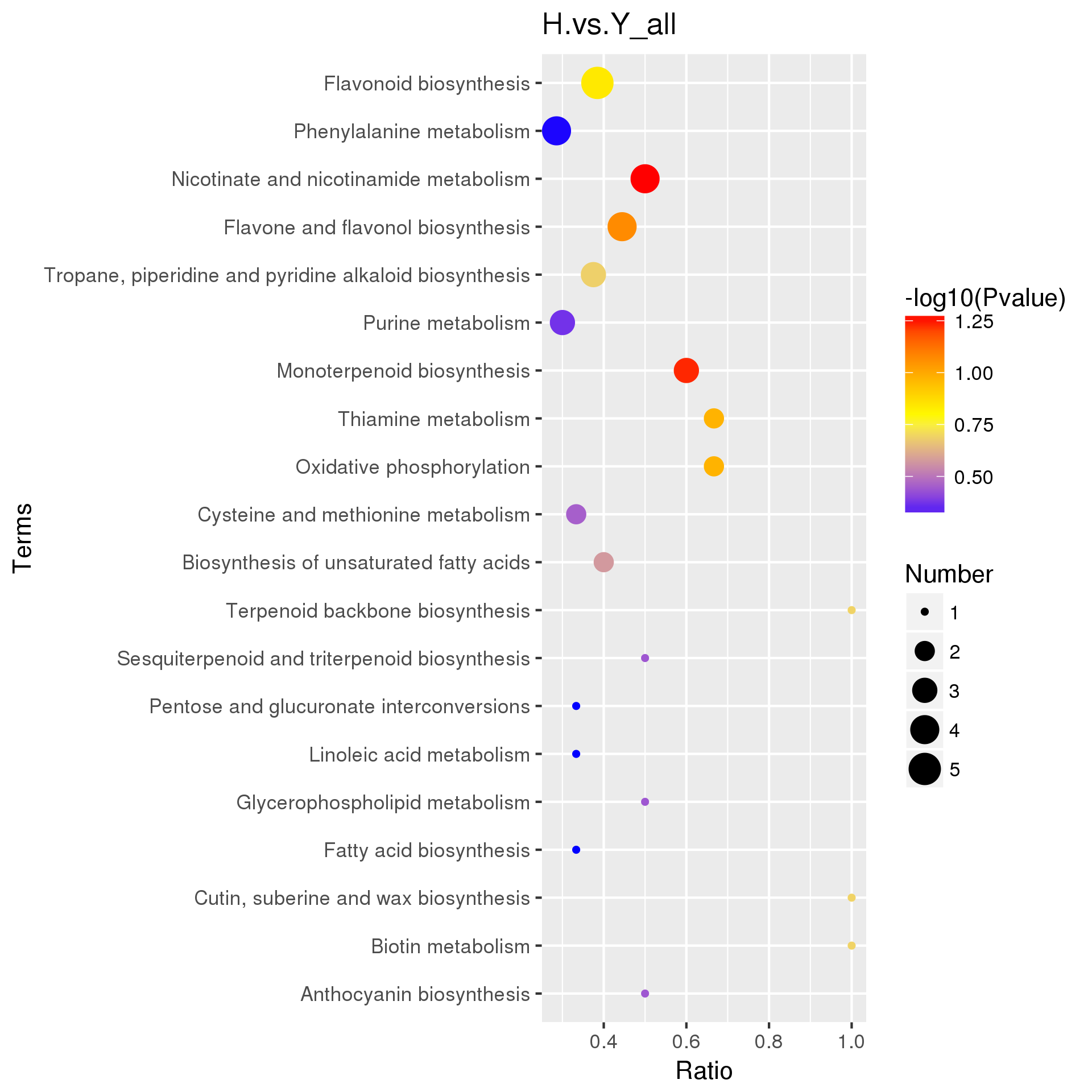

Supplement: Supplementary file 1 [file ijms-24-14761-s001.zip › Figure 8/H.vs.Y_all.KEGG_Enrich.scatterplot.png]

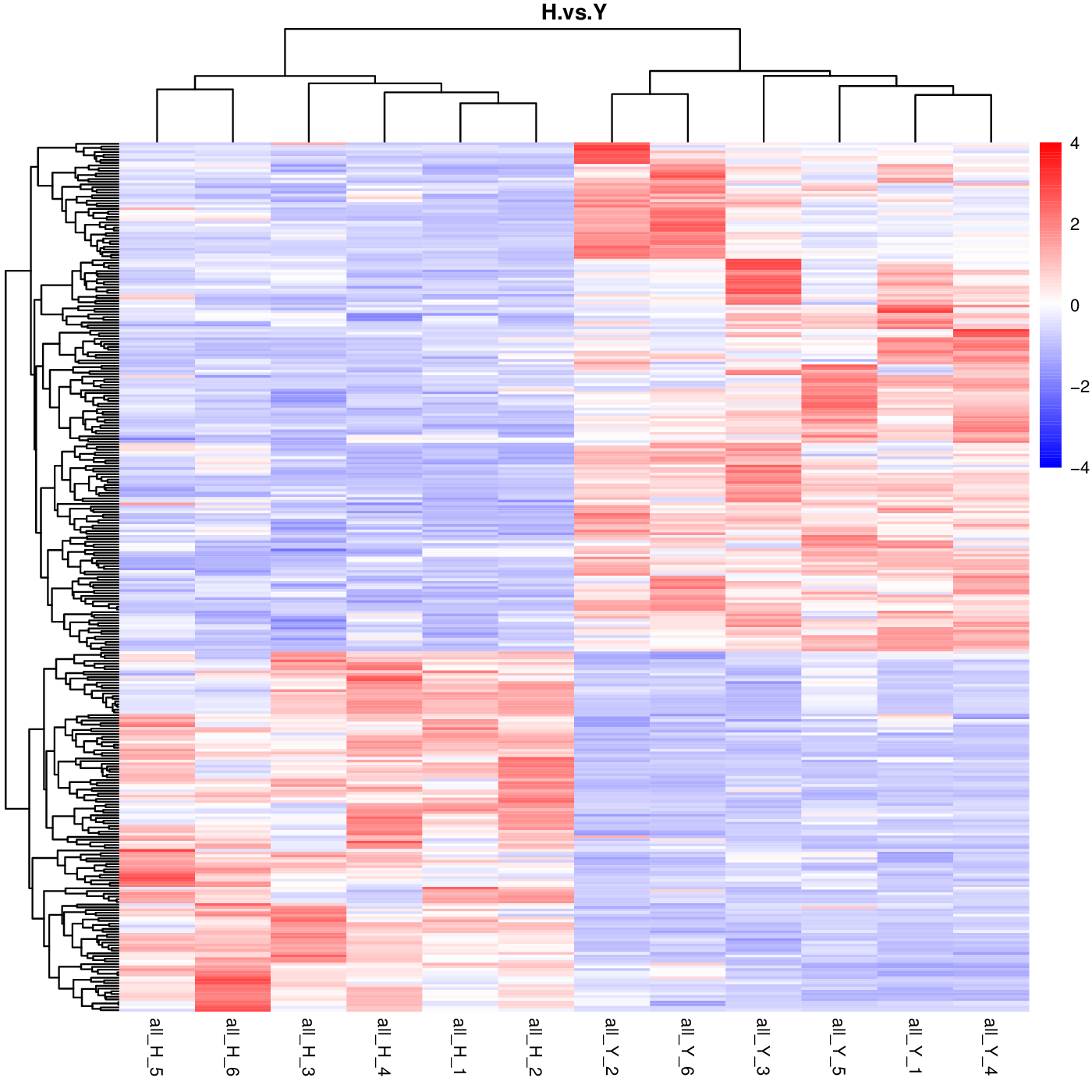

Supplement: Supplementary file 1 [file ijms-24-14761-s001.zip › Figure 9/H.vs.Y_all_cluster_heatmap.png]
