# Supplementary figures and images for: Integrated Transcriptome and Metabolome Analysis Reveals the Molecular Mechanism of Rust Resistance in Resistant (Youkang) and Susceptive (Tengjiao) Zanthoxylum armatum Cultivars (part 1 of 2)
Source: Int J Mol Sci. 2023 Sep 29;24(19):14761. doi: 10.3390/ijms241914761 (PMC10573174; doi:10.3390/ijms241914761)

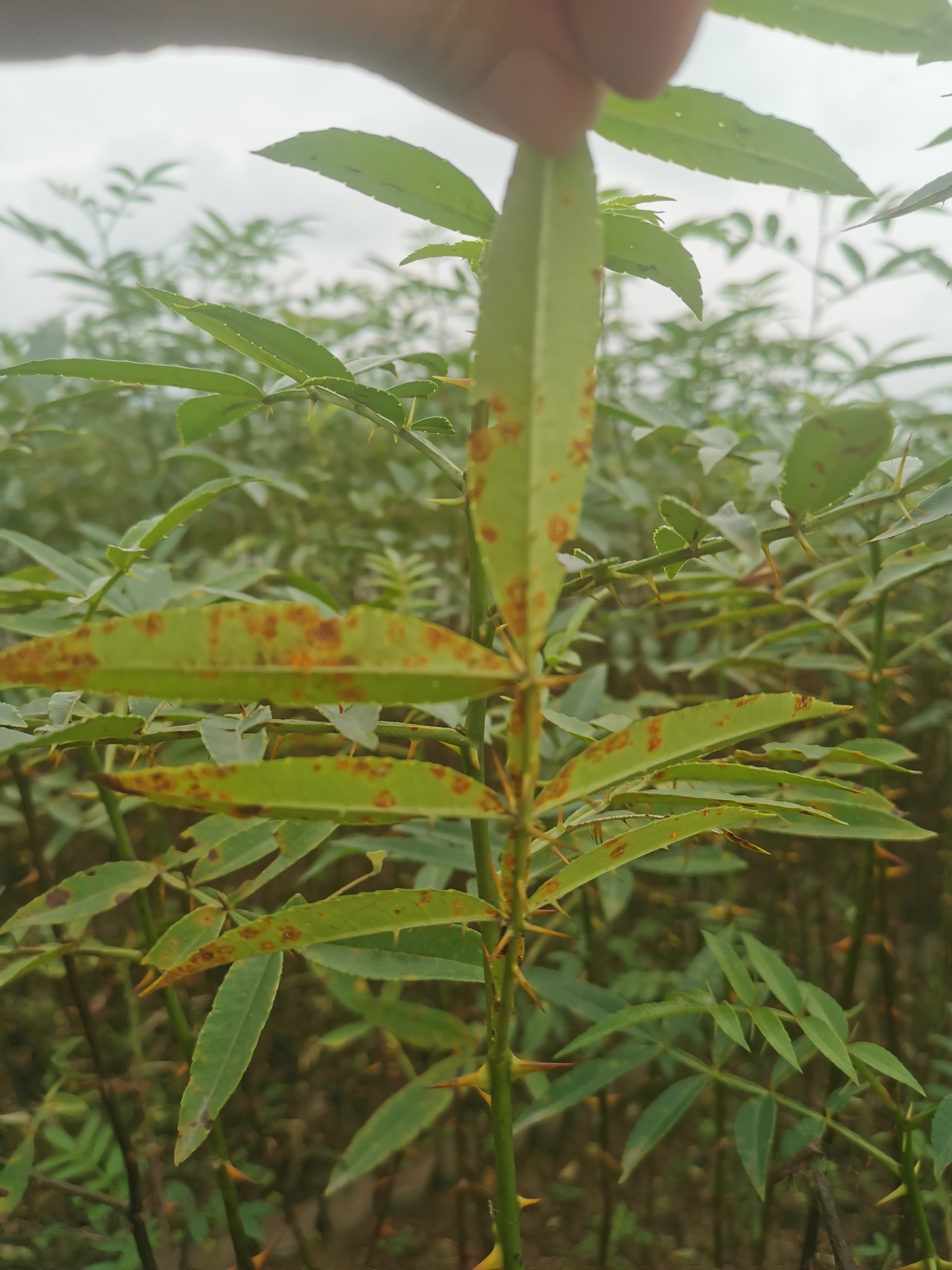

Supplement: Supplementary file 1 [file ijms-24-14761-s001.zip › Figure 1/Tengjiao.jpg]

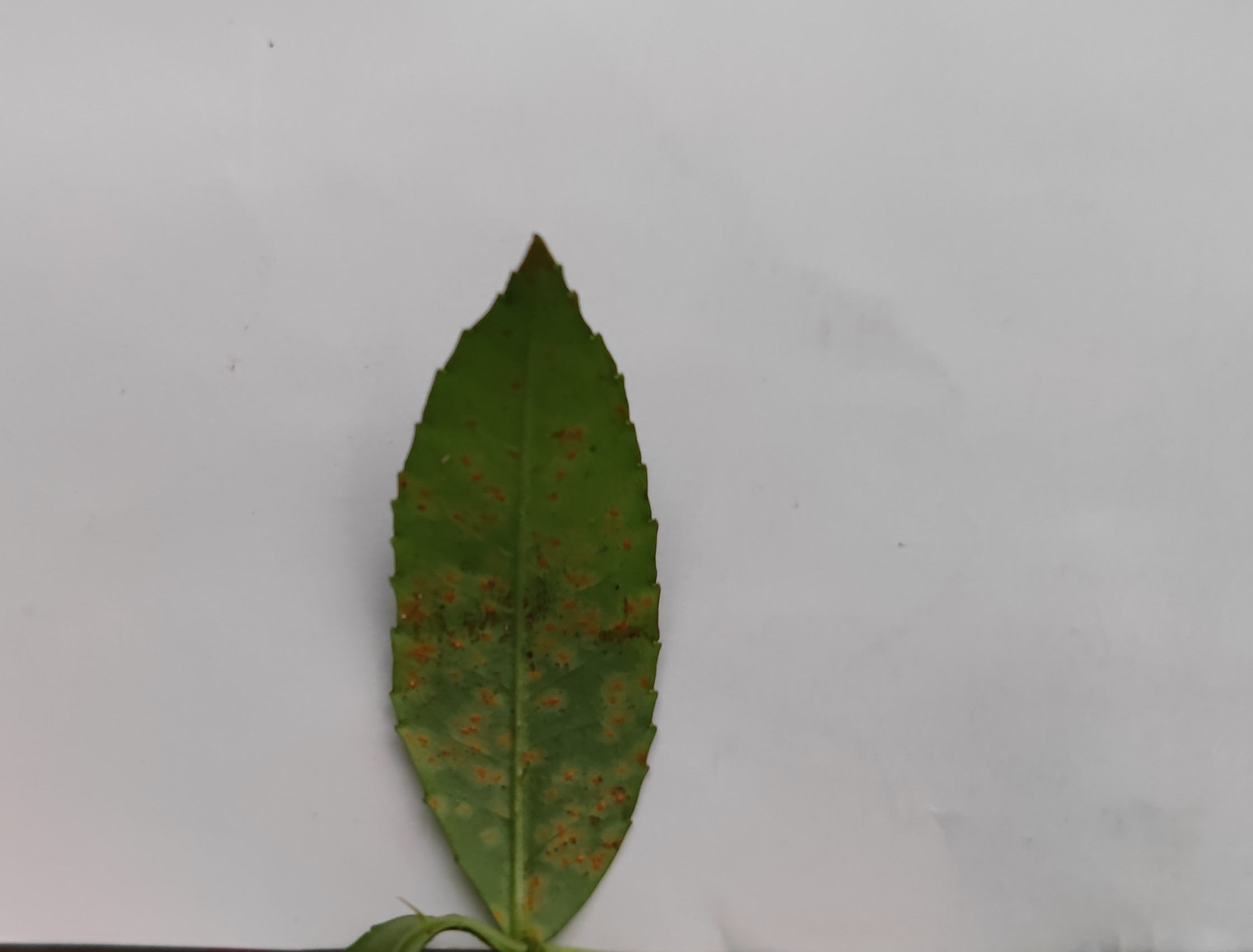

Supplement: Supplementary file 1 [file ijms-24-14761-s001.zip › Figure 1/Tengjiao-inoculated with C. zanthoxyli/1.jpg]

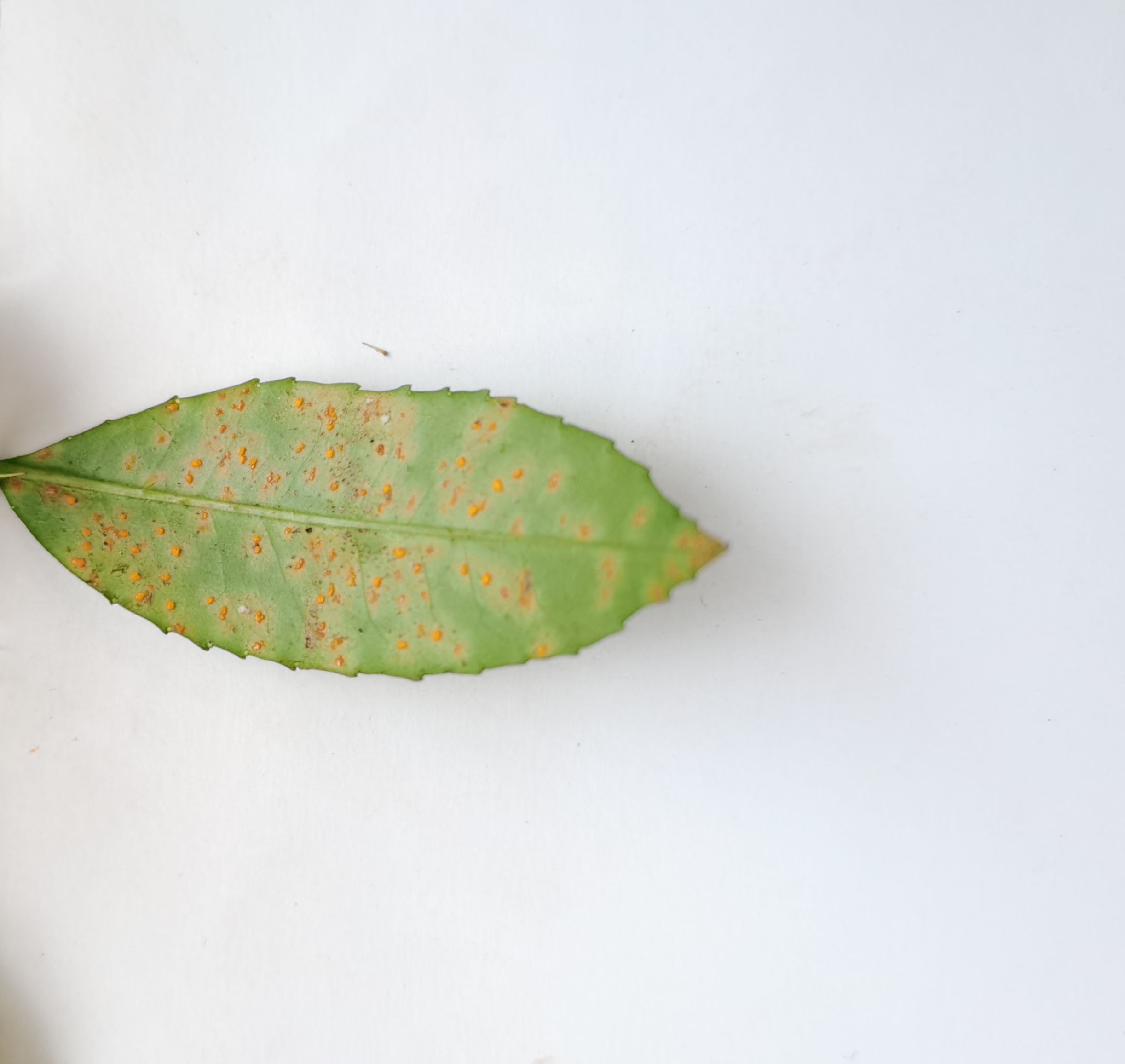

Supplement: Supplementary file 1 [file ijms-24-14761-s001.zip › Figure 1/Tengjiao-inoculated with C. zanthoxyli/335F75E0BEF632857F2A18BF1D527861.jpg]

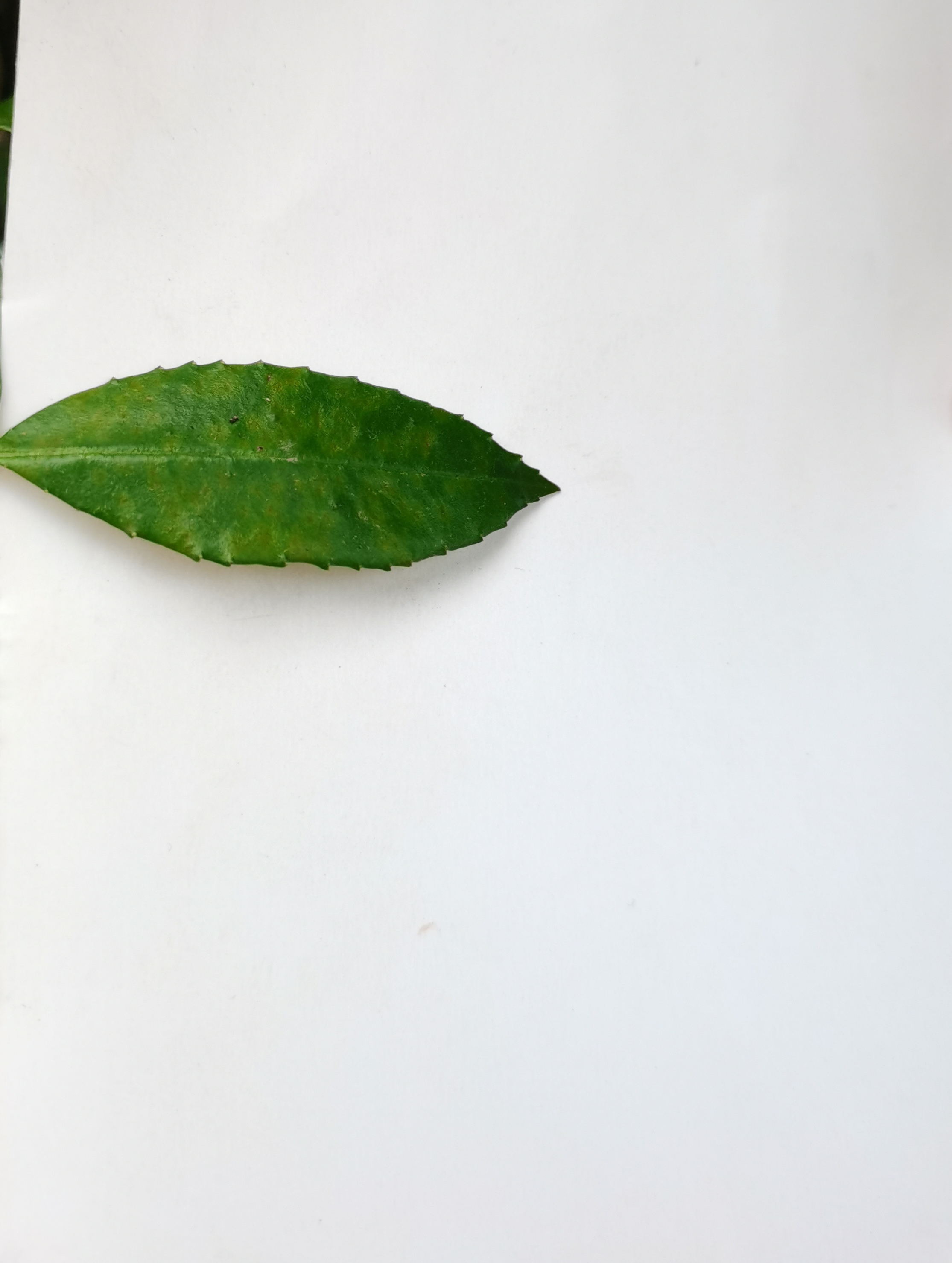

Supplement: Supplementary file 1 [file ijms-24-14761-s001.zip › Figure 1/Tengjiao-inoculated with C. zanthoxyli/AFC266F776971C0212DA71B50E9A8BB7.jpg]

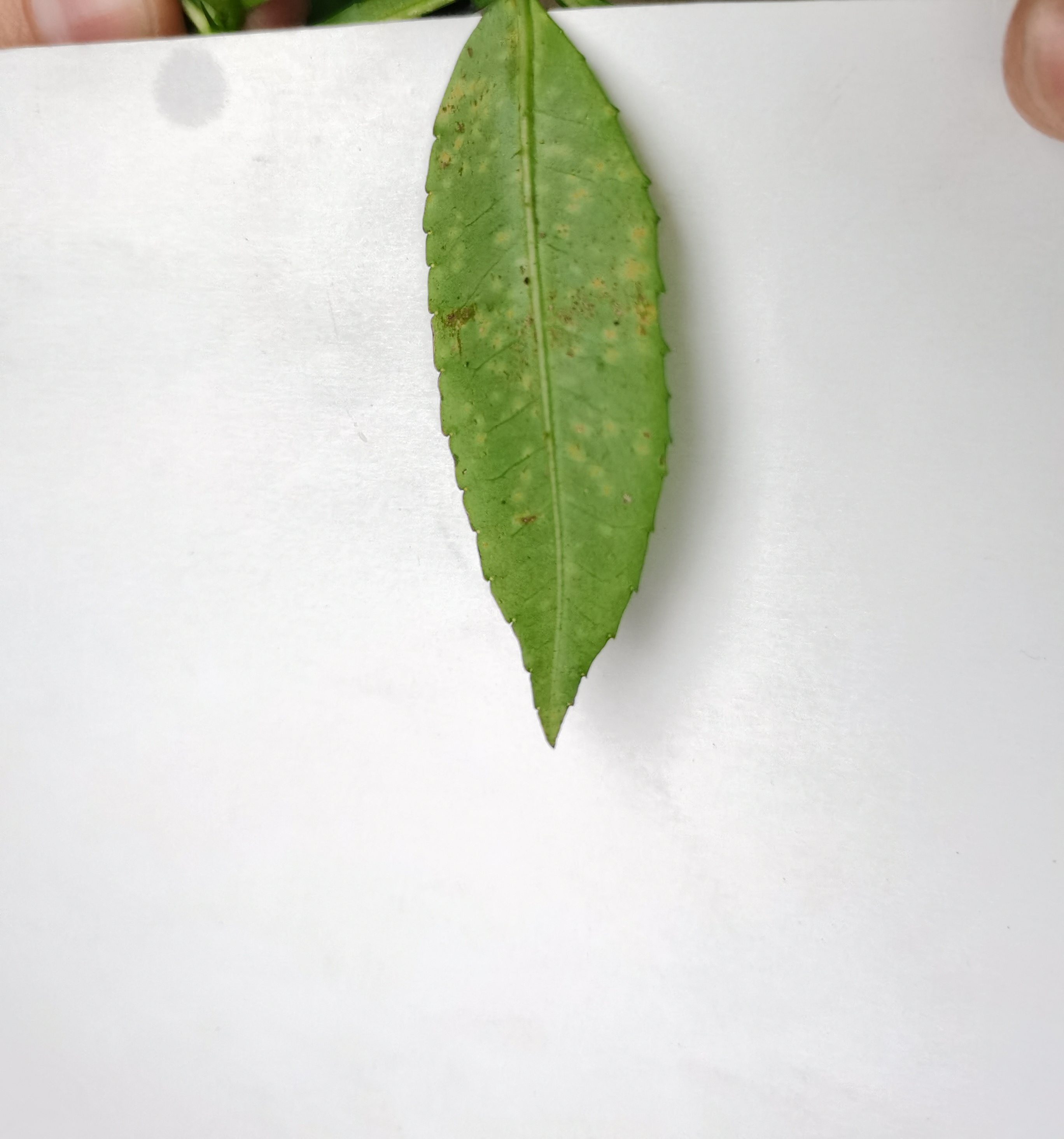

Supplement: Supplementary file 1 [file ijms-24-14761-s001.zip › Figure 1/Tengjiao-inoculated with C. zanthoxyli/IMG_20211020_174713_edit_389658816311895.jpg]

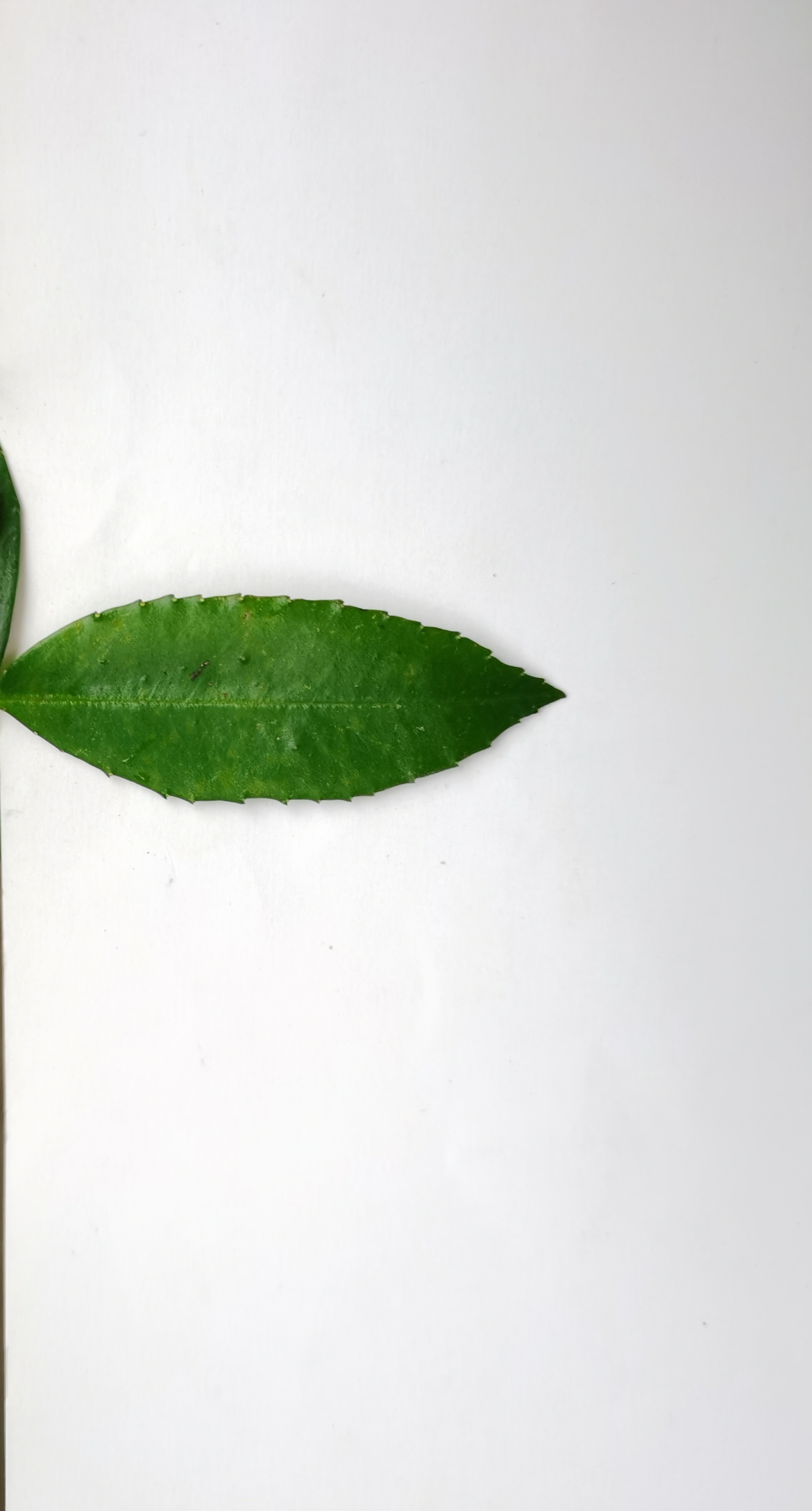

Supplement: Supplementary file 1 [file ijms-24-14761-s001.zip › Figure 1/Tengjiao-inoculated with C. zanthoxyli/IMG_20211020_174726_edit_389666934978561.jpg]

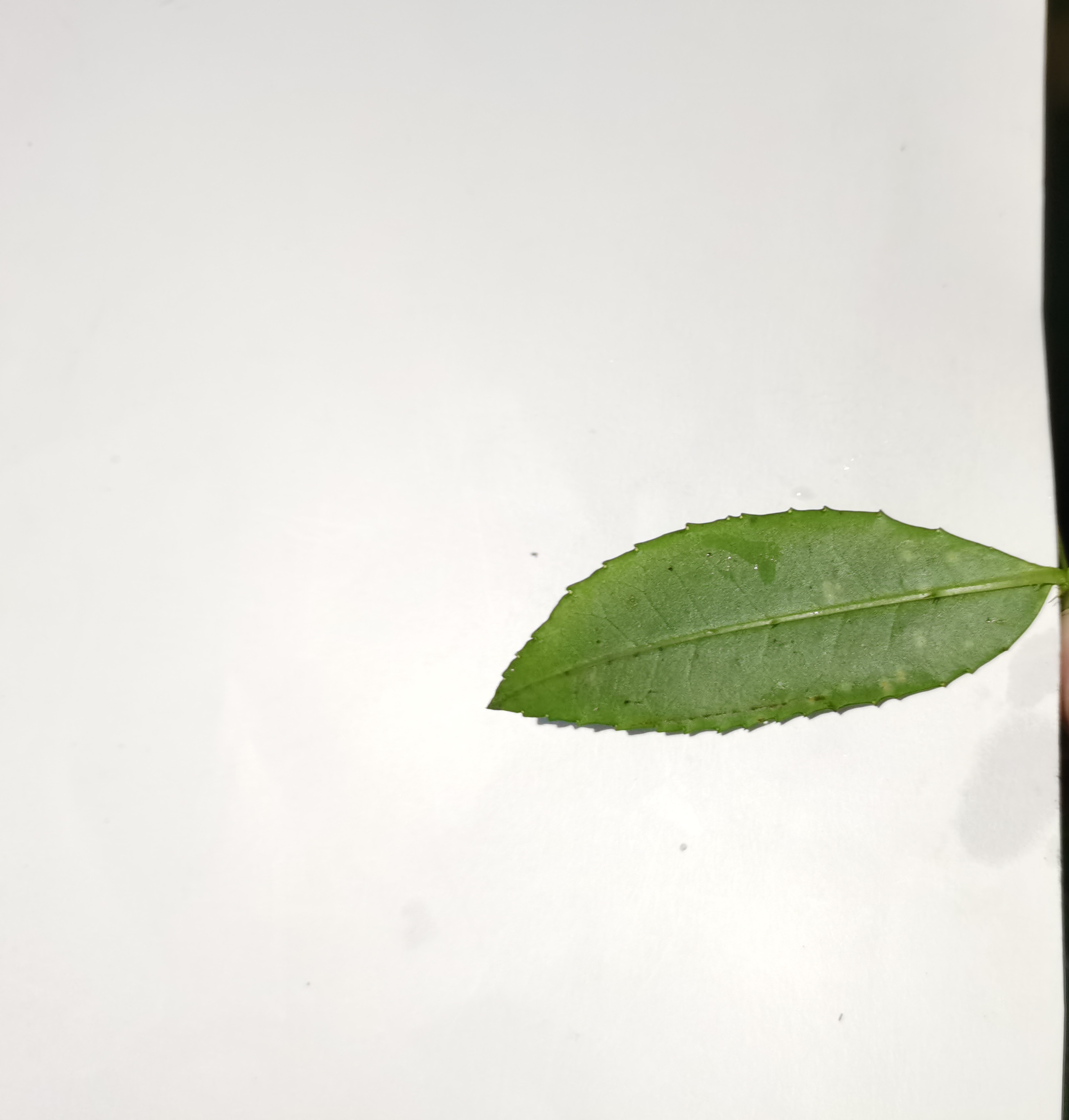

Supplement: Supplementary file 1 [file ijms-24-14761-s001.zip › Figure 1/Tengjiao-inoculated with C. zanthoxyli/IMG_20211021_183444_edit_387043136121669.jpg]

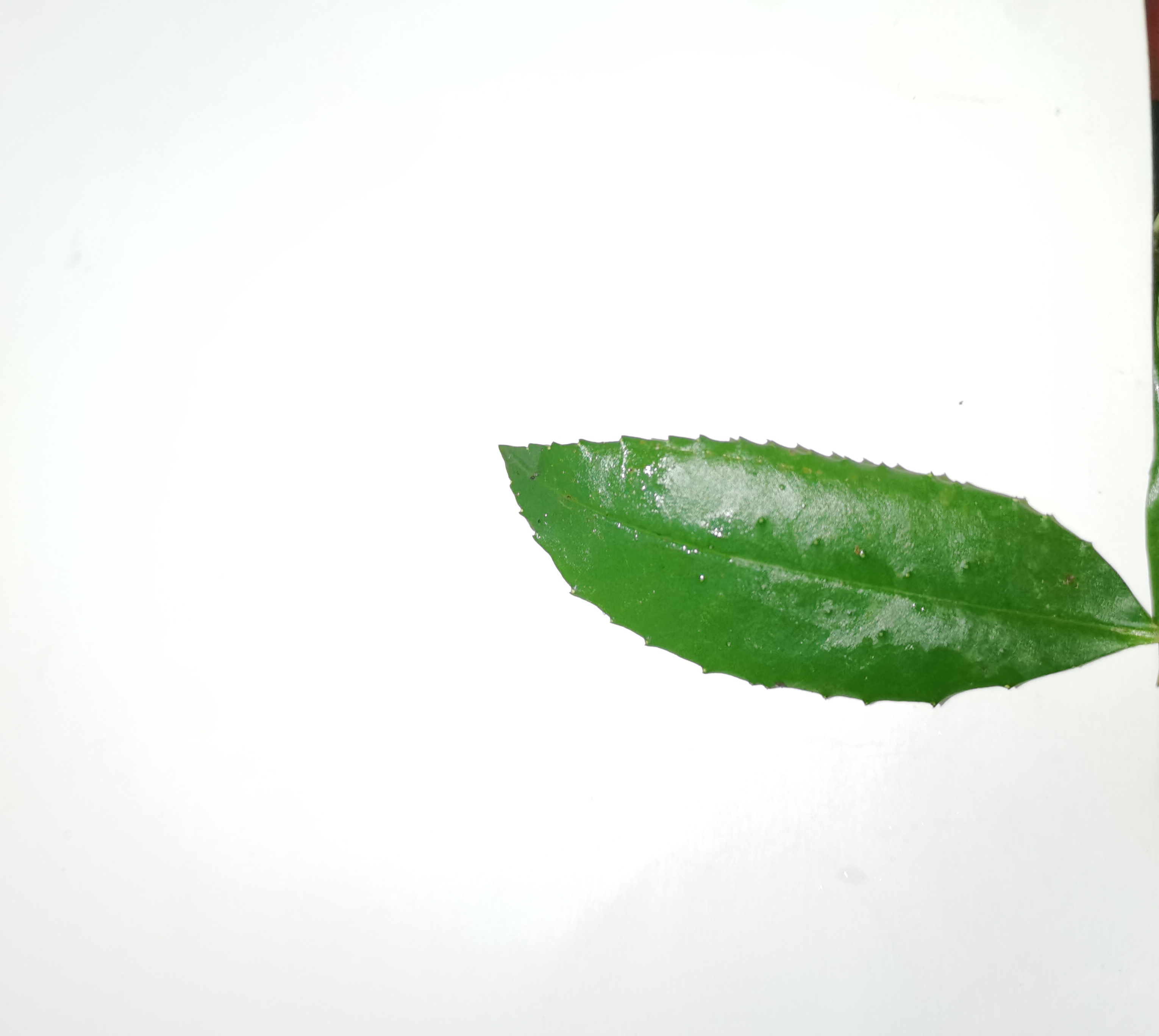

Supplement: Supplementary file 1 [file ijms-24-14761-s001.zip › Figure 1/Tengjiao-inoculated with C. zanthoxyli/IMG_20211021_183502_edit_386989894986261.jpg]

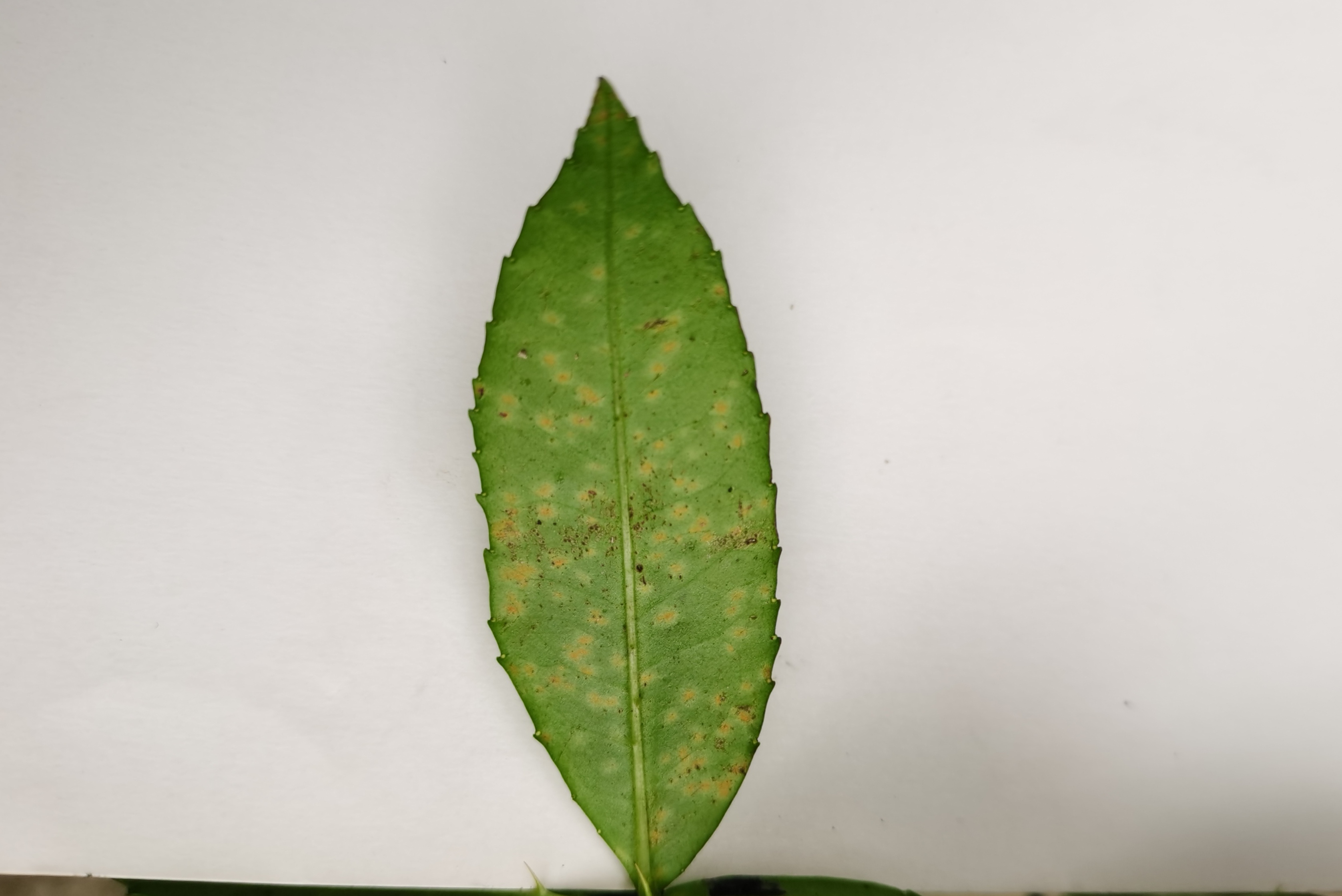

Supplement: Supplementary file 1 [file ijms-24-14761-s001.zip › Figure 1/Tengjiao-inoculated with C. zanthoxyli/IMG_20211022_154047_edit_393522321193597.jpg]

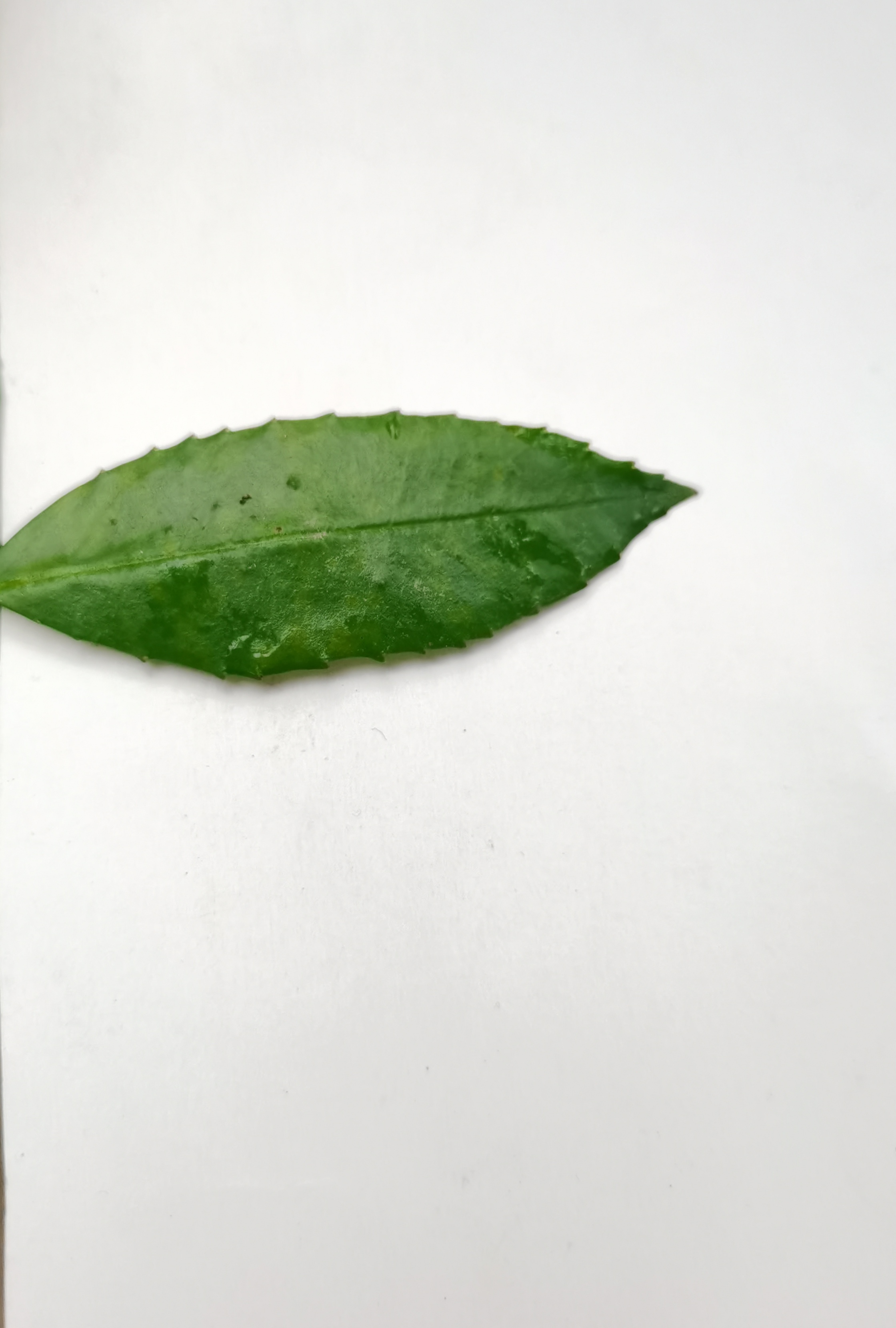

Supplement: Supplementary file 1 [file ijms-24-14761-s001.zip › Figure 1/Tengjiao-inoculated with C. zanthoxyli/IMG_20211023_172754_edit_431810847100776.jpg]

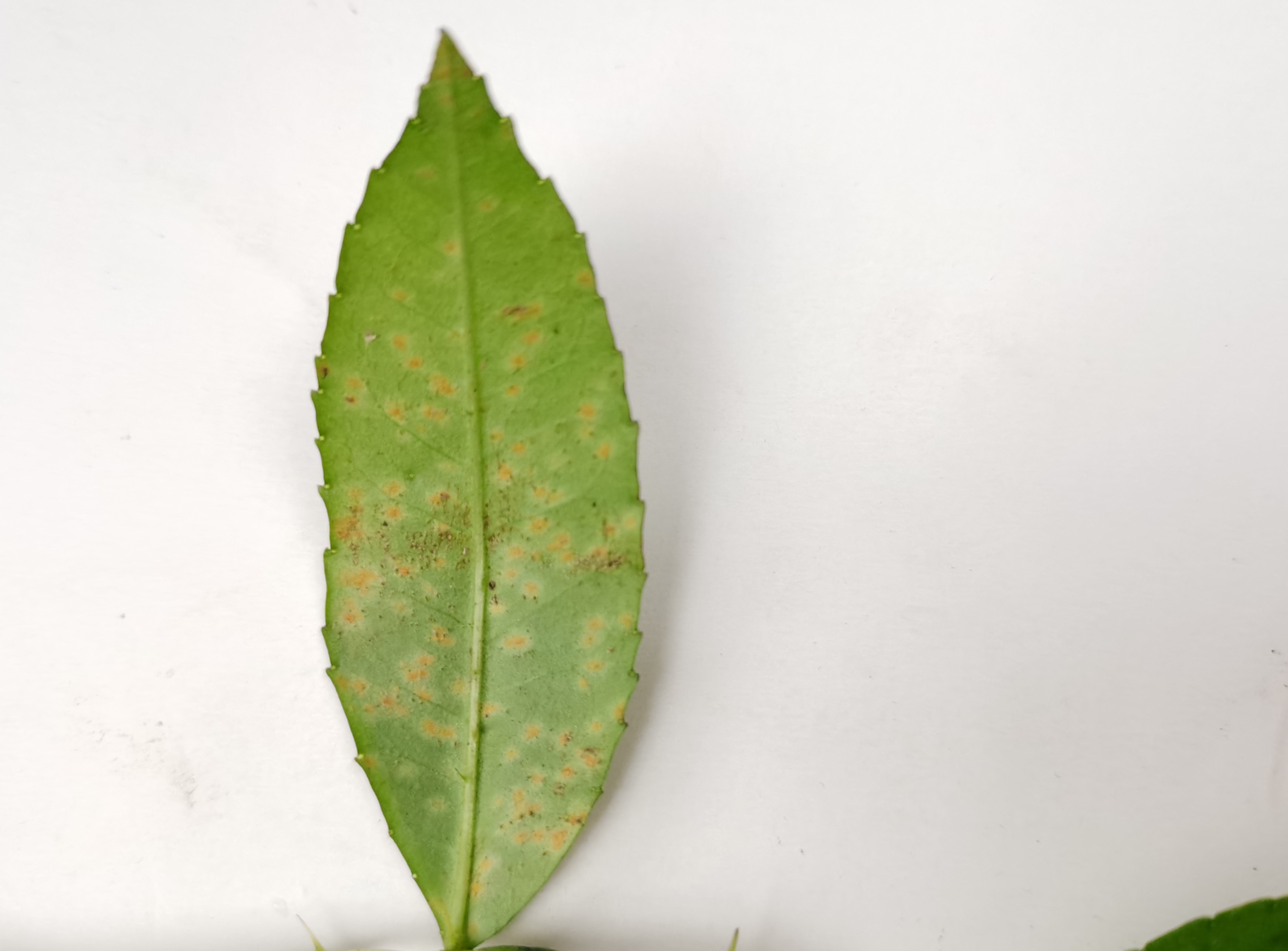

Supplement: Supplementary file 1 [file ijms-24-14761-s001.zip › Figure 1/Tengjiao-inoculated with C. zanthoxyli/IMG_20211023_172812_edit_431832443109627.jpg]

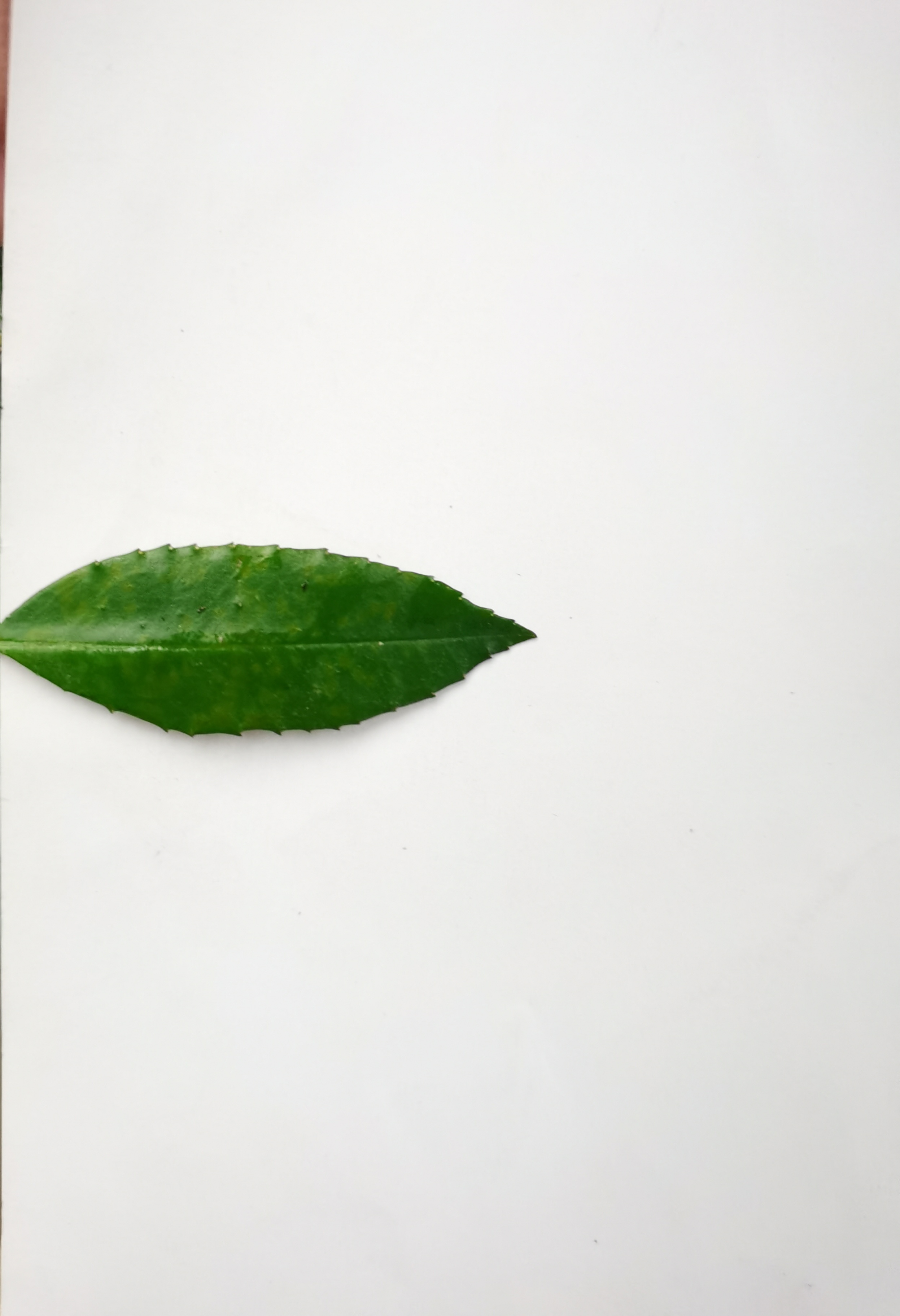

Supplement: Supplementary file 1 [file ijms-24-14761-s001.zip › Figure 1/Tengjiao-inoculated with C. zanthoxyli/IMG_20211024_175035_edit_472943674475749.jpg]

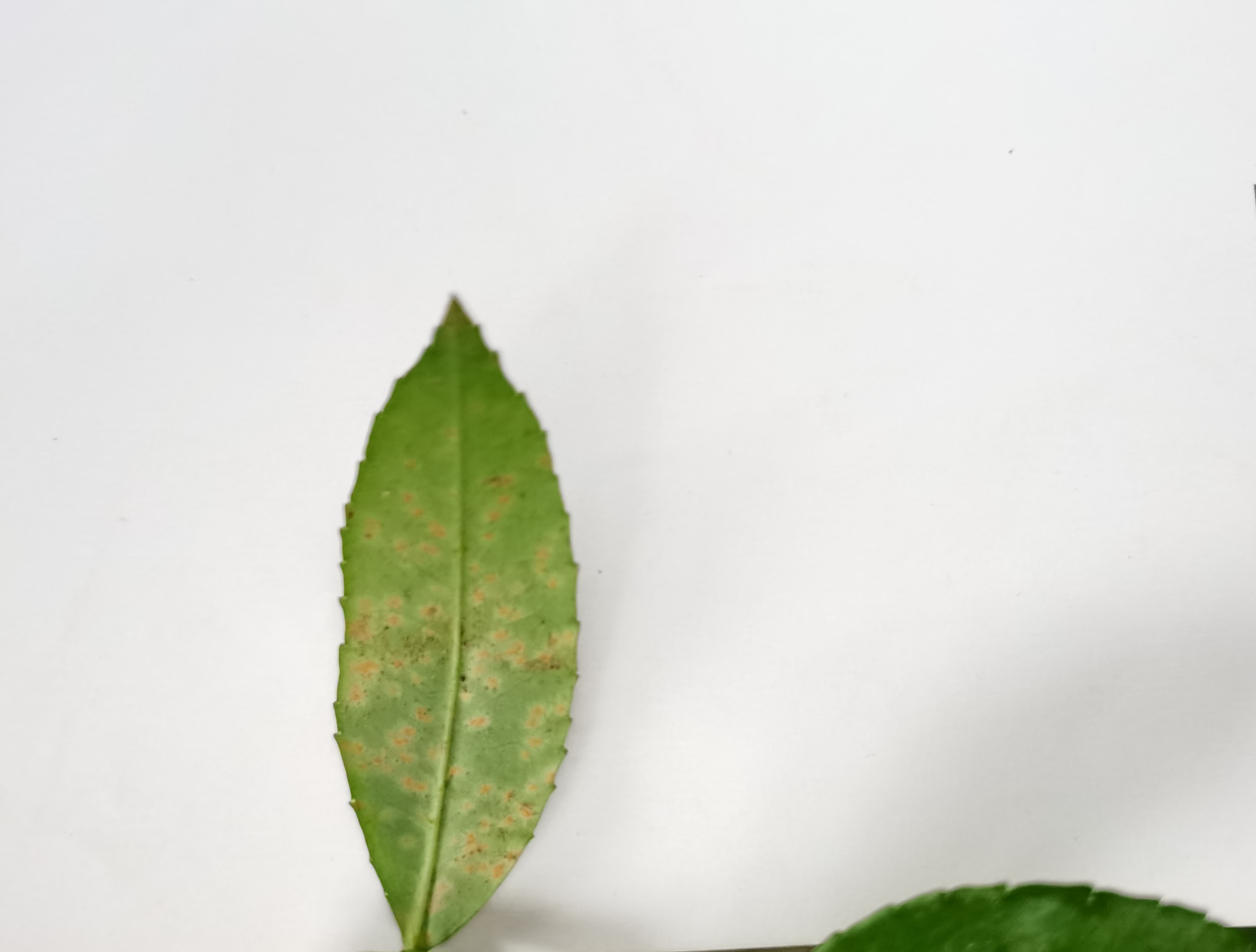

Supplement: Supplementary file 1 [file ijms-24-14761-s001.zip › Figure 1/Tengjiao-inoculated with C. zanthoxyli/IMG_20211024_175143_edit_472952113234081.jpg]

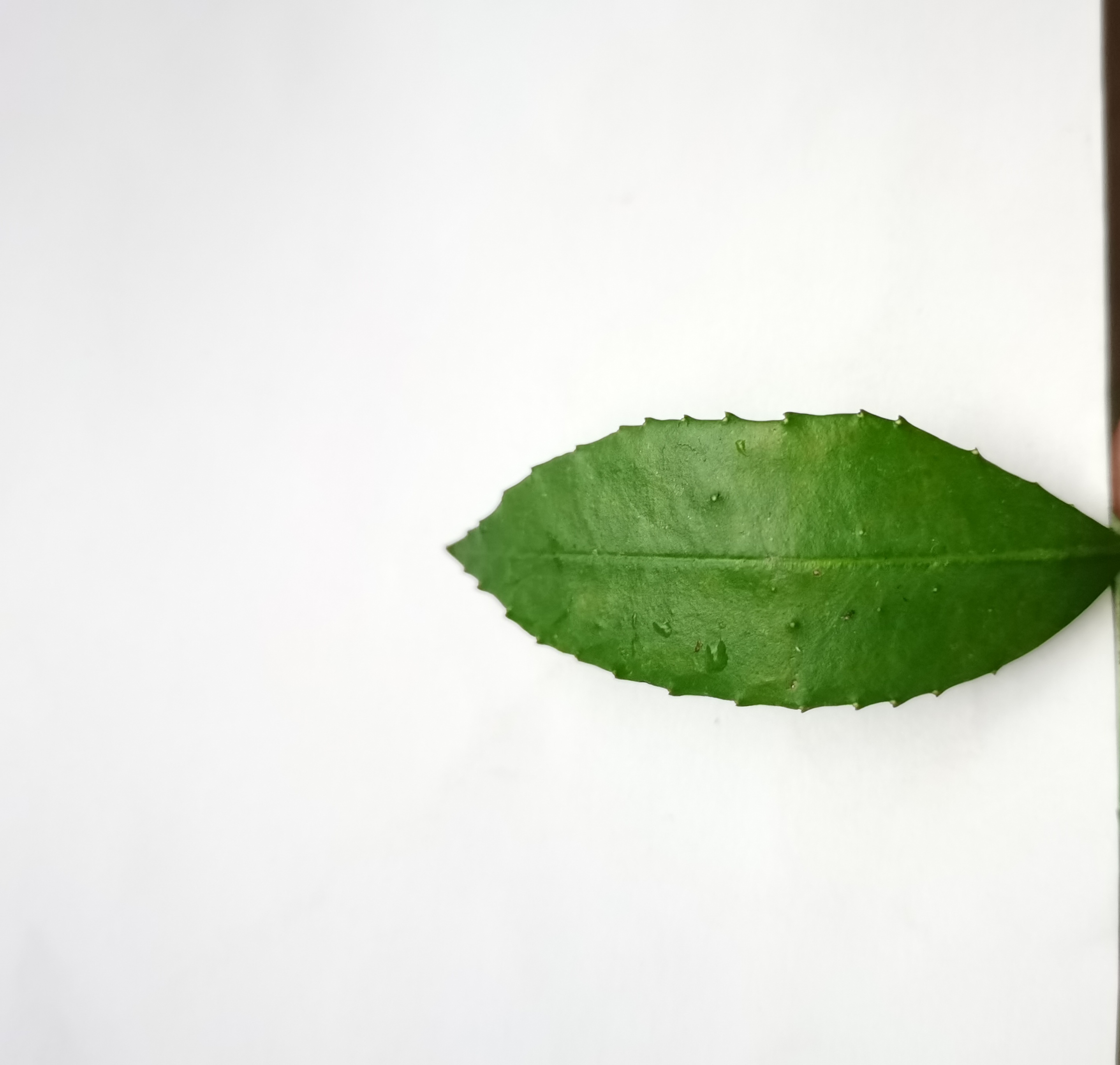

Supplement: Supplementary file 1 [file ijms-24-14761-s001.zip › Figure 1/Tengjiao-inoculated with C. zanthoxyli/IMG_20211025_175445_edit_482963312397658.jpg]

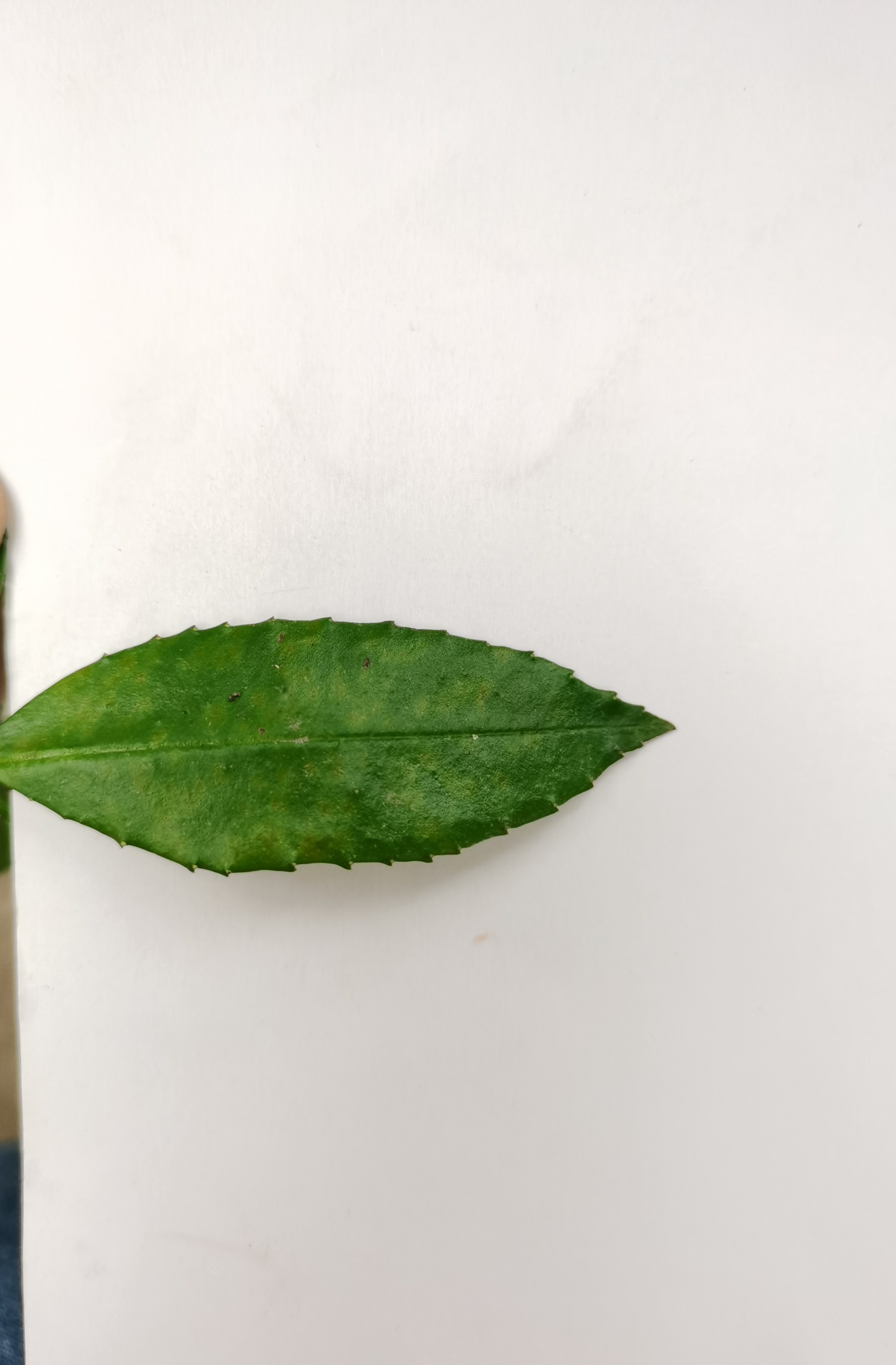

Supplement: Supplementary file 1 [file ijms-24-14761-s001.zip › Figure 1/Tengjiao-inoculated with C. zanthoxyli/IMG_20211026_165145_edit_512330058969219.jpg]

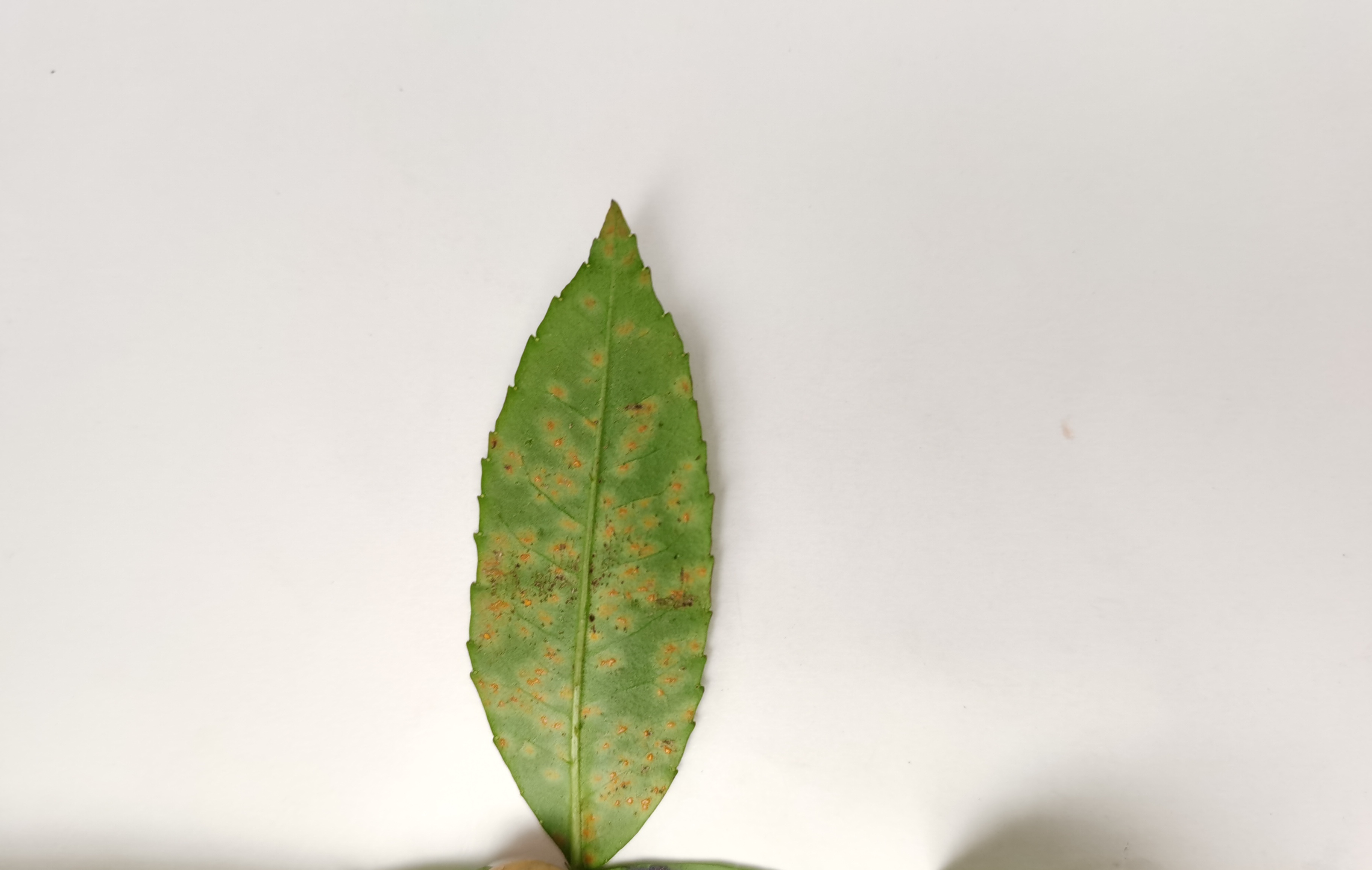

Supplement: Supplementary file 1 [file ijms-24-14761-s001.zip › Figure 1/Tengjiao-inoculated with C. zanthoxyli/IMG_20211026_165217_edit_512336469871822.jpg]

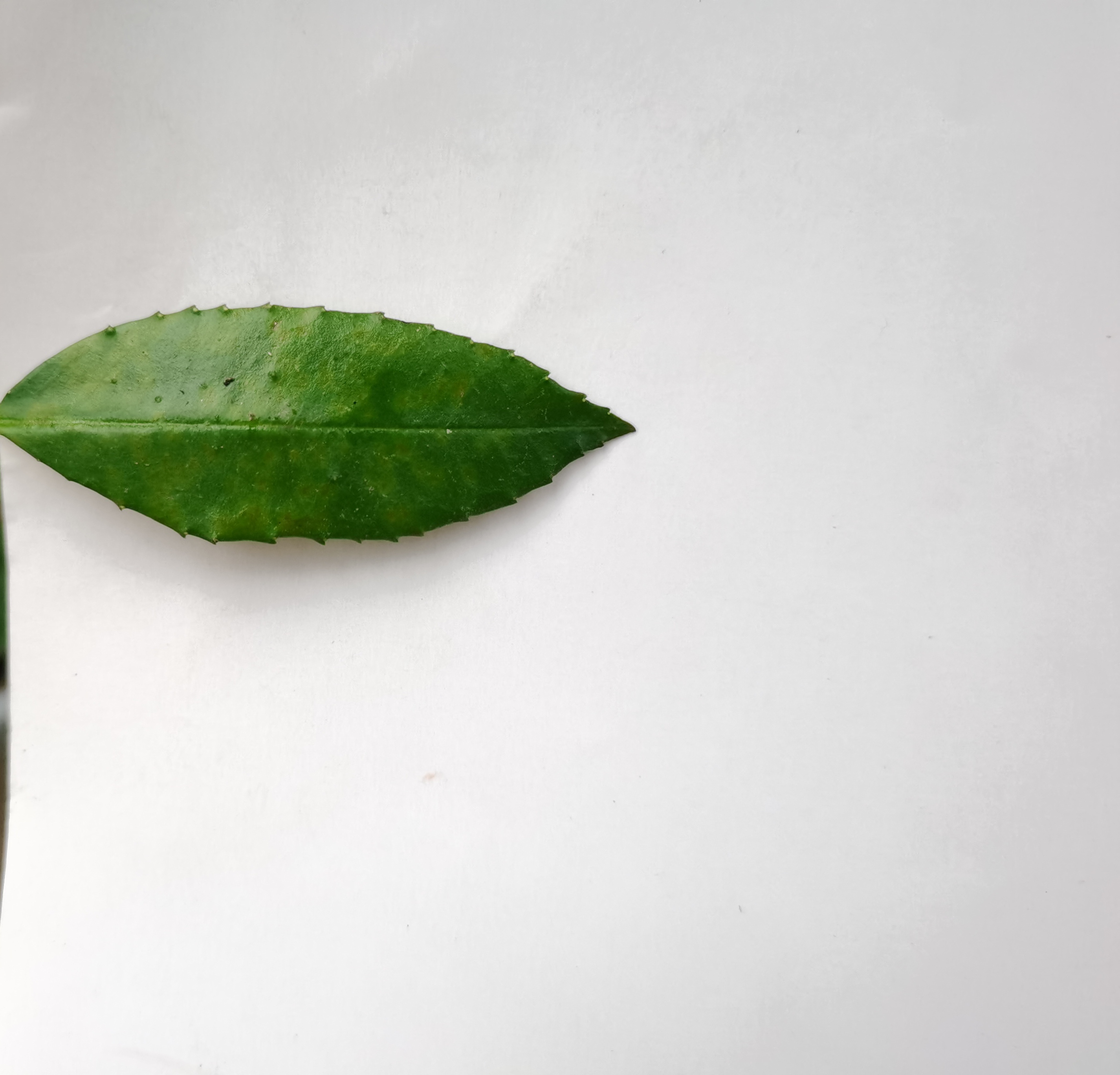

Supplement: Supplementary file 1 [file ijms-24-14761-s001.zip › Figure 1/Tengjiao-inoculated with C. zanthoxyli/IMG_20211027_173547_edit_543290519851994.jpg]

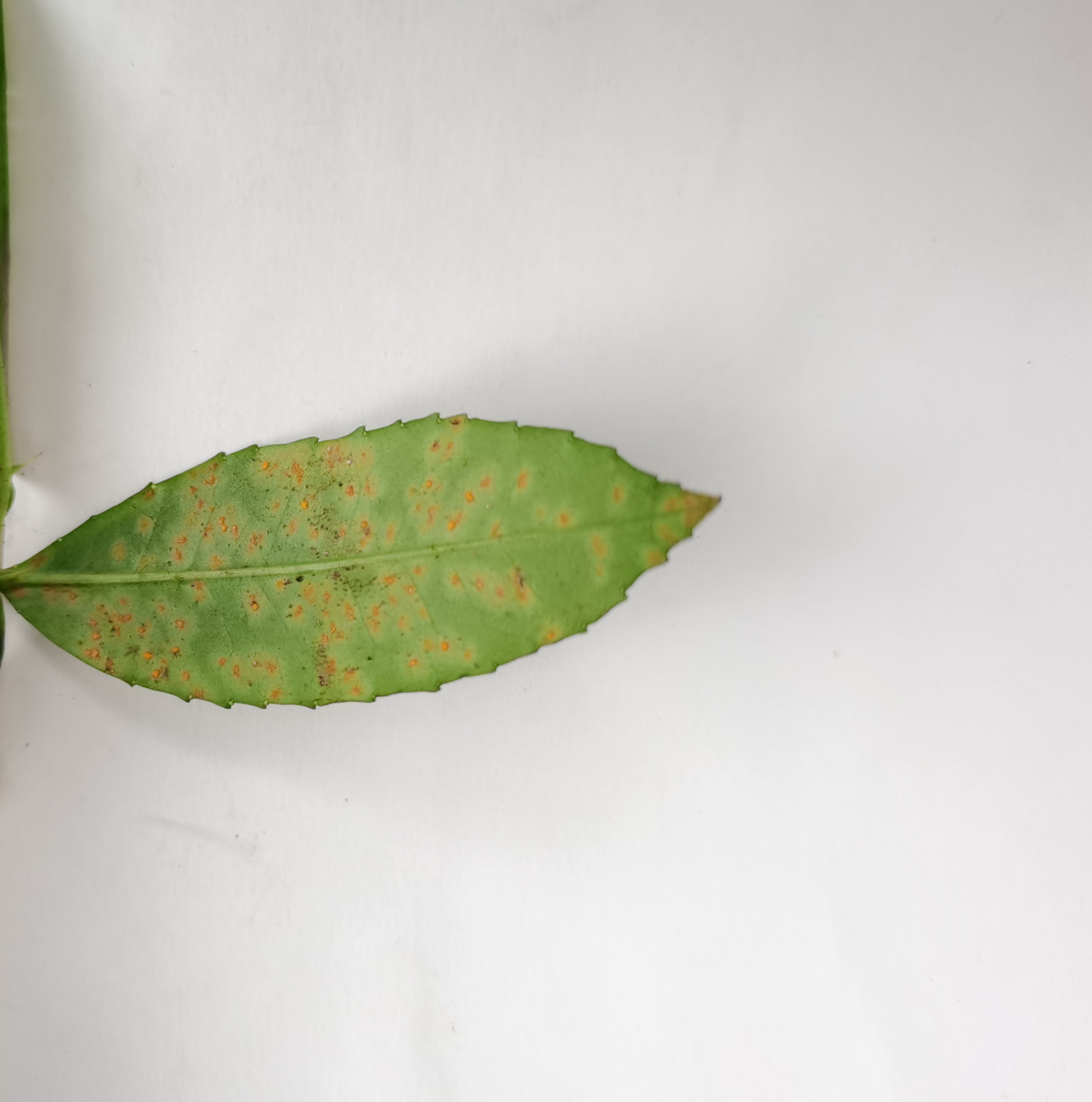

Supplement: Supplementary file 1 [file ijms-24-14761-s001.zip › Figure 1/Tengjiao-inoculated with C. zanthoxyli/IMG_20211027_173602_edit_543298755541056.jpg]

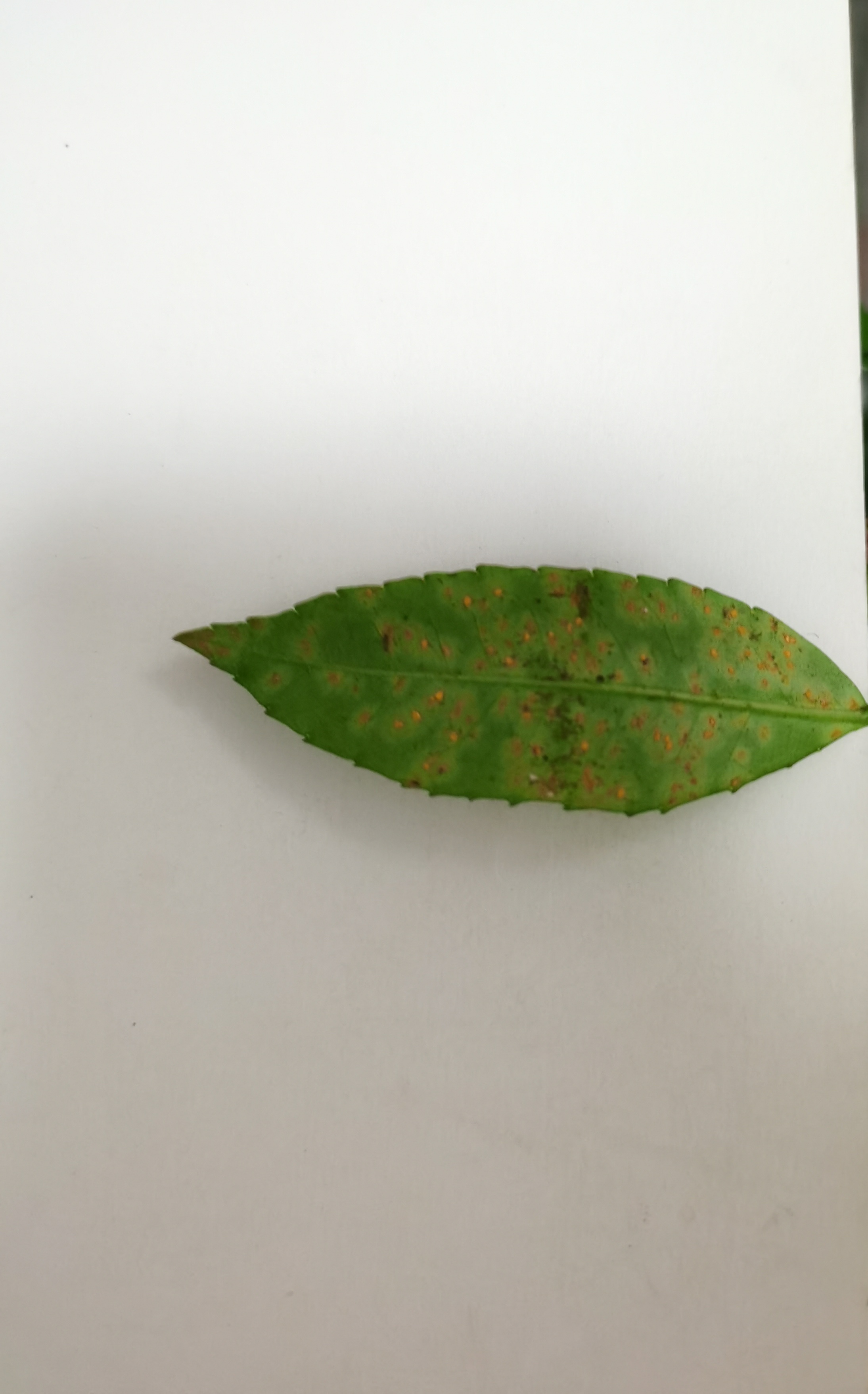

Supplement: Supplementary file 1 [file ijms-24-14761-s001.zip › Figure 1/Tengjiao-inoculated with C. zanthoxyli/IMG_20211028_190448_edit_603395173011573.jpg]

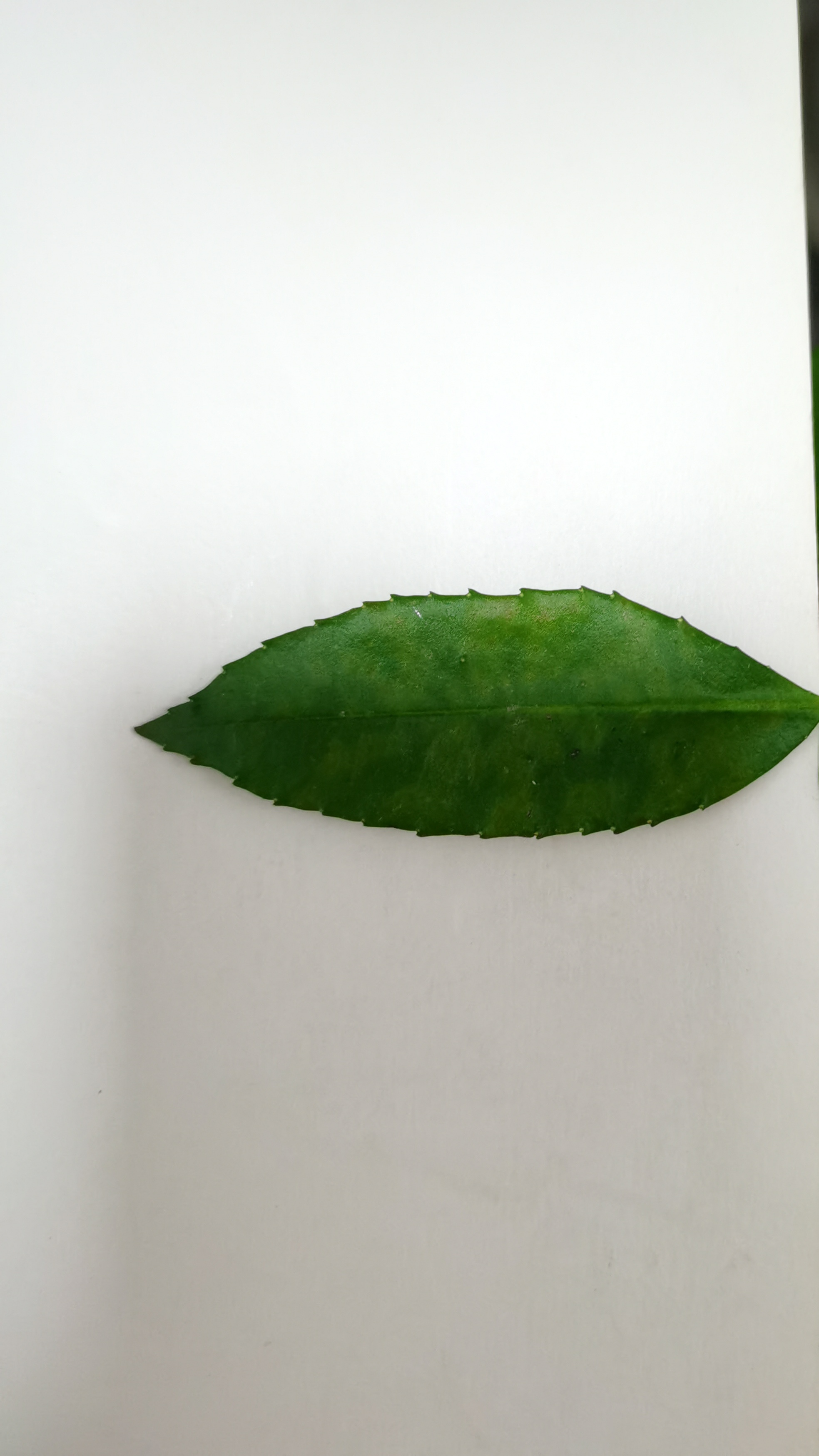

Supplement: Supplementary file 1 [file ijms-24-14761-s001.zip › Figure 1/Tengjiao-inoculated with C. zanthoxyli/IMG_20211028_190459_edit_603411566448550.jpg]

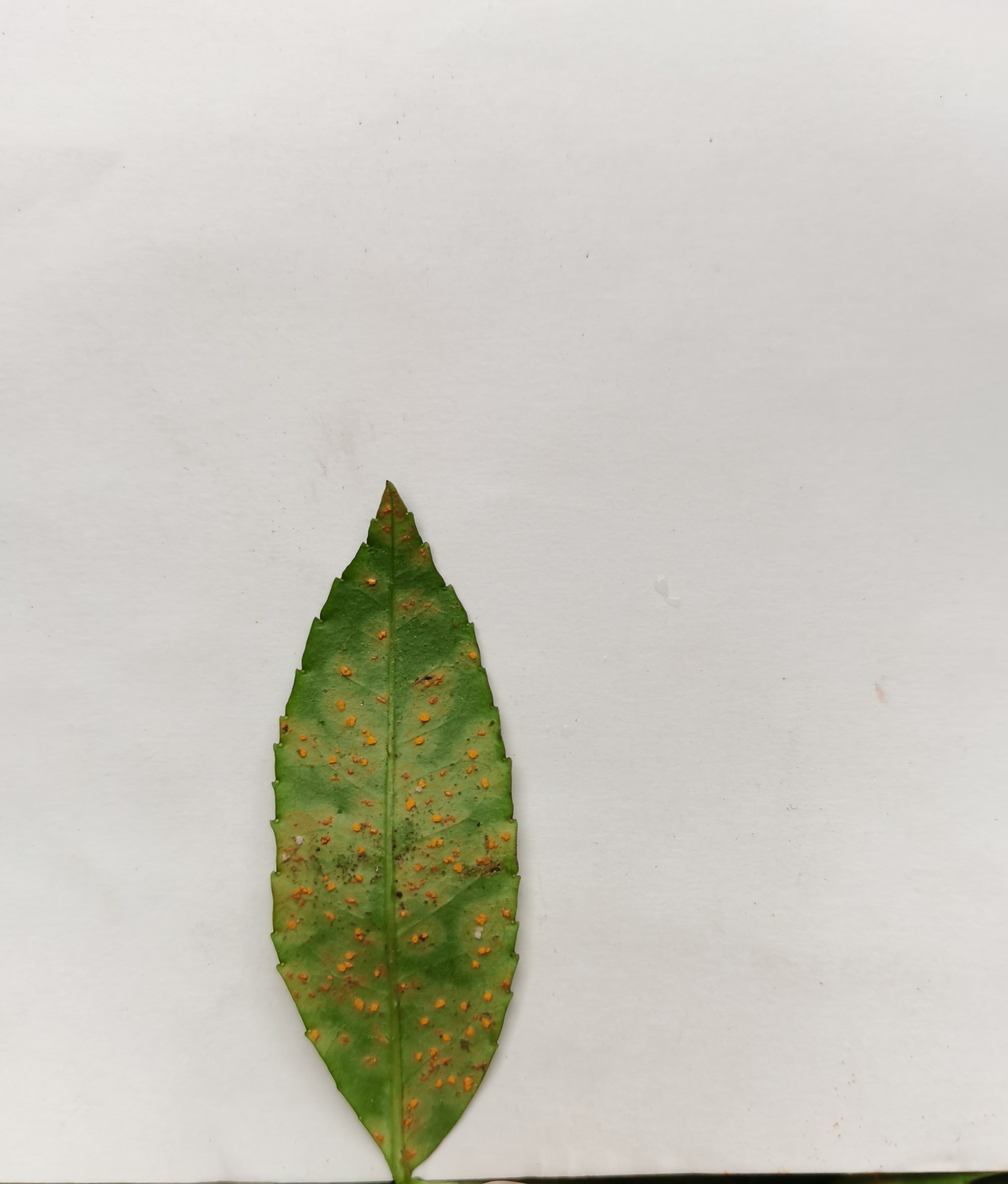

Supplement: Supplementary file 1 [file ijms-24-14761-s001.zip › Figure 1/Tengjiao-inoculated with C. zanthoxyli/IMG_20211030_173014_edit_644000797300689.jpg]

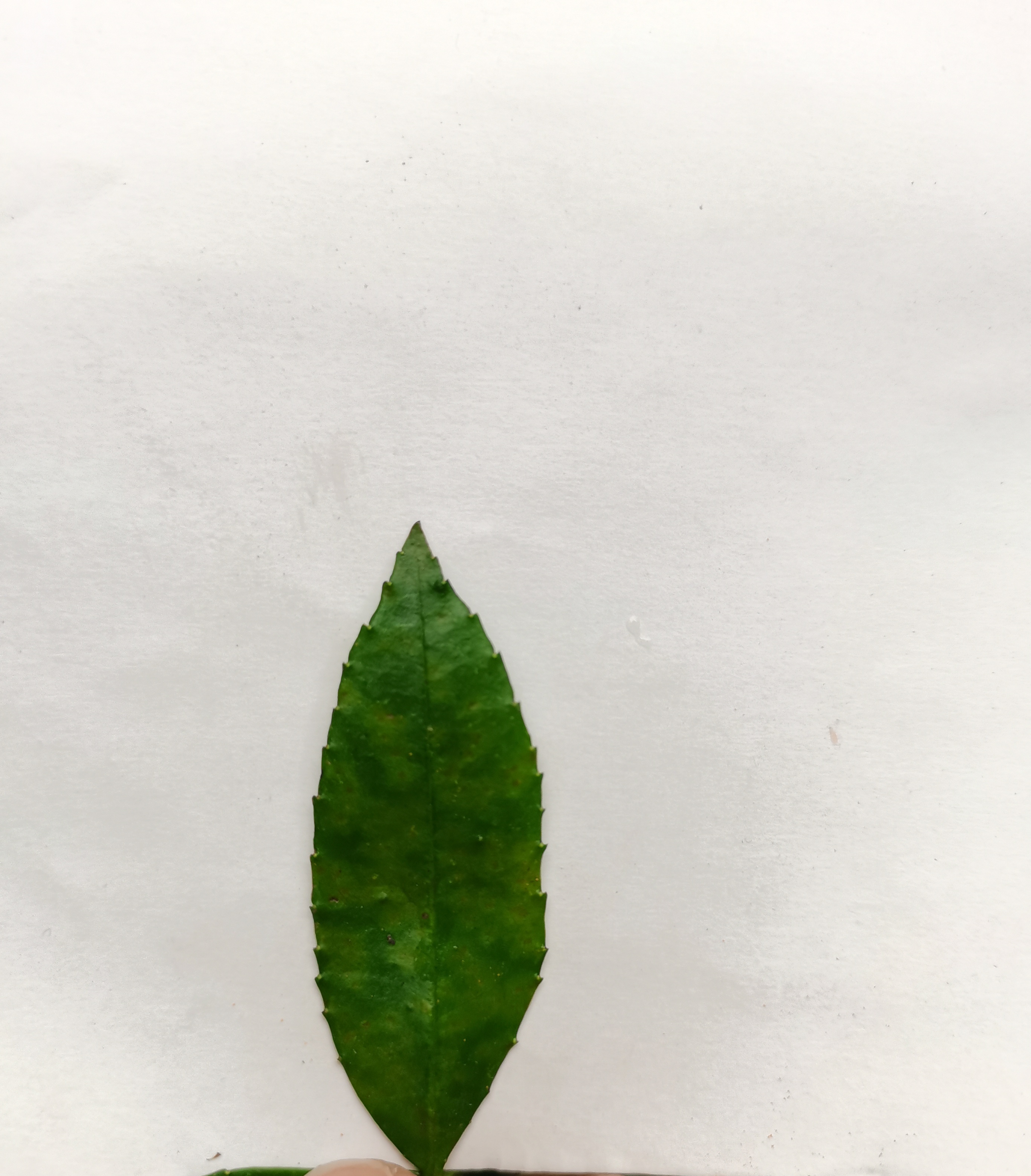

Supplement: Supplementary file 1 [file ijms-24-14761-s001.zip › Figure 1/Tengjiao-inoculated with C. zanthoxyli/IMG_20211030_173043_edit_644006695002251.jpg]

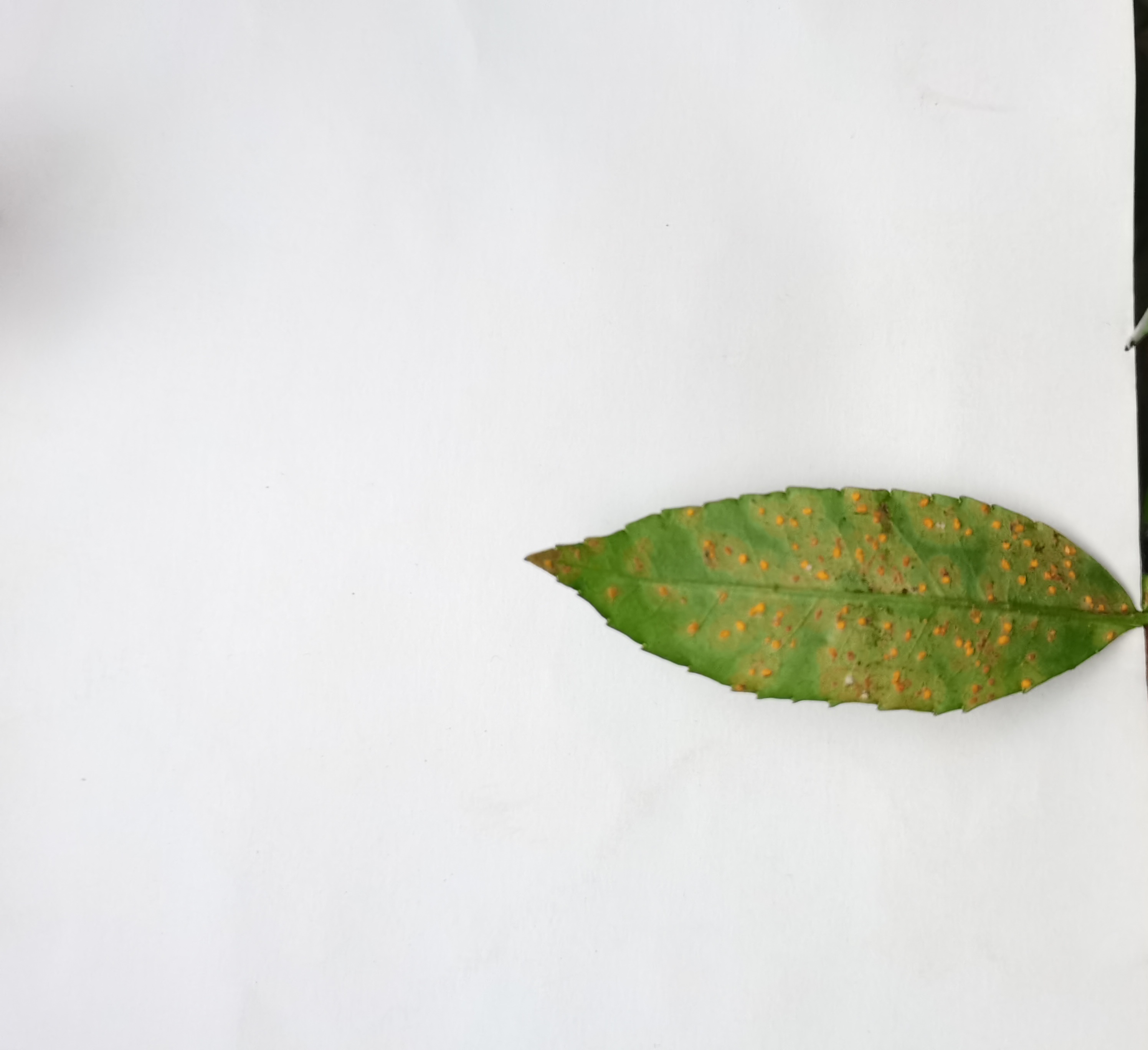

Supplement: Supplementary file 1 [file ijms-24-14761-s001.zip › Figure 1/Tengjiao-inoculated with C. zanthoxyli/IMG_20211031_180514_edit_673962167350283.jpg]

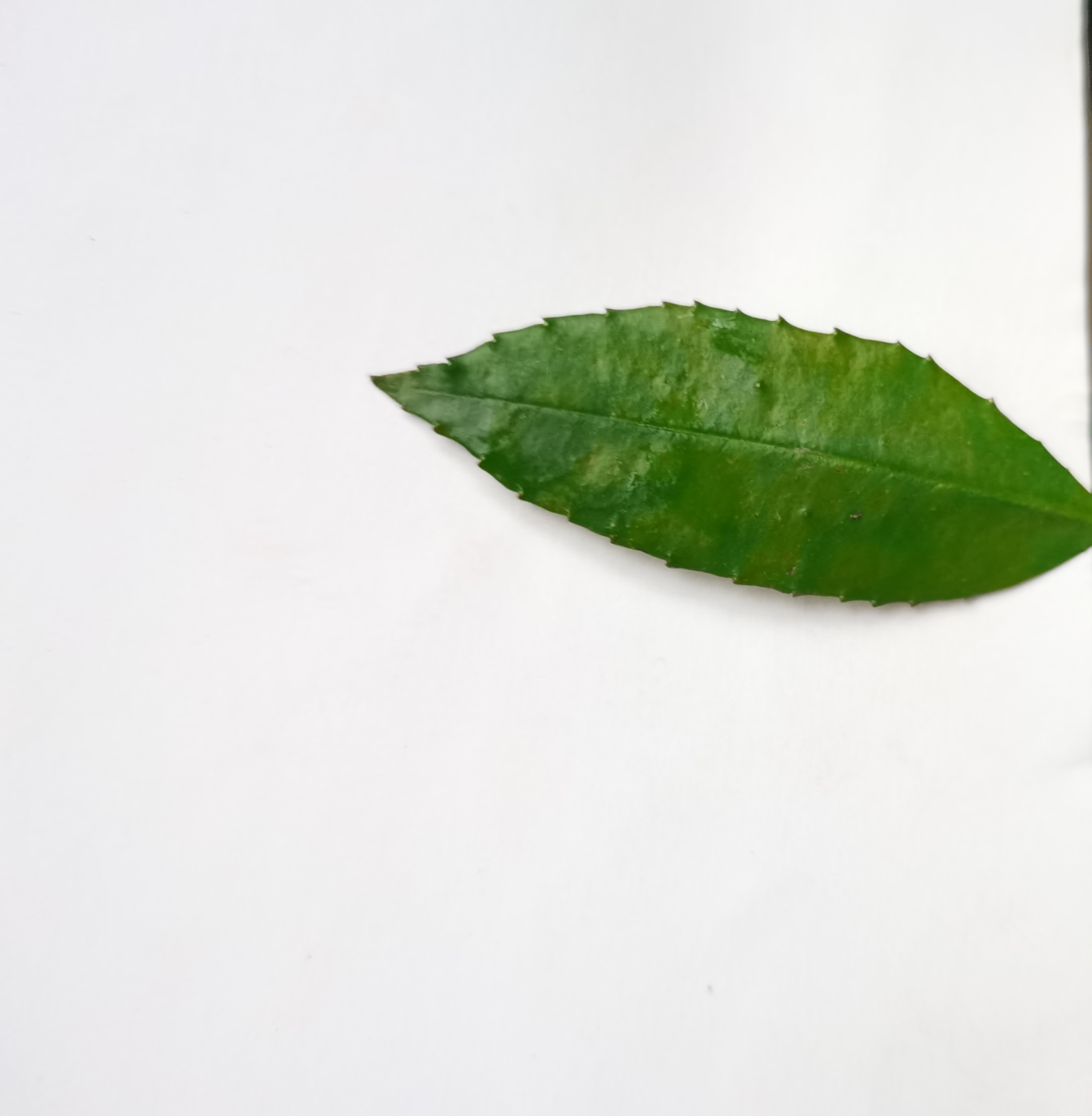

Supplement: Supplementary file 1 [file ijms-24-14761-s001.zip › Figure 1/Tengjiao-inoculated with C. zanthoxyli/IMG_20211031_180526_edit_673970459357053.jpg]

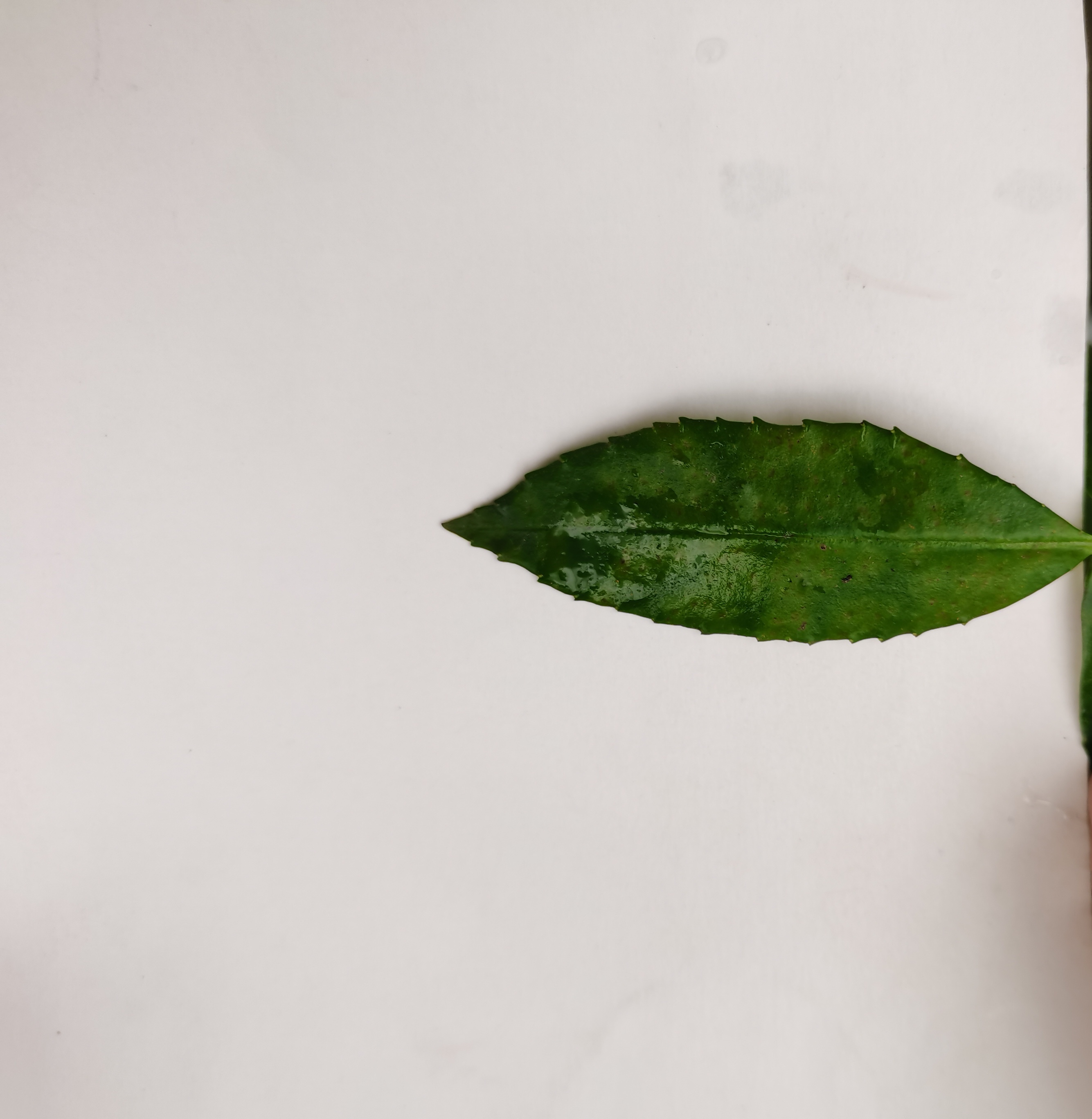

Supplement: Supplementary file 1 [file ijms-24-14761-s001.zip › Figure 1/Tengjiao-inoculated with C. zanthoxyli/IMG_20211101_172647_edit_702673874002673.jpg]

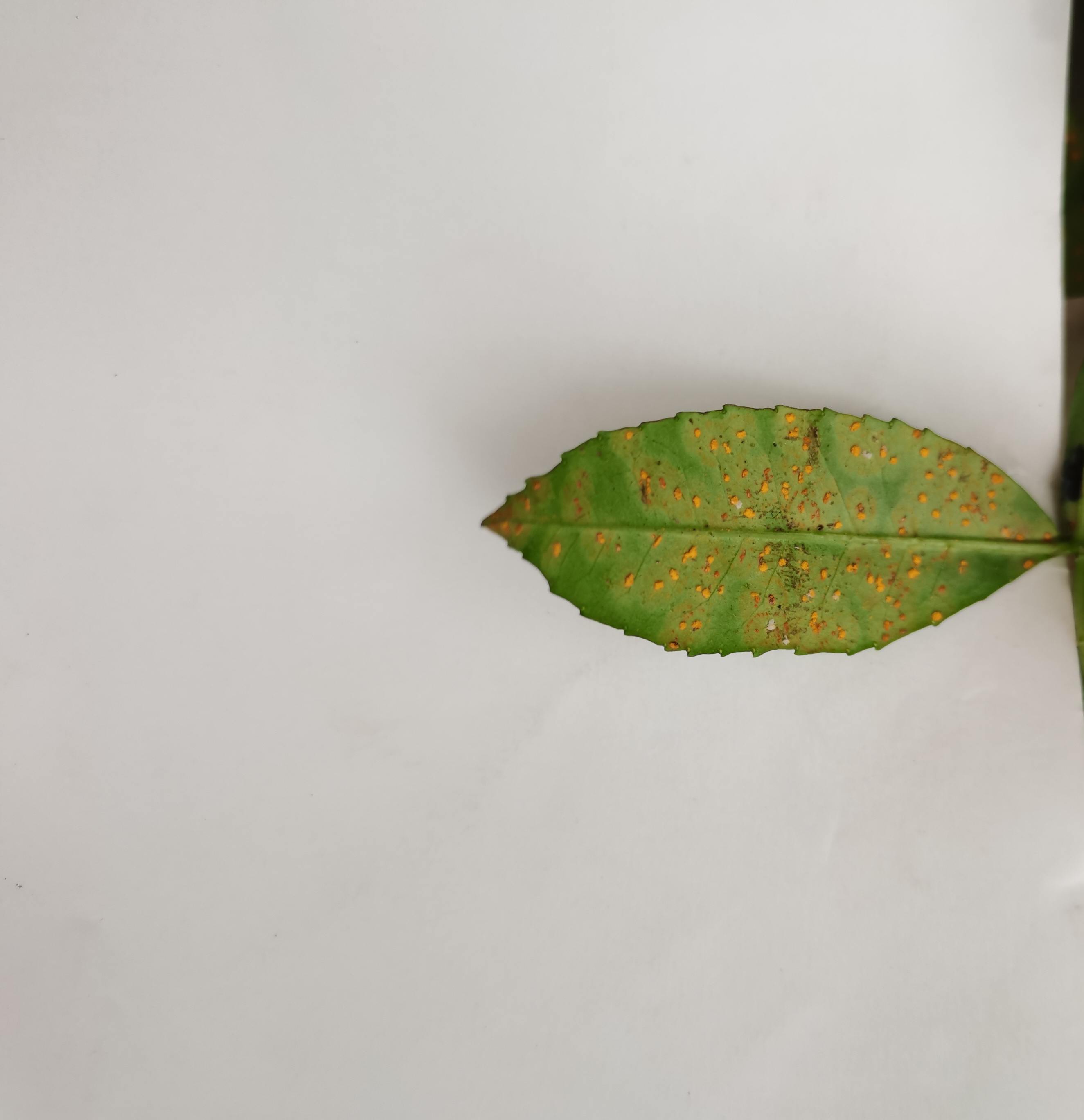

Supplement: Supplementary file 1 [file ijms-24-14761-s001.zip › Figure 1/Tengjiao-inoculated with C. zanthoxyli/IMG_20211101_172702_edit_702679850369339.jpg]

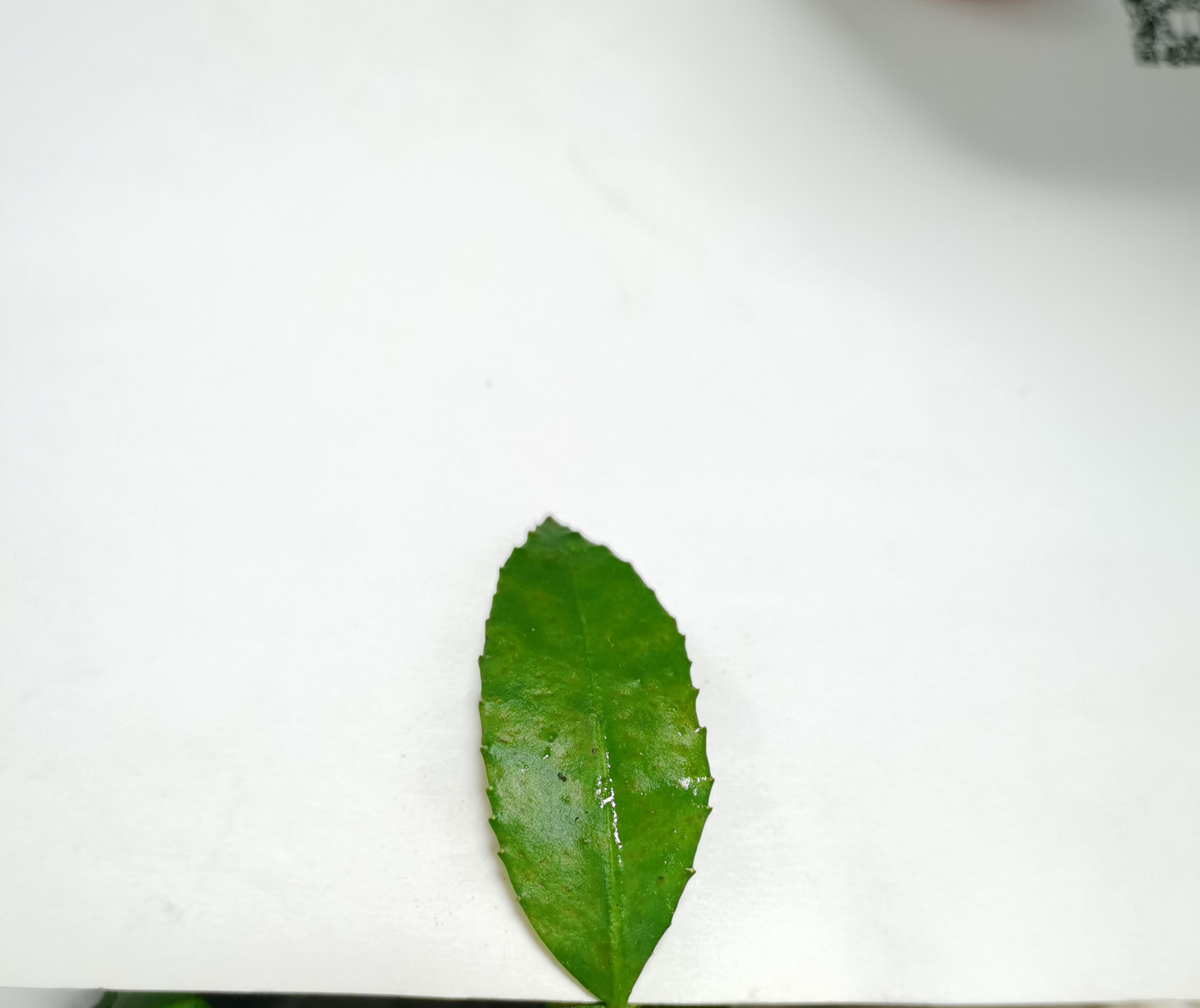

Supplement: Supplementary file 1 [file ijms-24-14761-s001.zip › Figure 1/Tengjiao-inoculated with C. zanthoxyli/IMG_20211102_220509_edit_760279332814717.jpg]

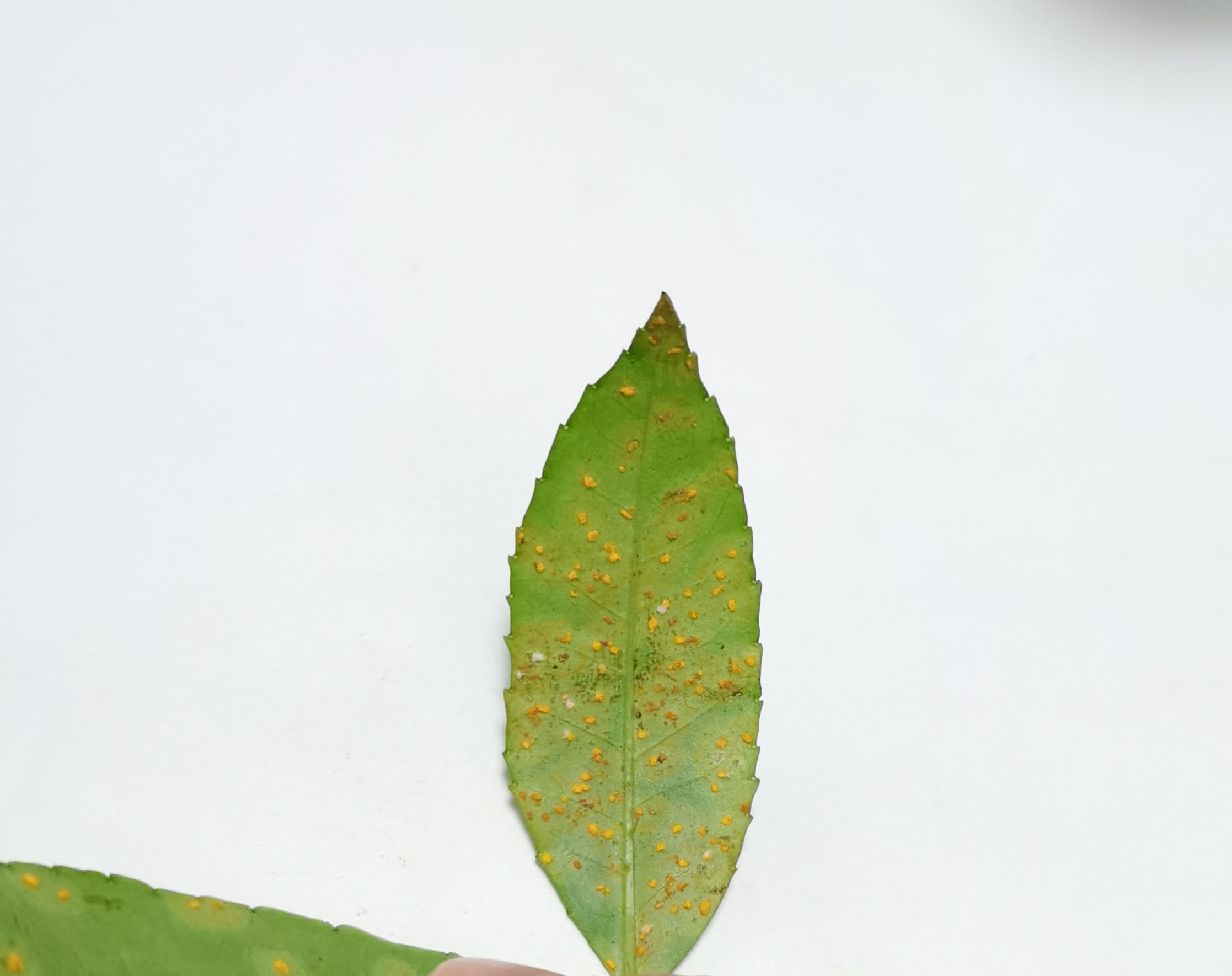

Supplement: Supplementary file 1 [file ijms-24-14761-s001.zip › Figure 1/Tengjiao-inoculated with C. zanthoxyli/IMG_20211102_220528_edit_760292107083465.jpg]

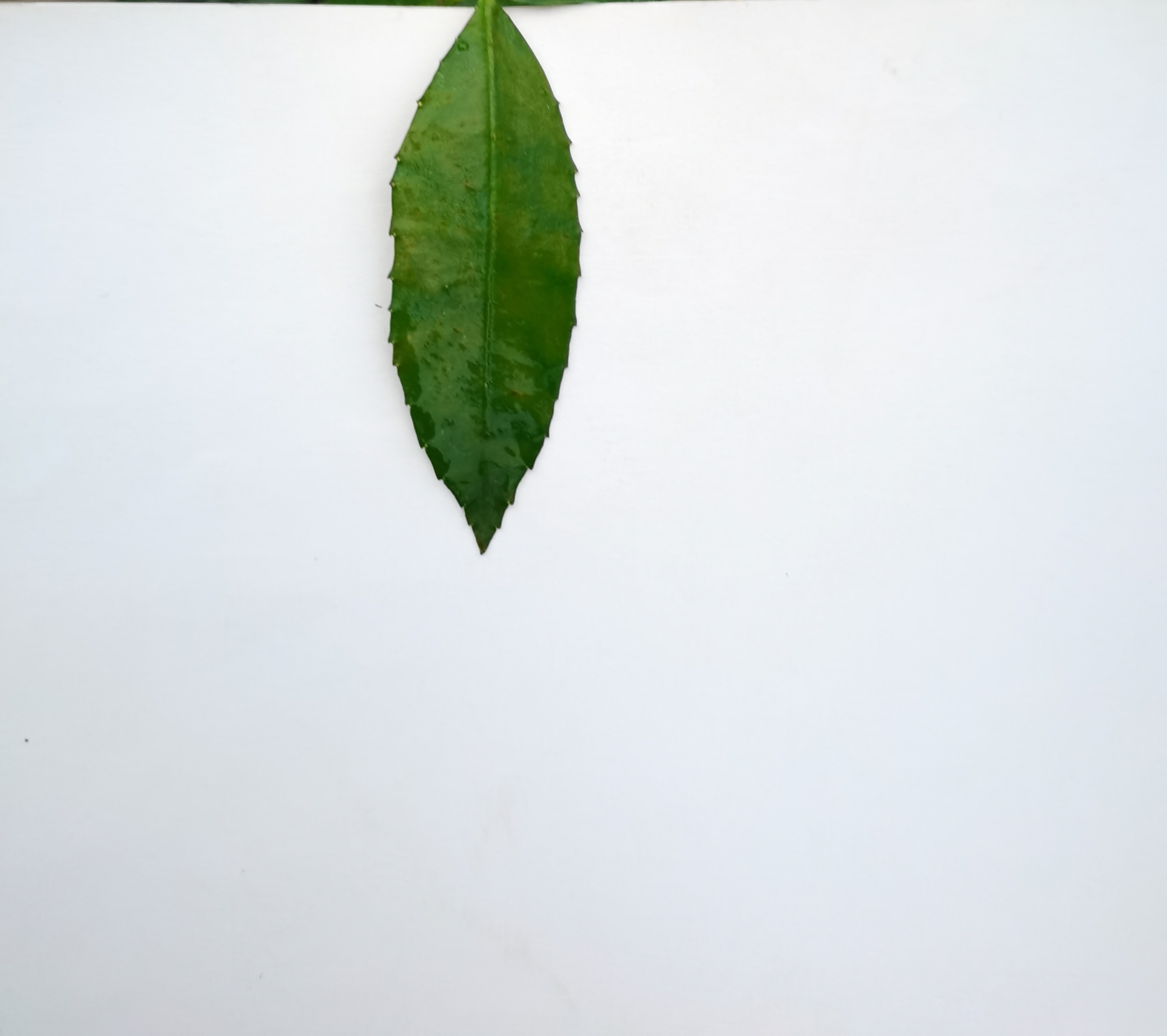

Supplement: Supplementary file 1 [file ijms-24-14761-s001.zip › Figure 1/Tengjiao-inoculated with C. zanthoxyli/IMG_20211103_181952_edit_819078758553661.jpg]

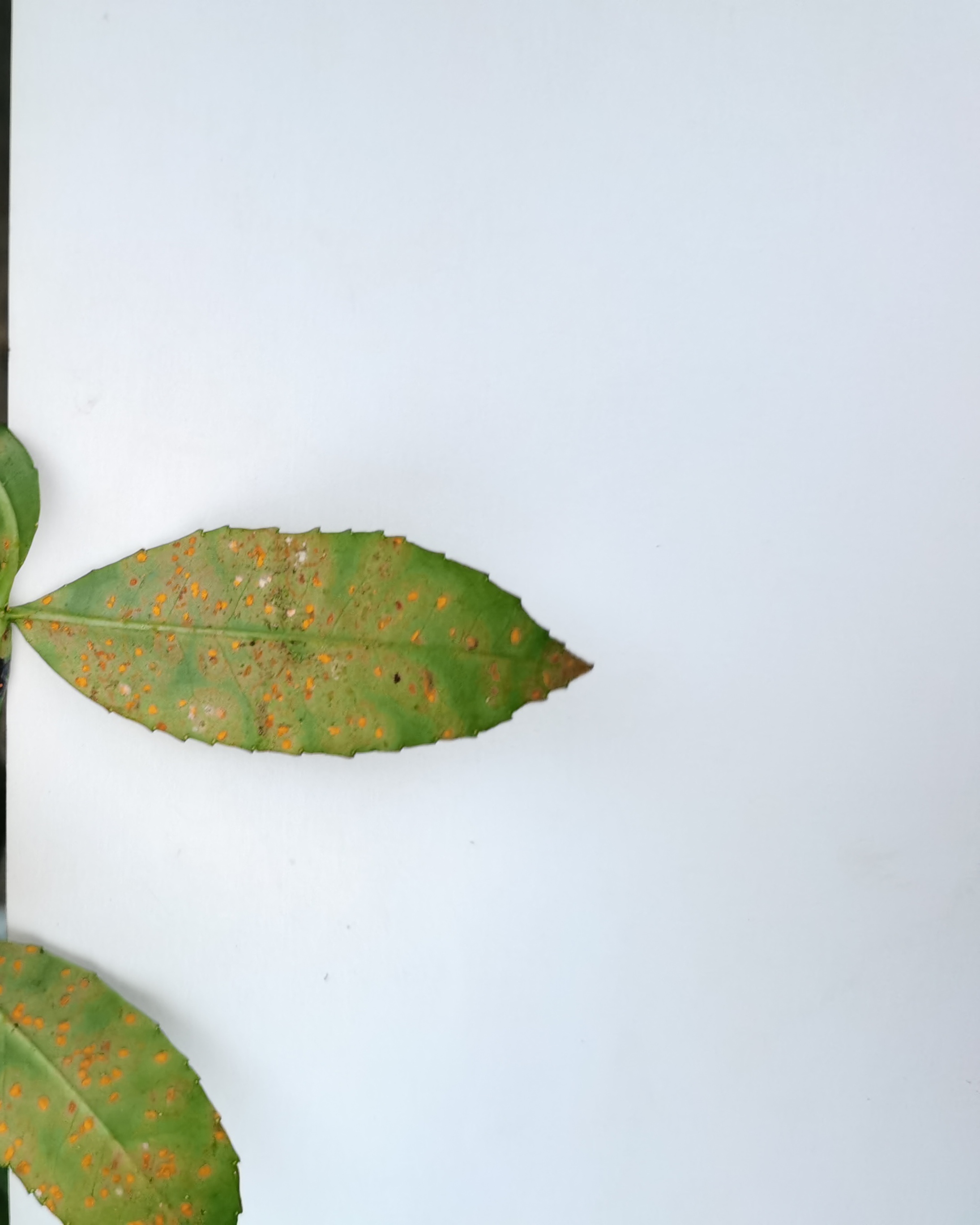

Supplement: Supplementary file 1 [file ijms-24-14761-s001.zip › Figure 1/Tengjiao-inoculated with C. zanthoxyli/IMG_20211103_182015_edit_819085806979702.jpg]

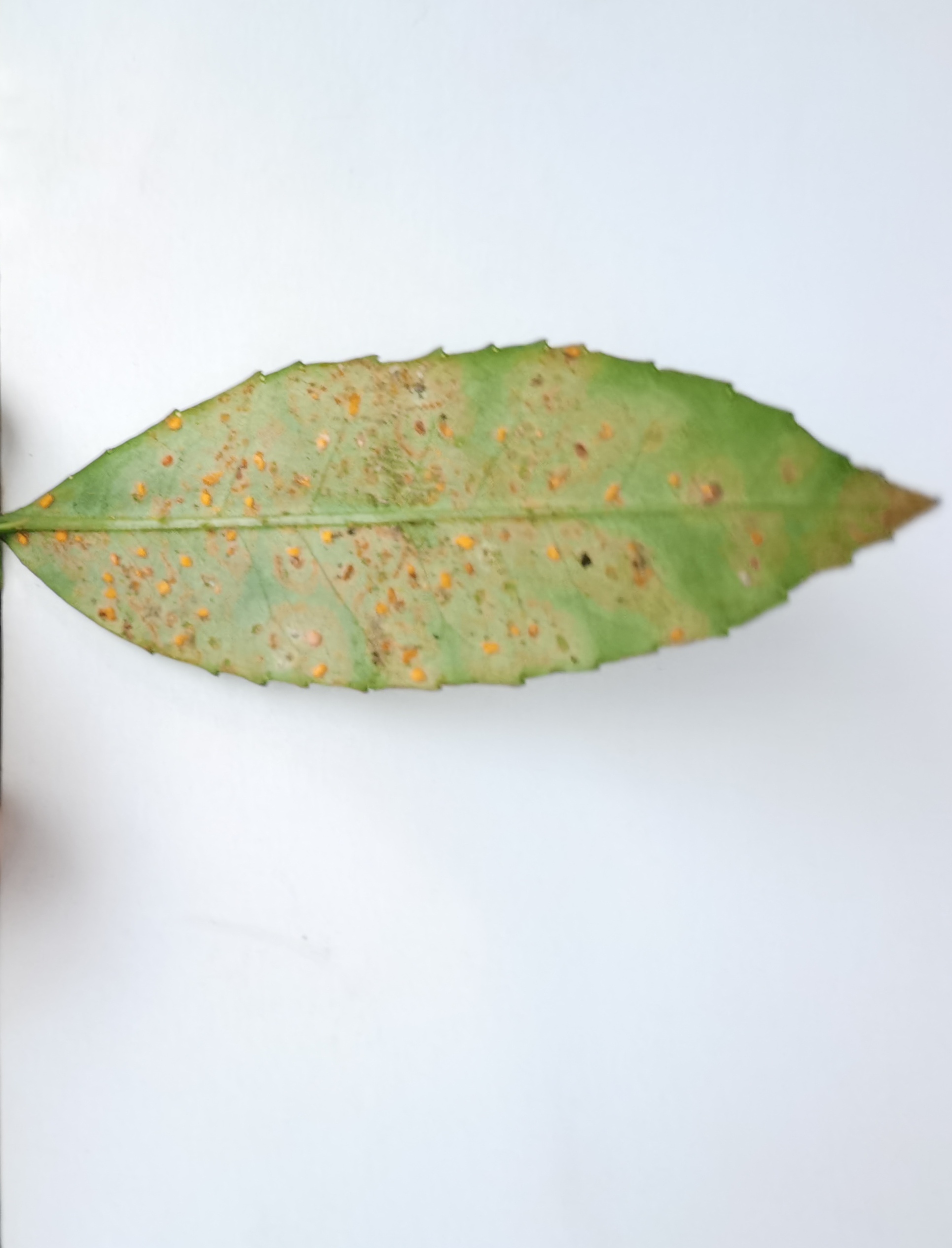

Supplement: Supplementary file 1 [file ijms-24-14761-s001.zip › Figure 1/Tengjiao-inoculated with C. zanthoxyli/IMG_20211104_175256_edit_819166887397919.jpg]

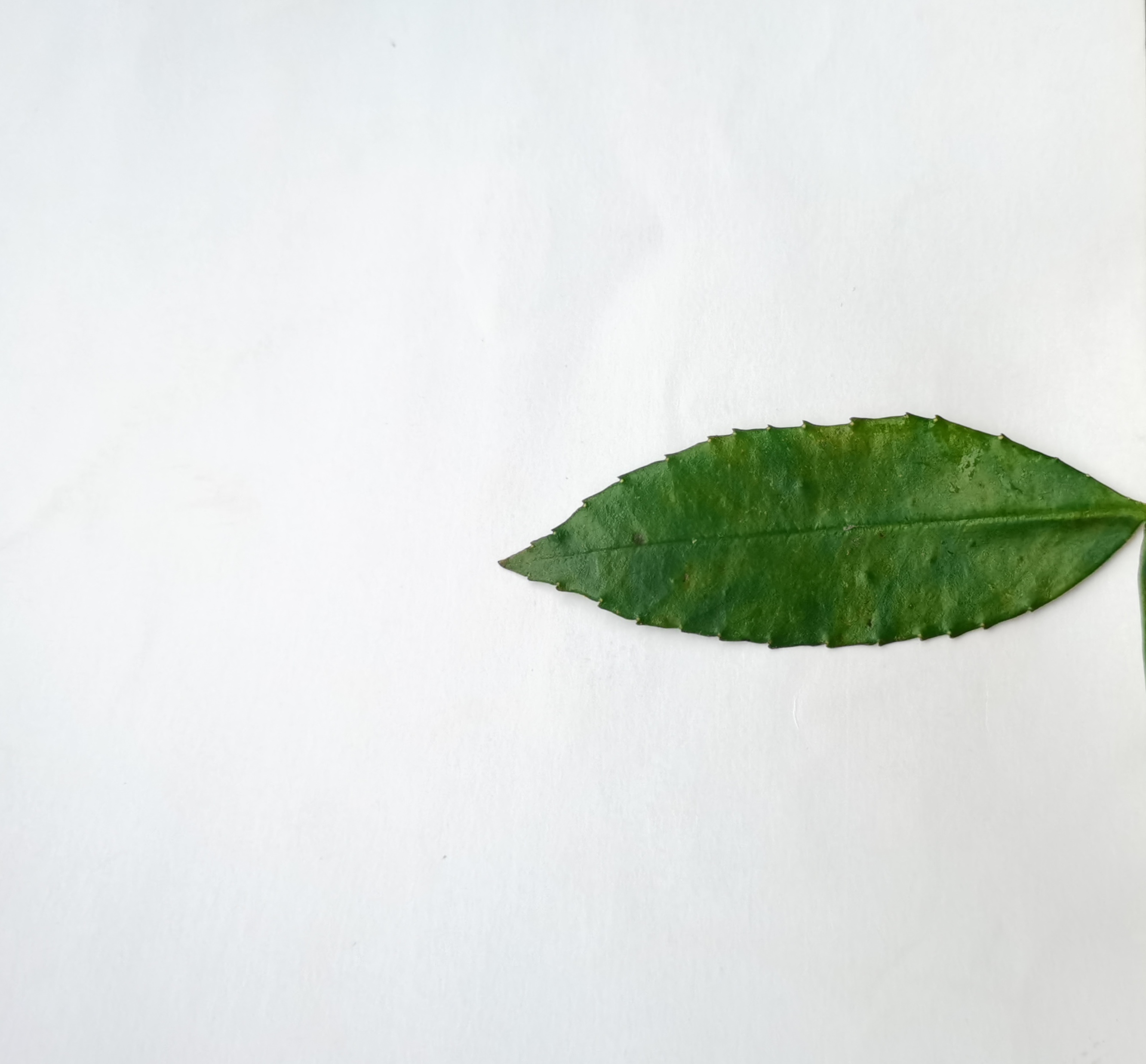

Supplement: Supplementary file 1 [file ijms-24-14761-s001.zip › Figure 1/Tengjiao-inoculated with C. zanthoxyli/IMG_20211104_175305_edit_819185152442187.jpg]

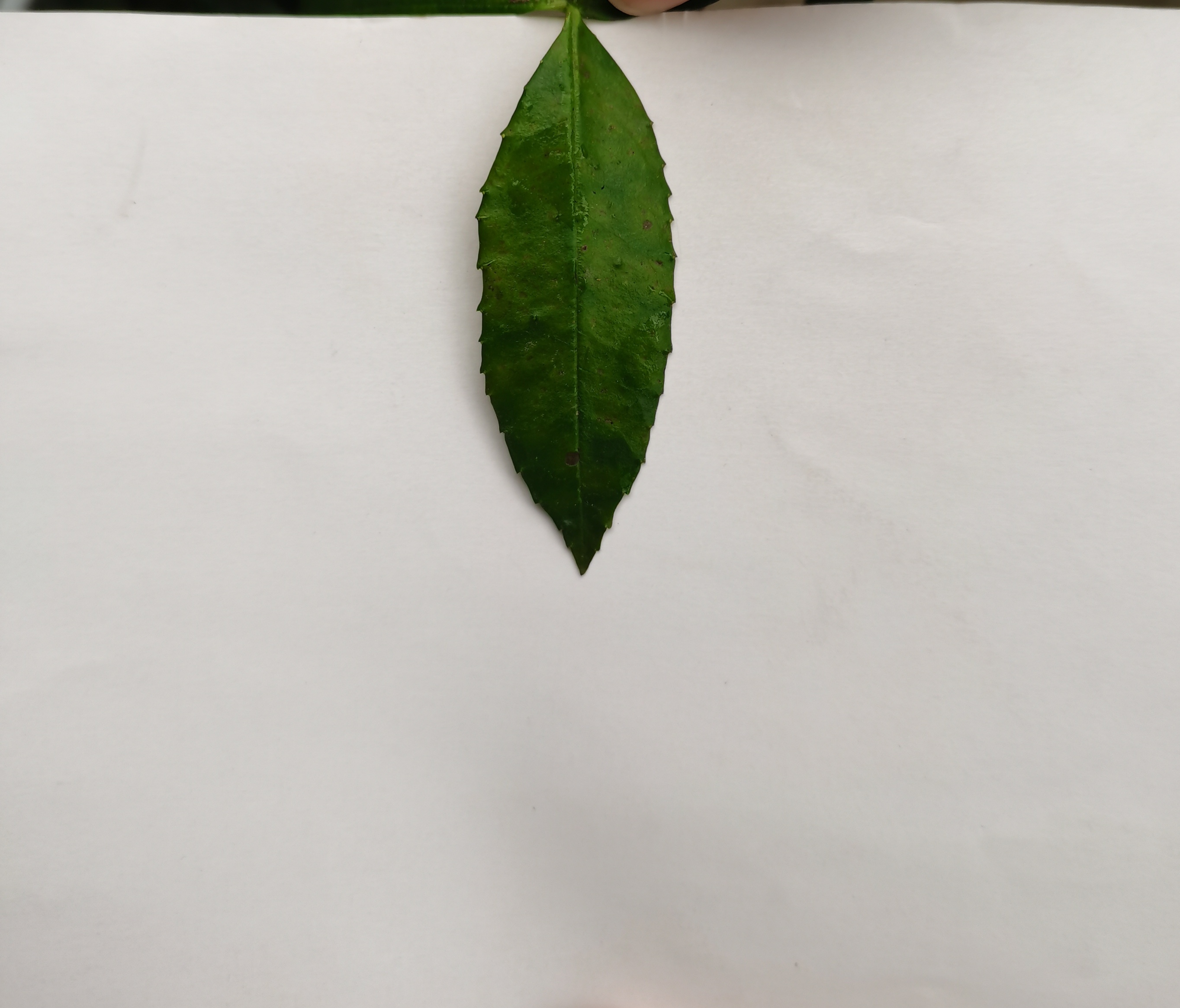

Supplement: Supplementary file 1 [file ijms-24-14761-s001.zip › Figure 1/Tengjiao-inoculated with C. zanthoxyli/IMG_20211105_163810_edit_821337003451233.jpg]

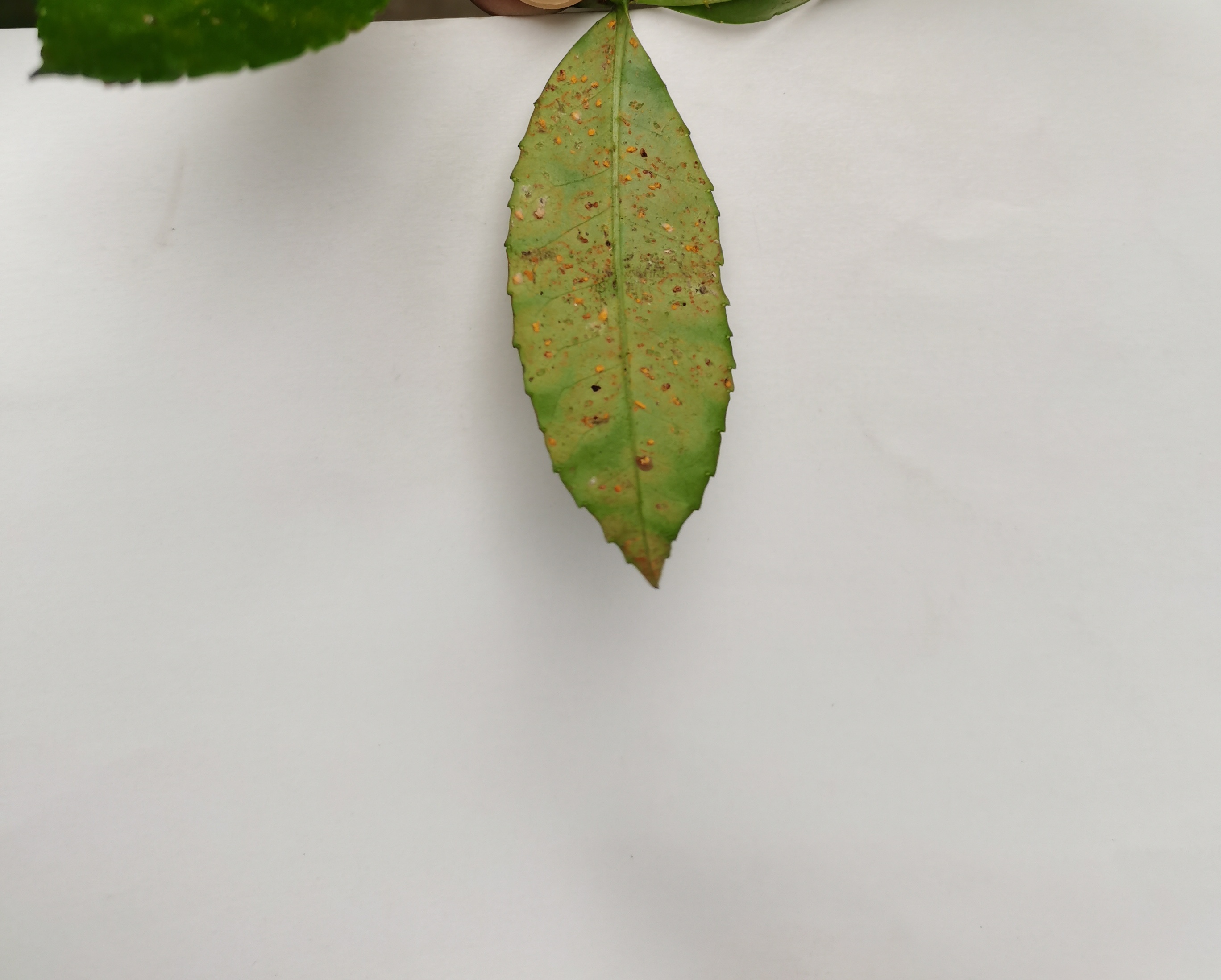

Supplement: Supplementary file 1 [file ijms-24-14761-s001.zip › Figure 1/Tengjiao-inoculated with C. zanthoxyli/IMG_20211105_163816_edit_821344682634565.jpg]

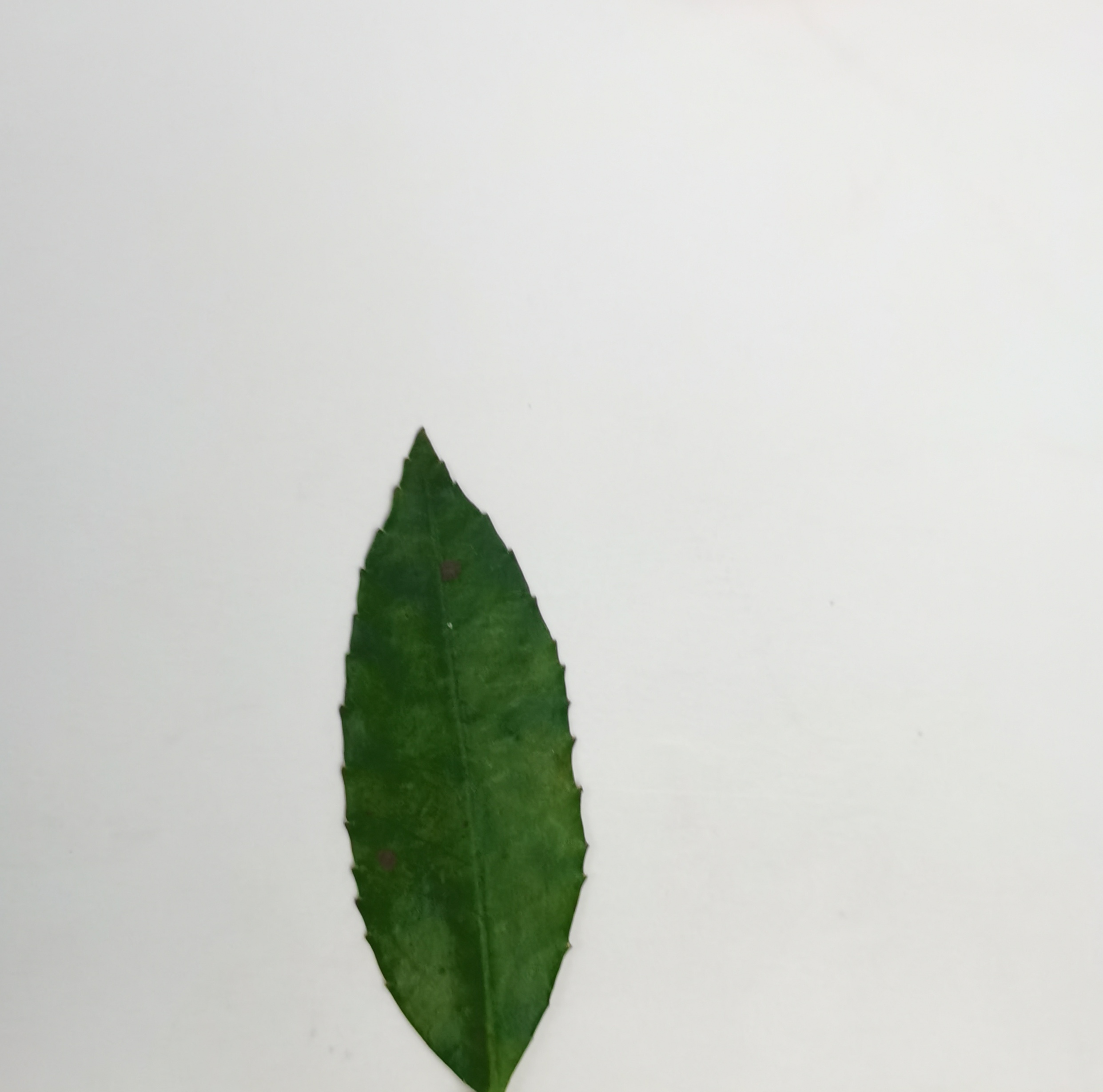

Supplement: Supplementary file 1 [file ijms-24-14761-s001.zip › Figure 1/Tengjiao-inoculated with C. zanthoxyli/IMG_20211106_173853_edit_900731629827139.jpg]

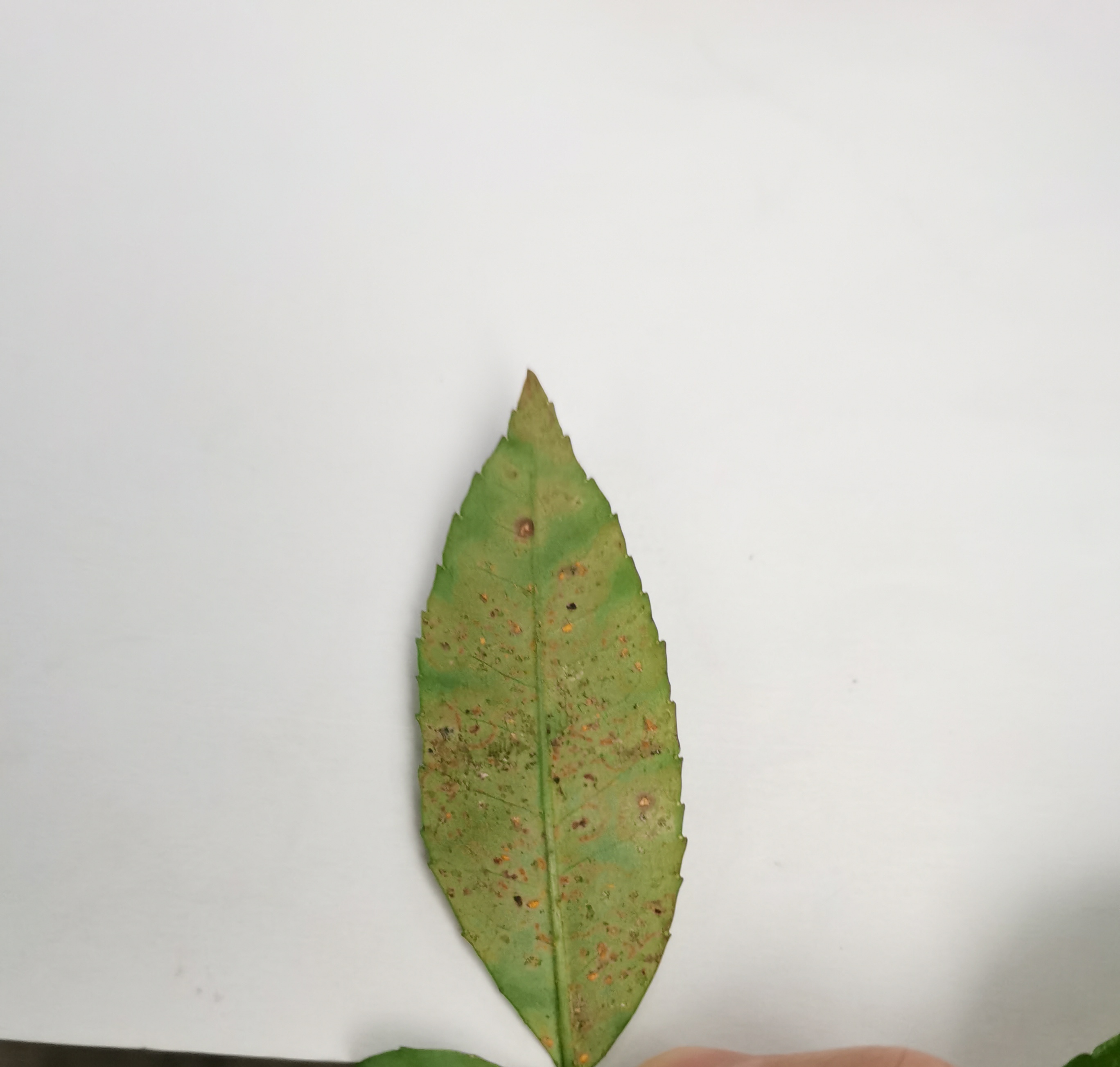

Supplement: Supplementary file 1 [file ijms-24-14761-s001.zip › Figure 1/Tengjiao-inoculated with C. zanthoxyli/IMG_20211106_173919_edit_900741510664637.jpg]

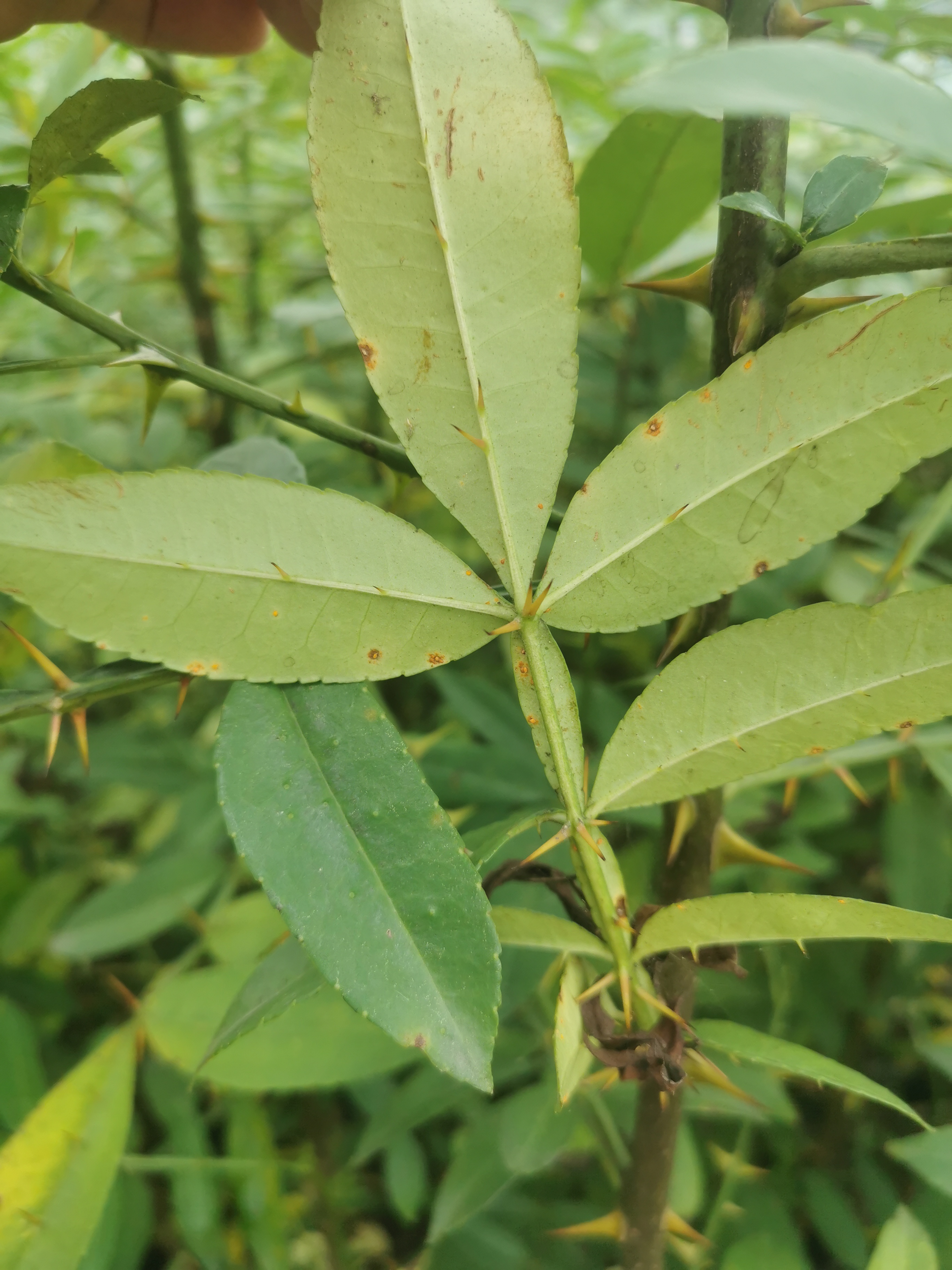

Supplement: Supplementary file 1 [file ijms-24-14761-s001.zip › Figure 1/Youkang.jpg]

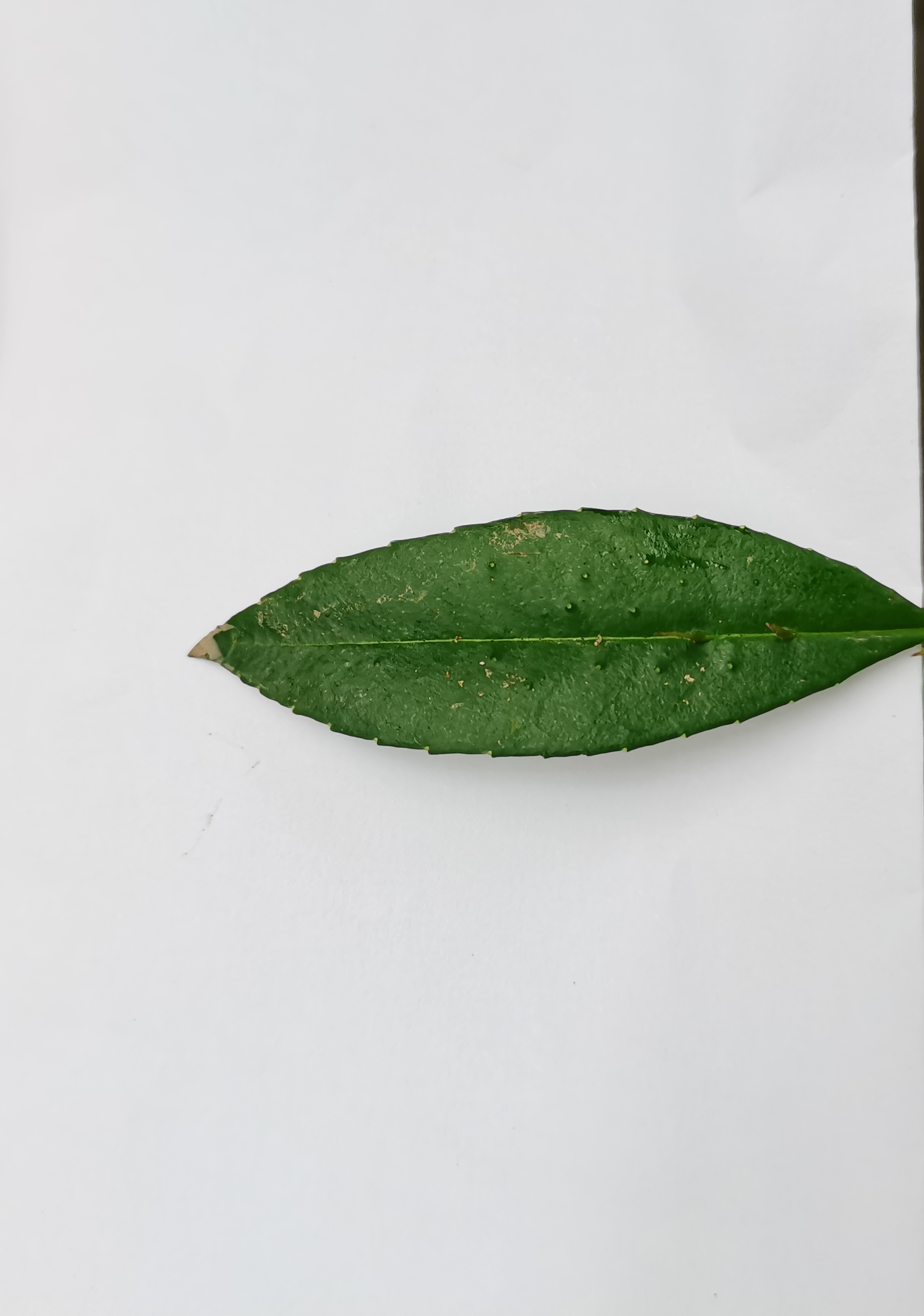

Supplement: Supplementary file 1 [file ijms-24-14761-s001.zip › Figure 1/Youkang-inoculated with C. zanthoxyli/IMG_20211115_155201_edit_157737315409263.jpg]

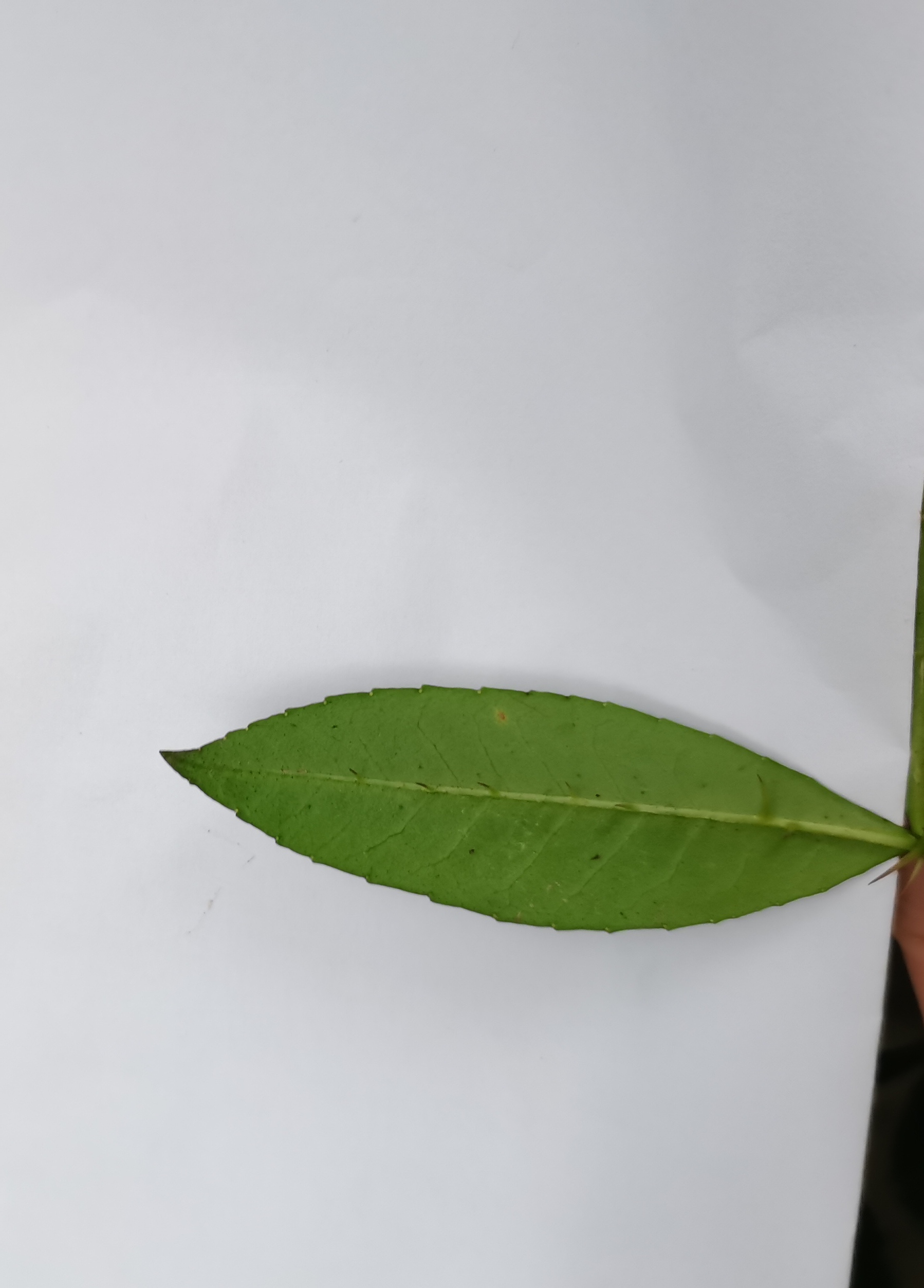

Supplement: Supplementary file 1 [file ijms-24-14761-s001.zip › Figure 1/Youkang-inoculated with C. zanthoxyli/IMG_20211115_155211_edit_157746489757699.jpg]

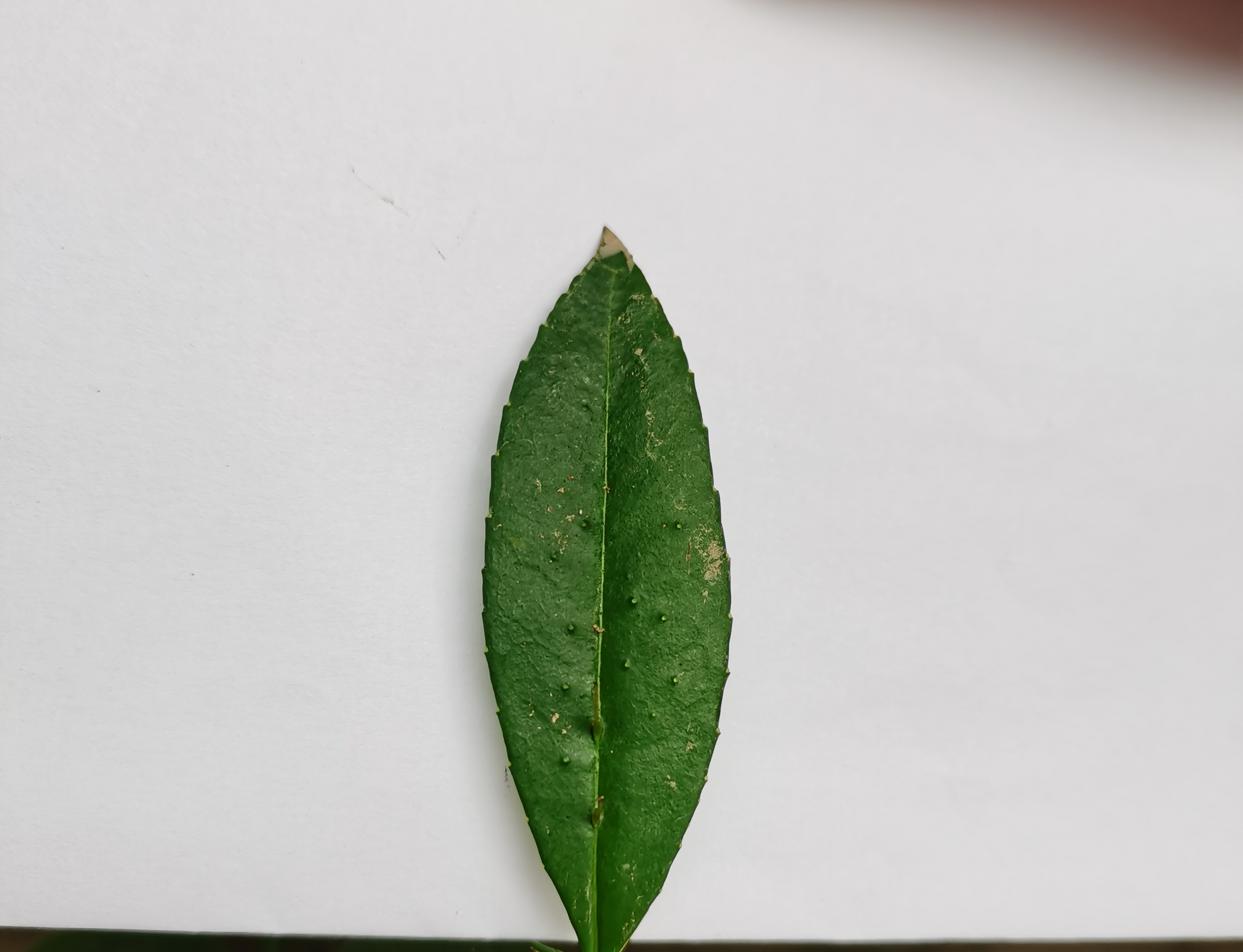

Supplement: Supplementary file 1 [file ijms-24-14761-s001.zip › Figure 1/Youkang-inoculated with C. zanthoxyli/IMG_20211116_150143_edit_184799168470238.jpg]

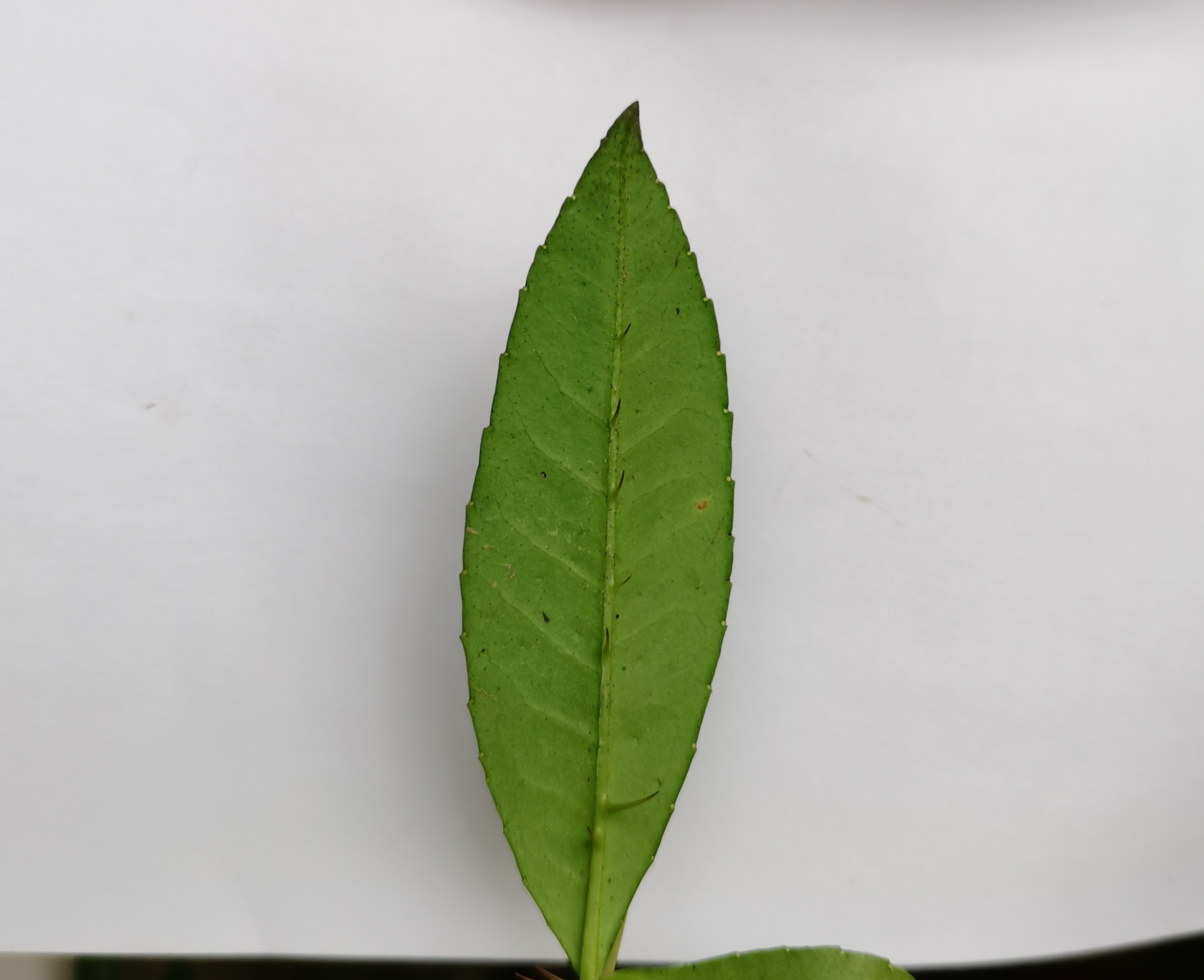

Supplement: Supplementary file 1 [file ijms-24-14761-s001.zip › Figure 1/Youkang-inoculated with C. zanthoxyli/IMG_20211116_150208_edit_184810239048361.jpg]

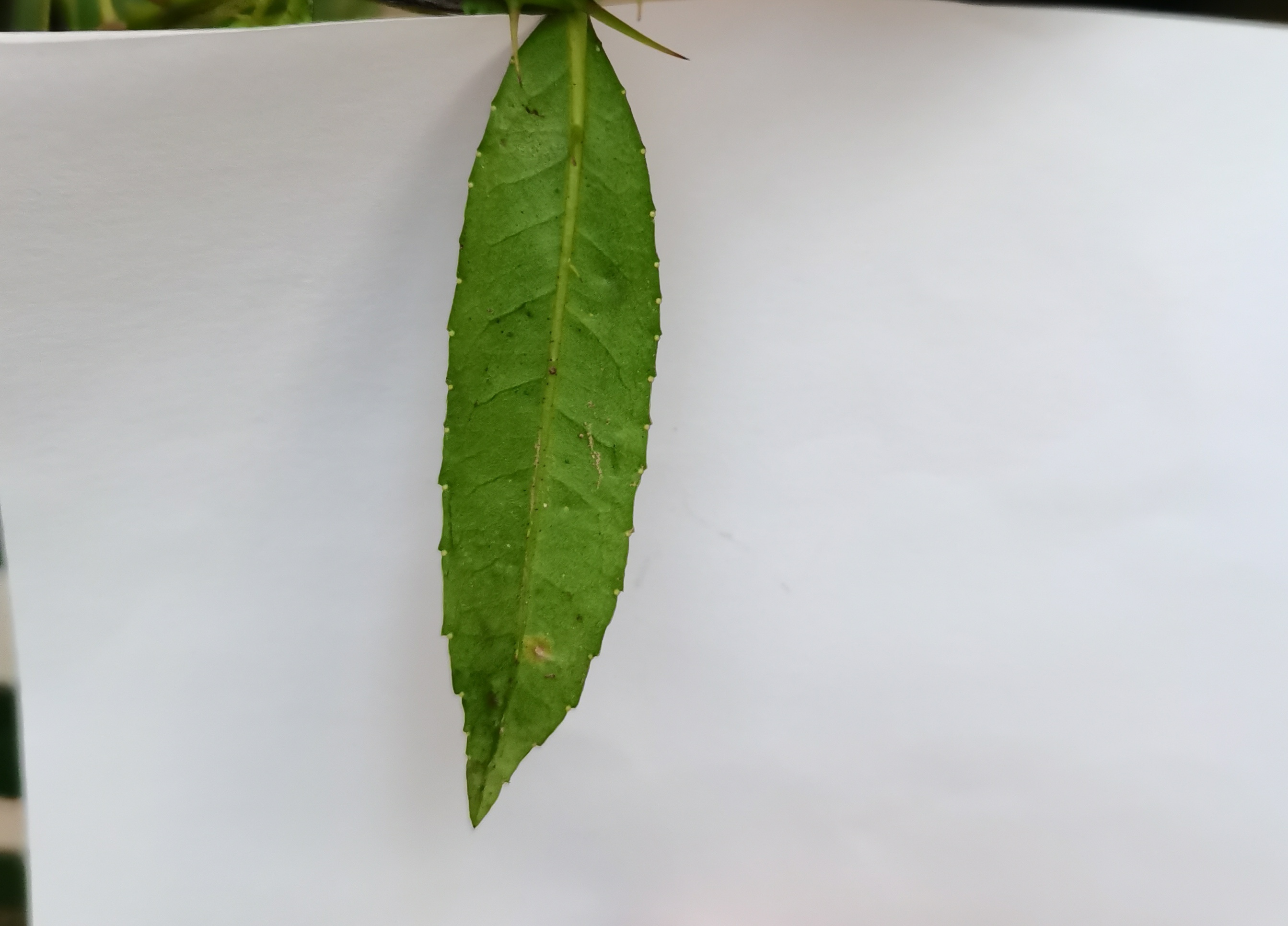

Supplement: Supplementary file 1 [file ijms-24-14761-s001.zip › Figure 1/Youkang-inoculated with C. zanthoxyli/IMG_20211117_173553_edit_227043644301813.jpg]

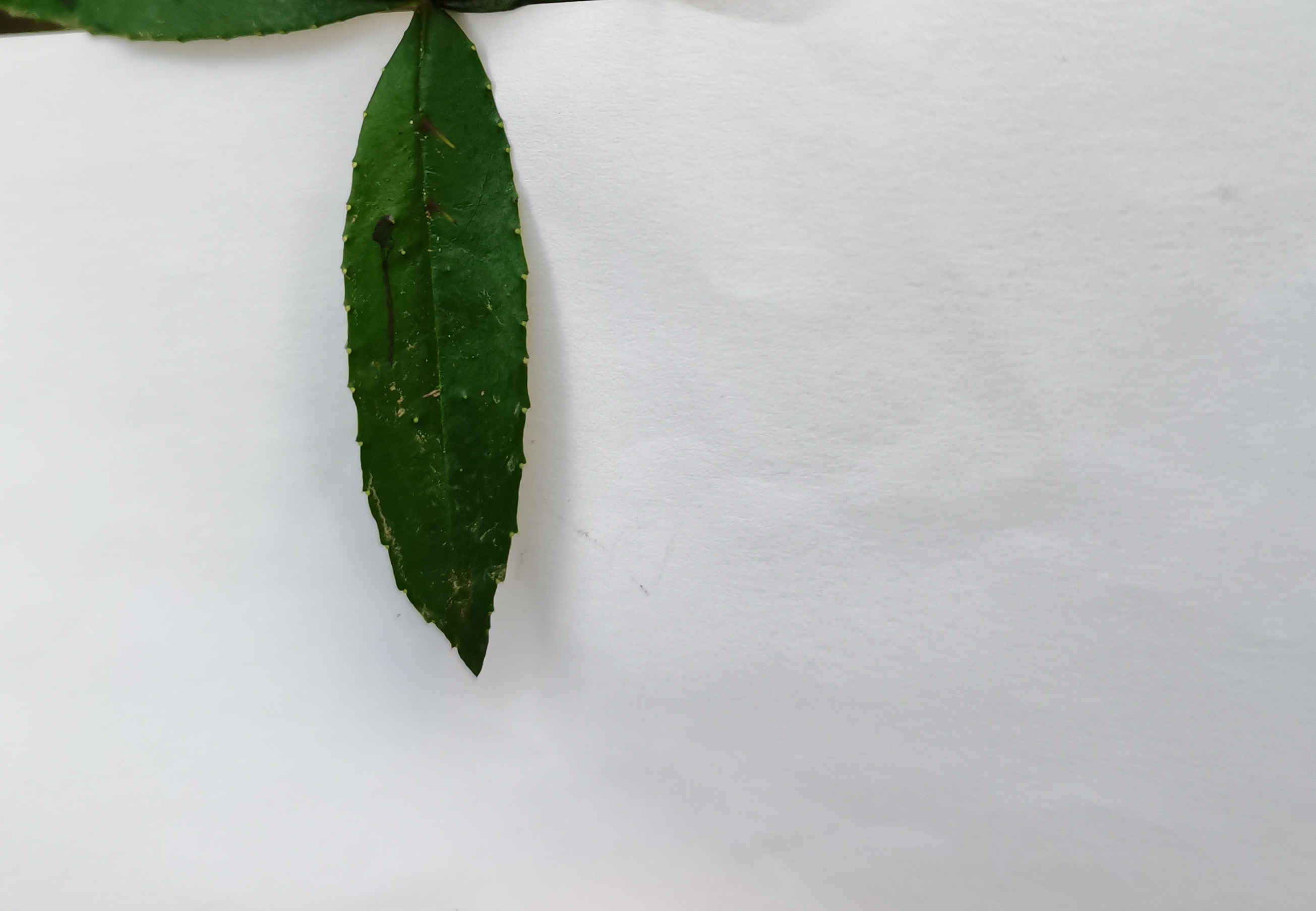

Supplement: Supplementary file 1 [file ijms-24-14761-s001.zip › Figure 1/Youkang-inoculated with C. zanthoxyli/IMG_20211117_173607_edit_227053408687228.jpg]

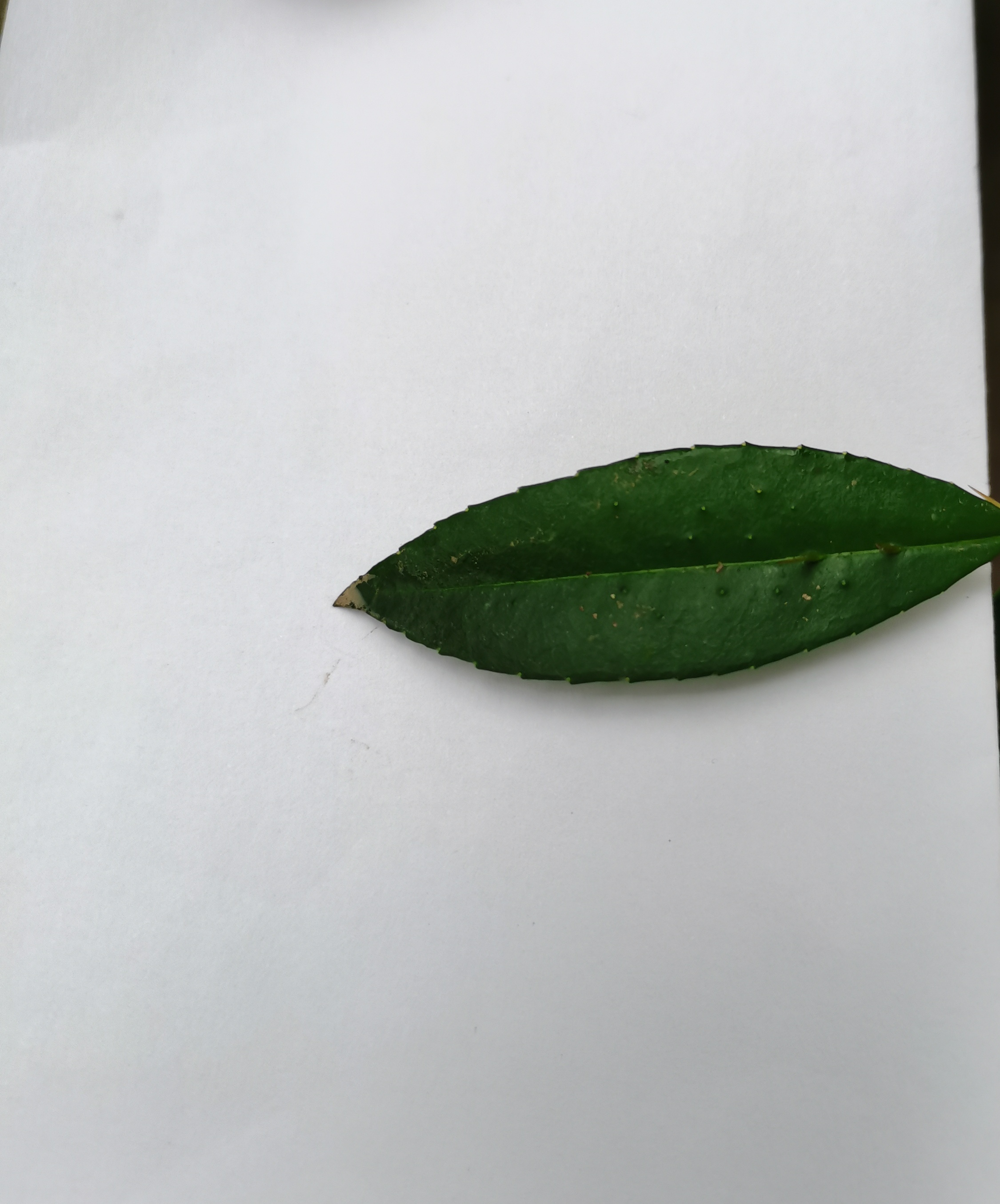

Supplement: Supplementary file 1 [file ijms-24-14761-s001.zip › Figure 1/Youkang-inoculated with C. zanthoxyli/IMG_20211118_163044_edit_254251699803390.jpg]

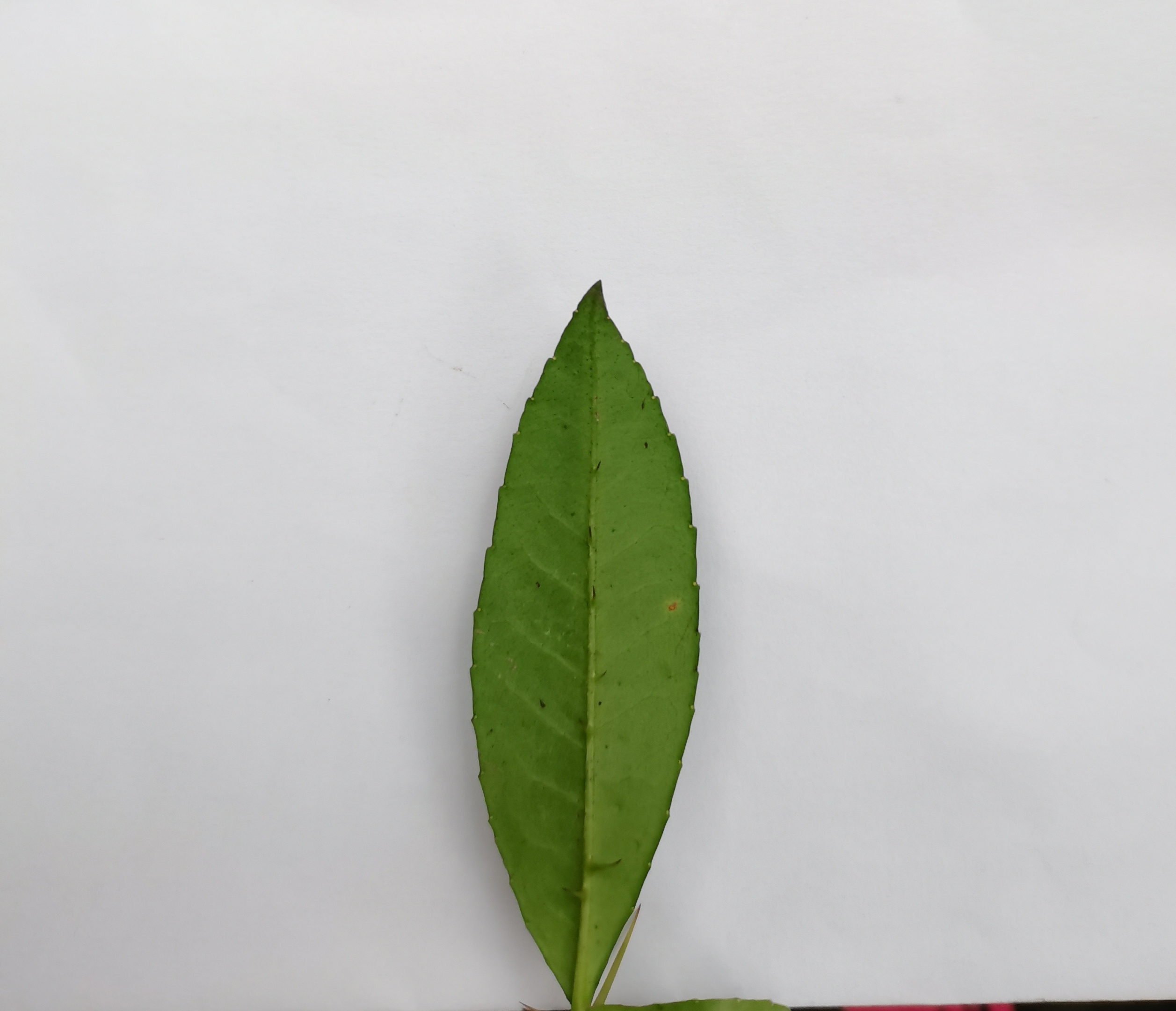

Supplement: Supplementary file 1 [file ijms-24-14761-s001.zip › Figure 1/Youkang-inoculated with C. zanthoxyli/IMG_20211118_163106_edit_254263061567451.jpg]

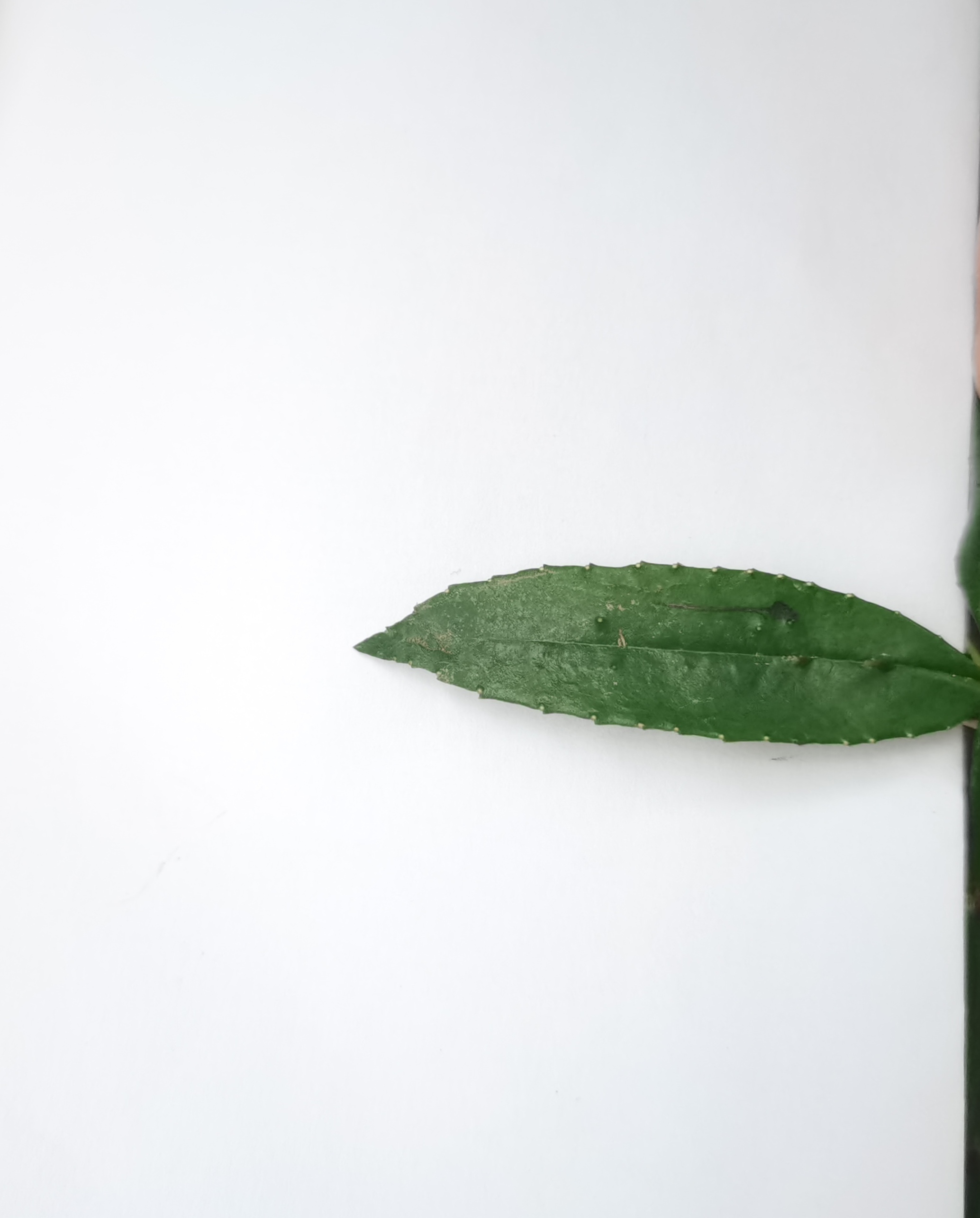

Supplement: Supplementary file 1 [file ijms-24-14761-s001.zip › Figure 1/Youkang-inoculated with C. zanthoxyli/IMG_20211119_173104_edit_306895894818274.jpg]

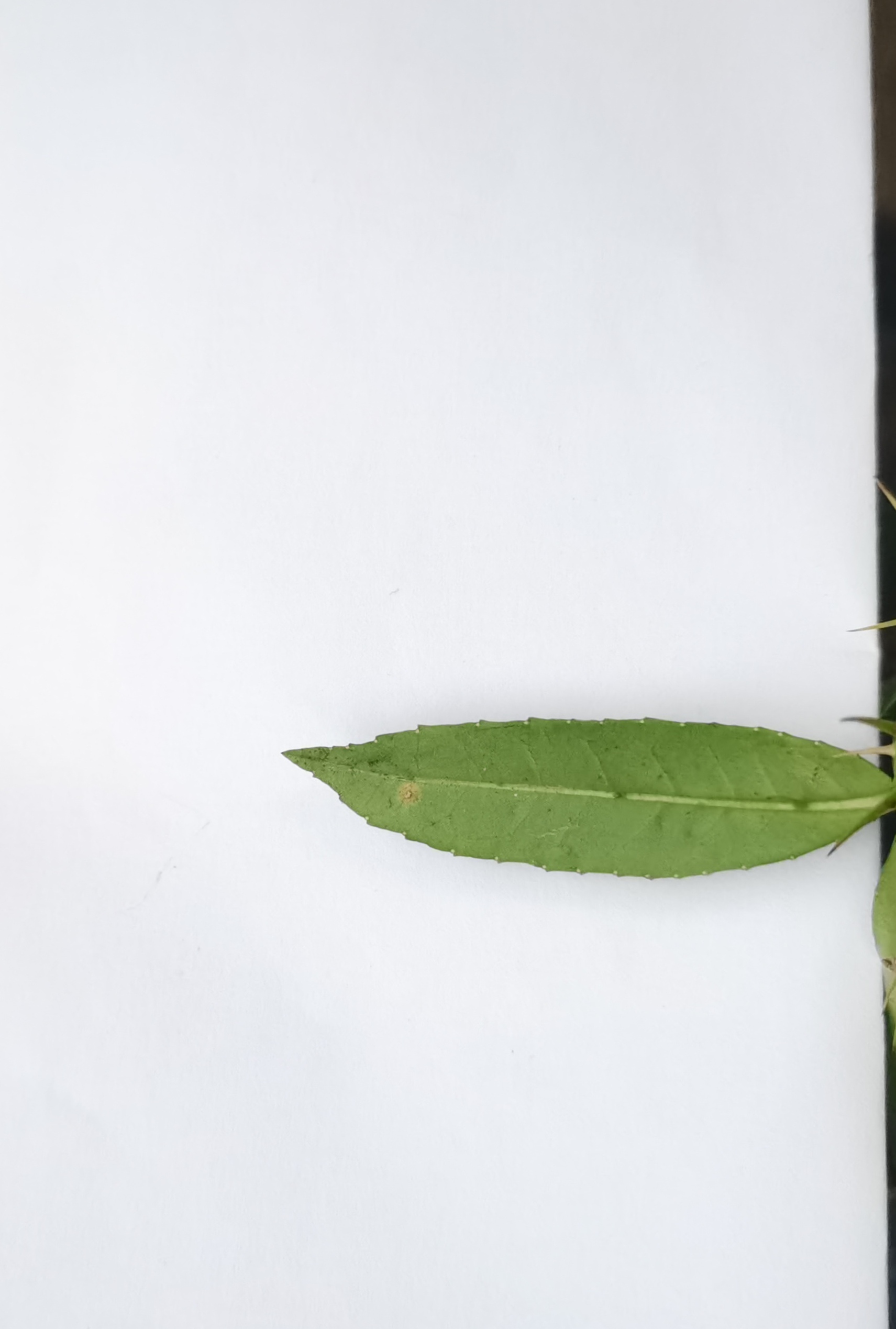

Supplement: Supplementary file 1 [file ijms-24-14761-s001.zip › Figure 1/Youkang-inoculated with C. zanthoxyli/IMG_20211119_173113_edit_306906685654210.jpg]

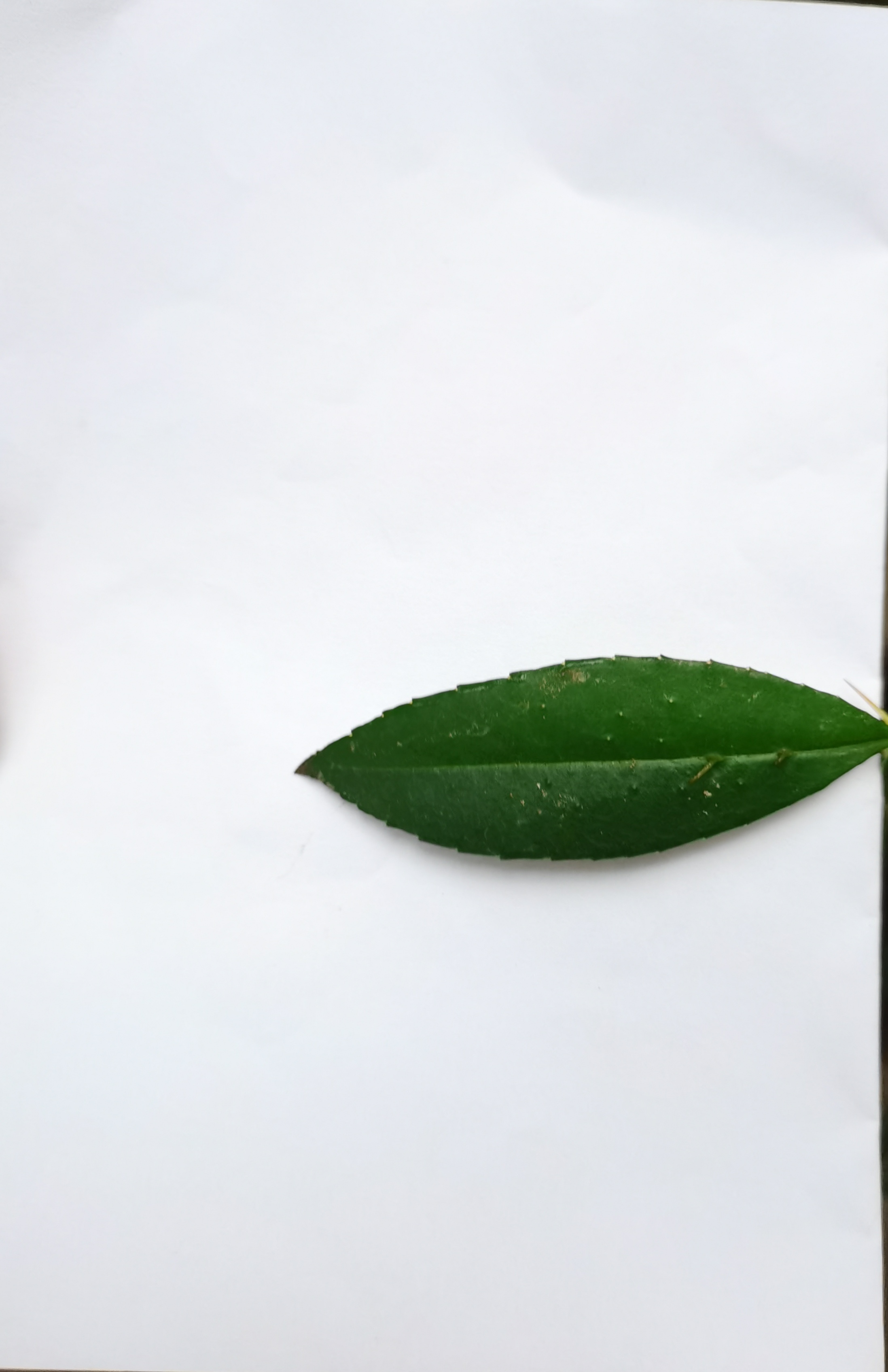

Supplement: Supplementary file 1 [file ijms-24-14761-s001.zip › Figure 1/Youkang-inoculated with C. zanthoxyli/IMG_20211120_173613_edit_337280505359992.jpg]

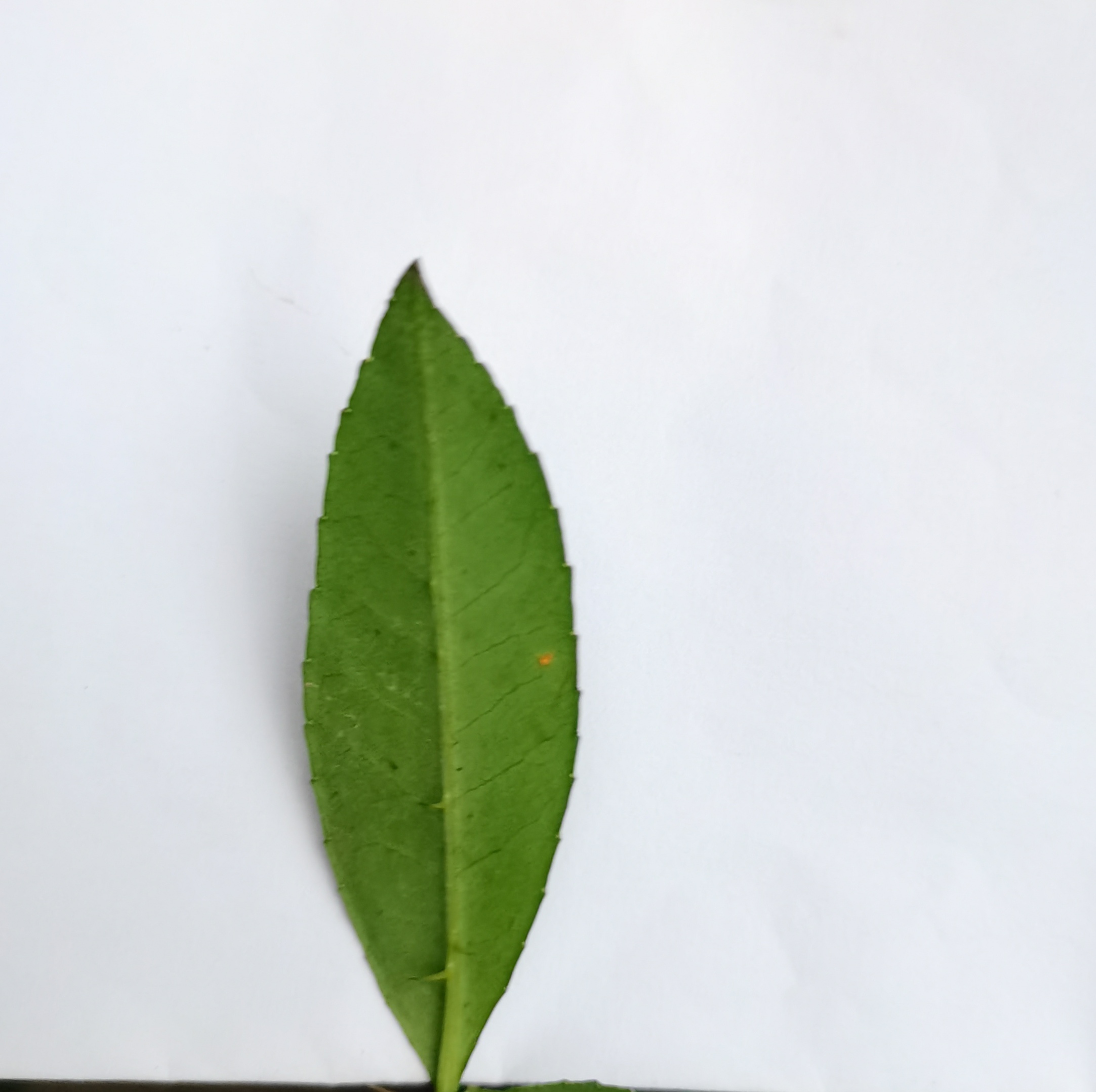

Supplement: Supplementary file 1 [file ijms-24-14761-s001.zip › Figure 1/Youkang-inoculated with C. zanthoxyli/IMG_20211120_173621_edit_337290689102699.jpg]

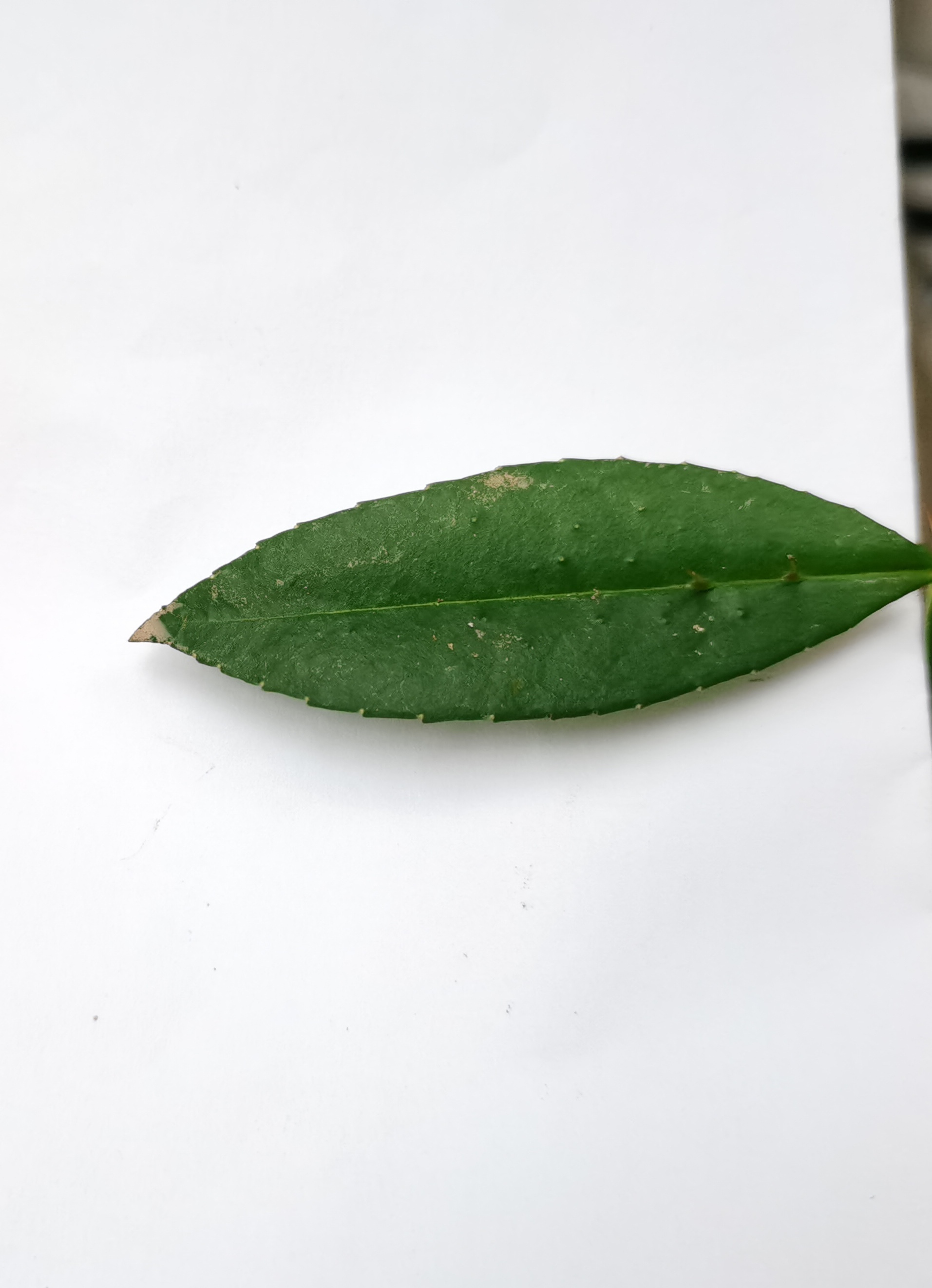

Supplement: Supplementary file 1 [file ijms-24-14761-s001.zip › Figure 1/Youkang-inoculated with C. zanthoxyli/IMG_20211121_171543_edit_394359523099199.jpg]

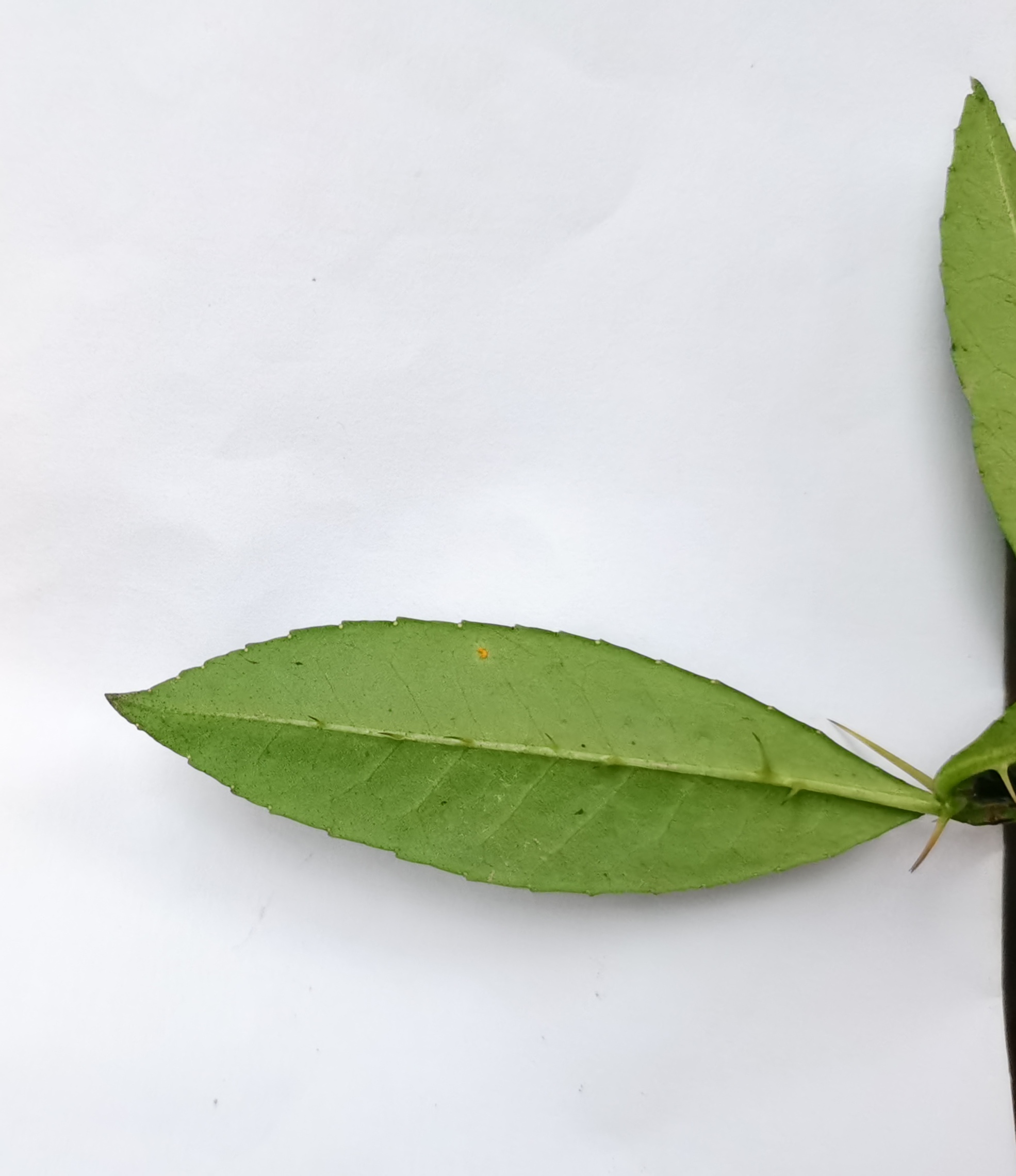

Supplement: Supplementary file 1 [file ijms-24-14761-s001.zip › Figure 1/Youkang-inoculated with C. zanthoxyli/IMG_20211121_171559_edit_394373877673676.jpg]

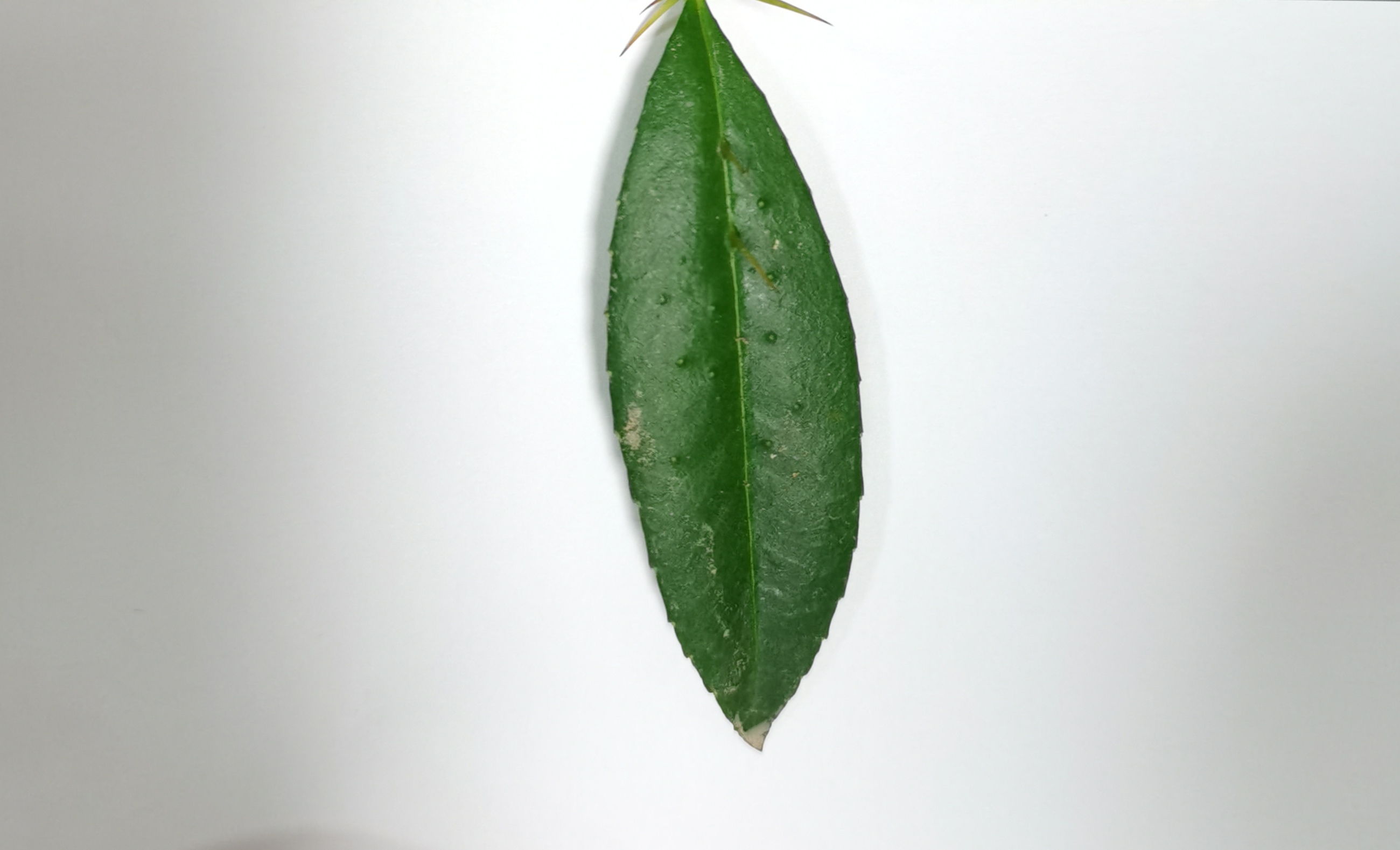

Supplement: Supplementary file 1 [file ijms-24-14761-s001.zip › Figure 1/Youkang-inoculated with C. zanthoxyli/IMG_20211122_192756_edit_432952502066748.jpg]

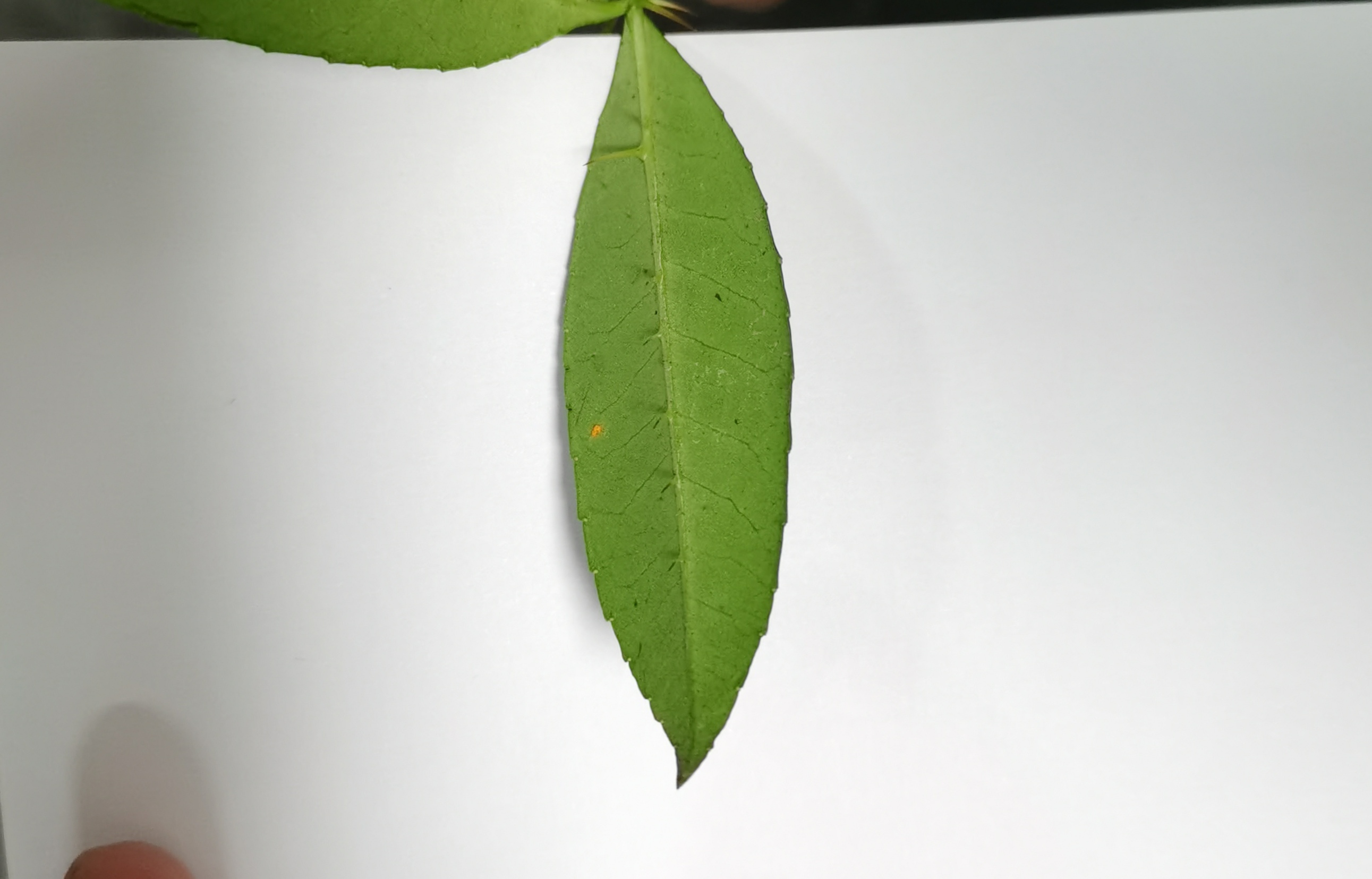

Supplement: Supplementary file 1 [file ijms-24-14761-s001.zip › Figure 1/Youkang-inoculated with C. zanthoxyli/IMG_20211122_192807_edit_432971014246432.jpg]

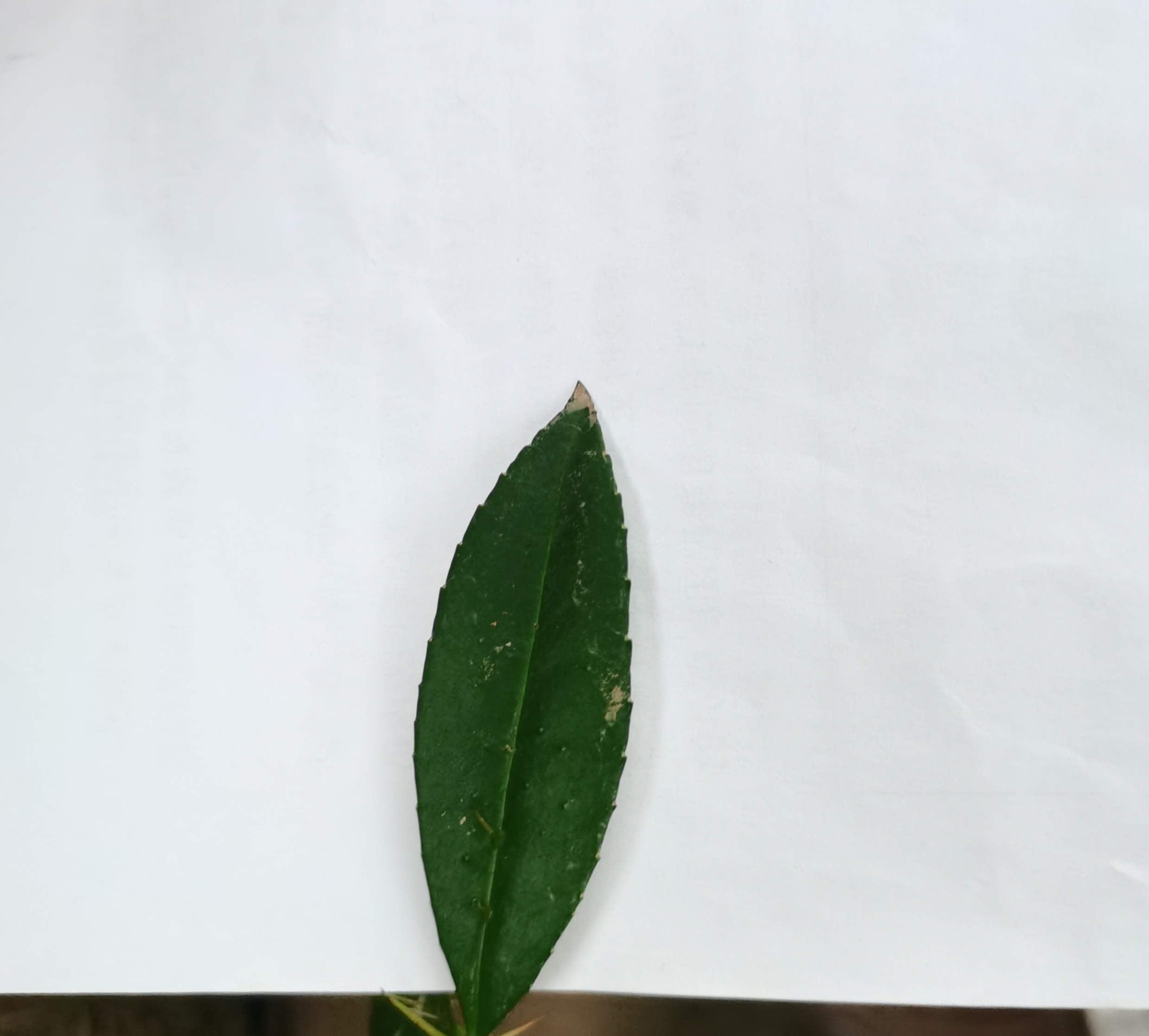

Supplement: Supplementary file 1 [file ijms-24-14761-s001.zip › Figure 1/Youkang-inoculated with C. zanthoxyli/IMG_20211123_175143_edit_457996046624385.jpg]

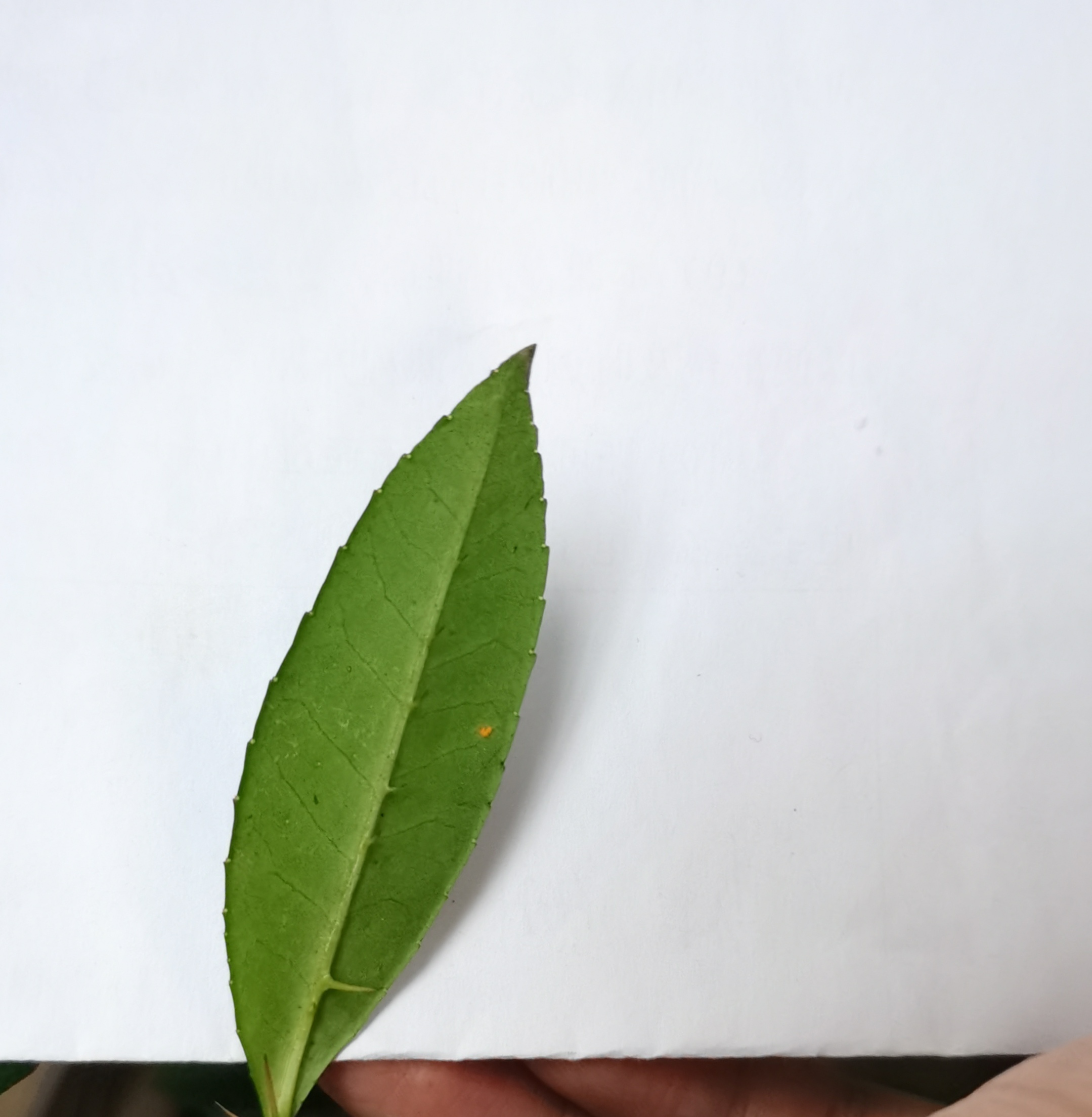

Supplement: Supplementary file 1 [file ijms-24-14761-s001.zip › Figure 1/Youkang-inoculated with C. zanthoxyli/IMG_20211123_175218_edit_458007393380112.jpg]

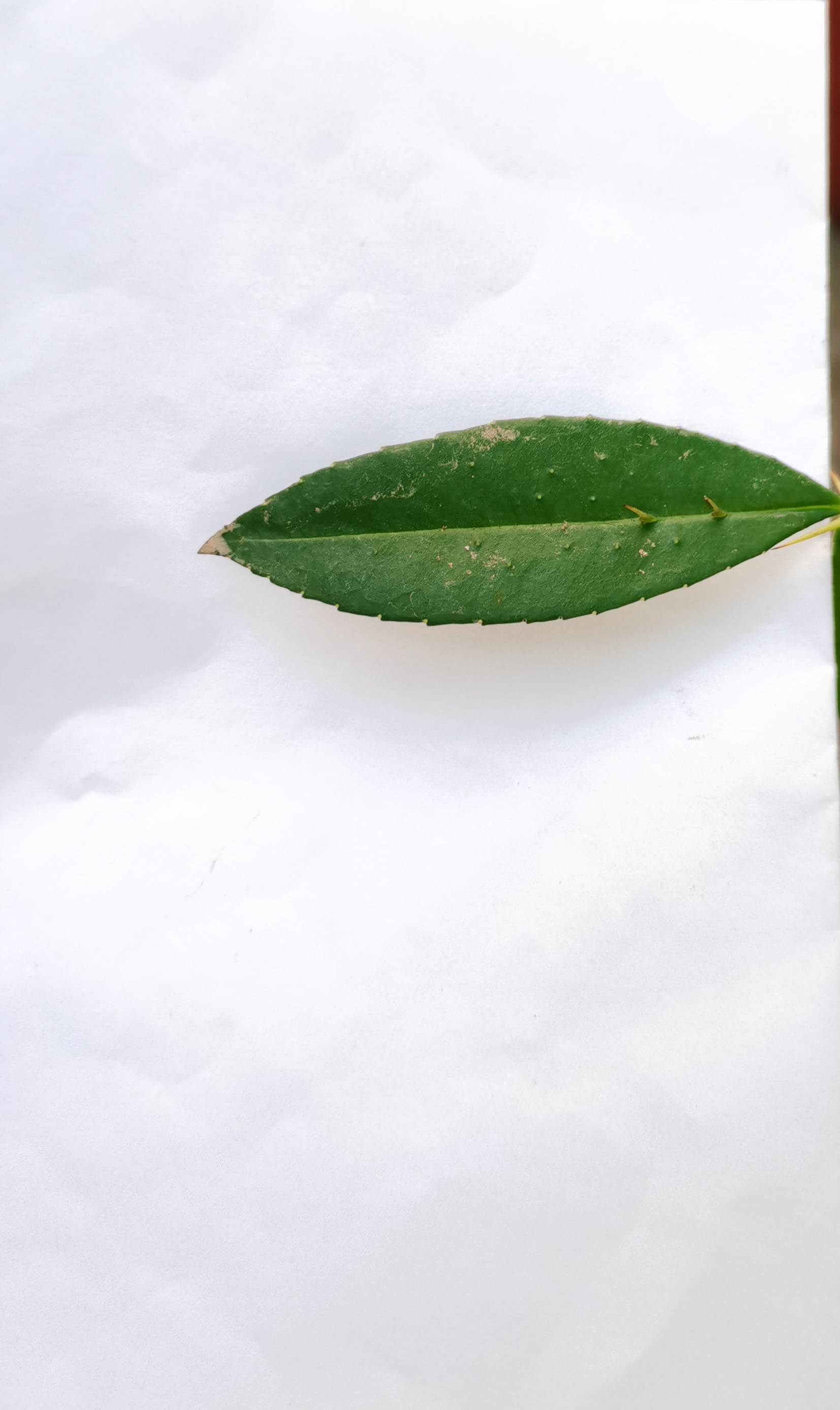

Supplement: Supplementary file 1 [file ijms-24-14761-s001.zip › Figure 1/Youkang-inoculated with C. zanthoxyli/IMG_20211124_171738_edit_509841756614910.jpg]

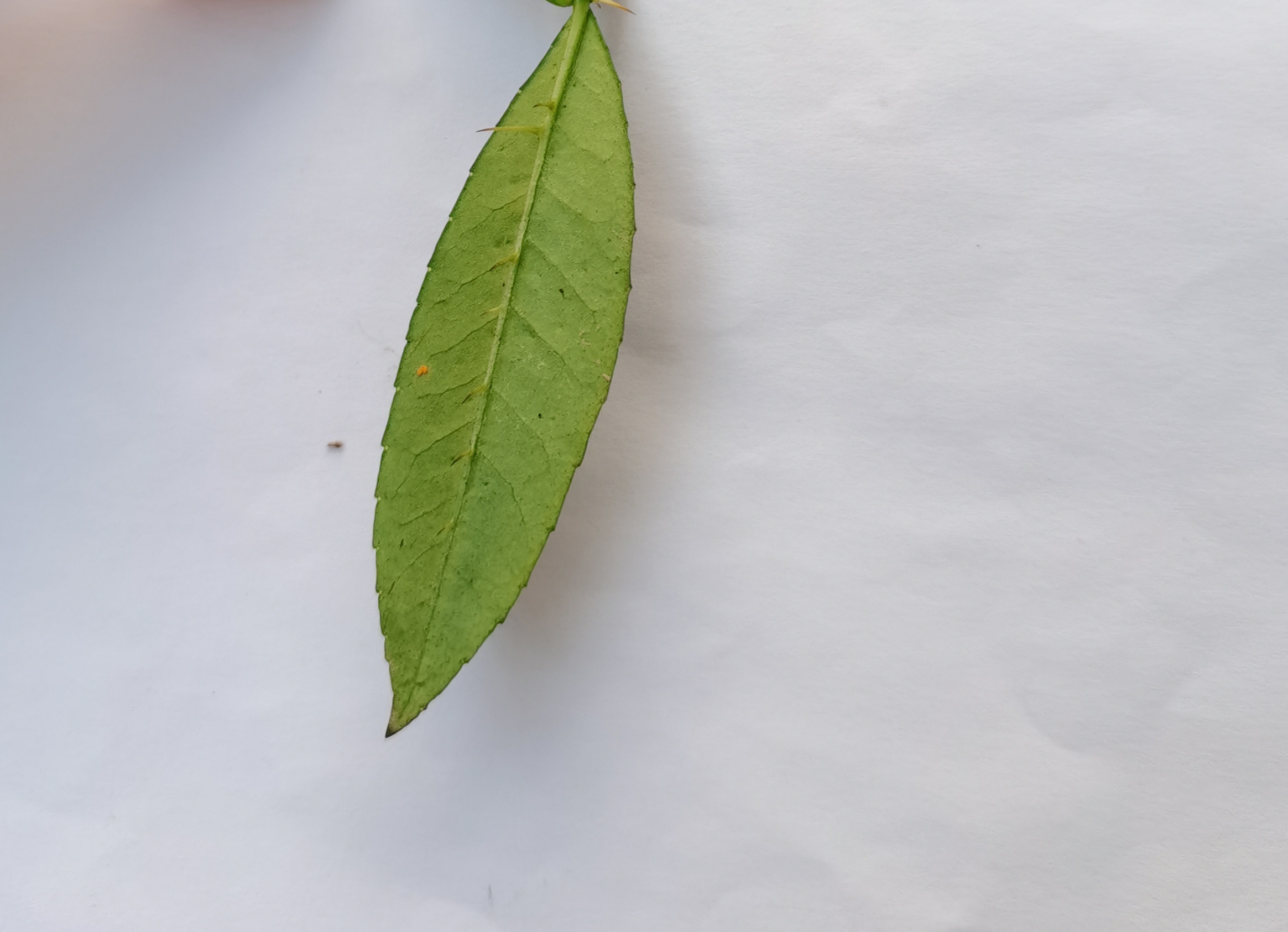

Supplement: Supplementary file 1 [file ijms-24-14761-s001.zip › Figure 1/Youkang-inoculated with C. zanthoxyli/IMG_20211124_171859_edit_509856514636783.jpg]

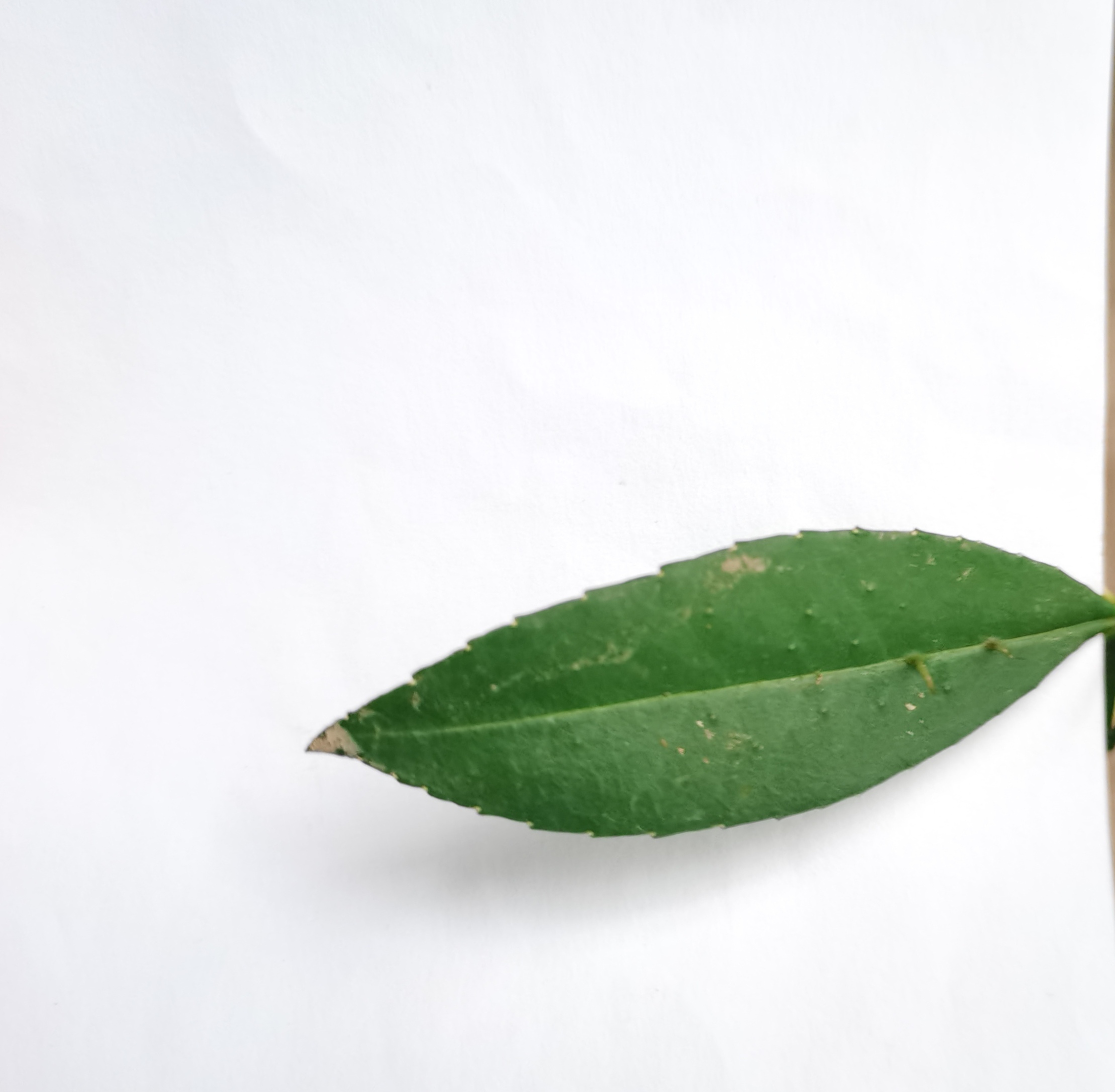

Supplement: Supplementary file 1 [file ijms-24-14761-s001.zip › Figure 1/Youkang-inoculated with C. zanthoxyli/IMG_20211125_170930_edit_535918939000515.jpg]

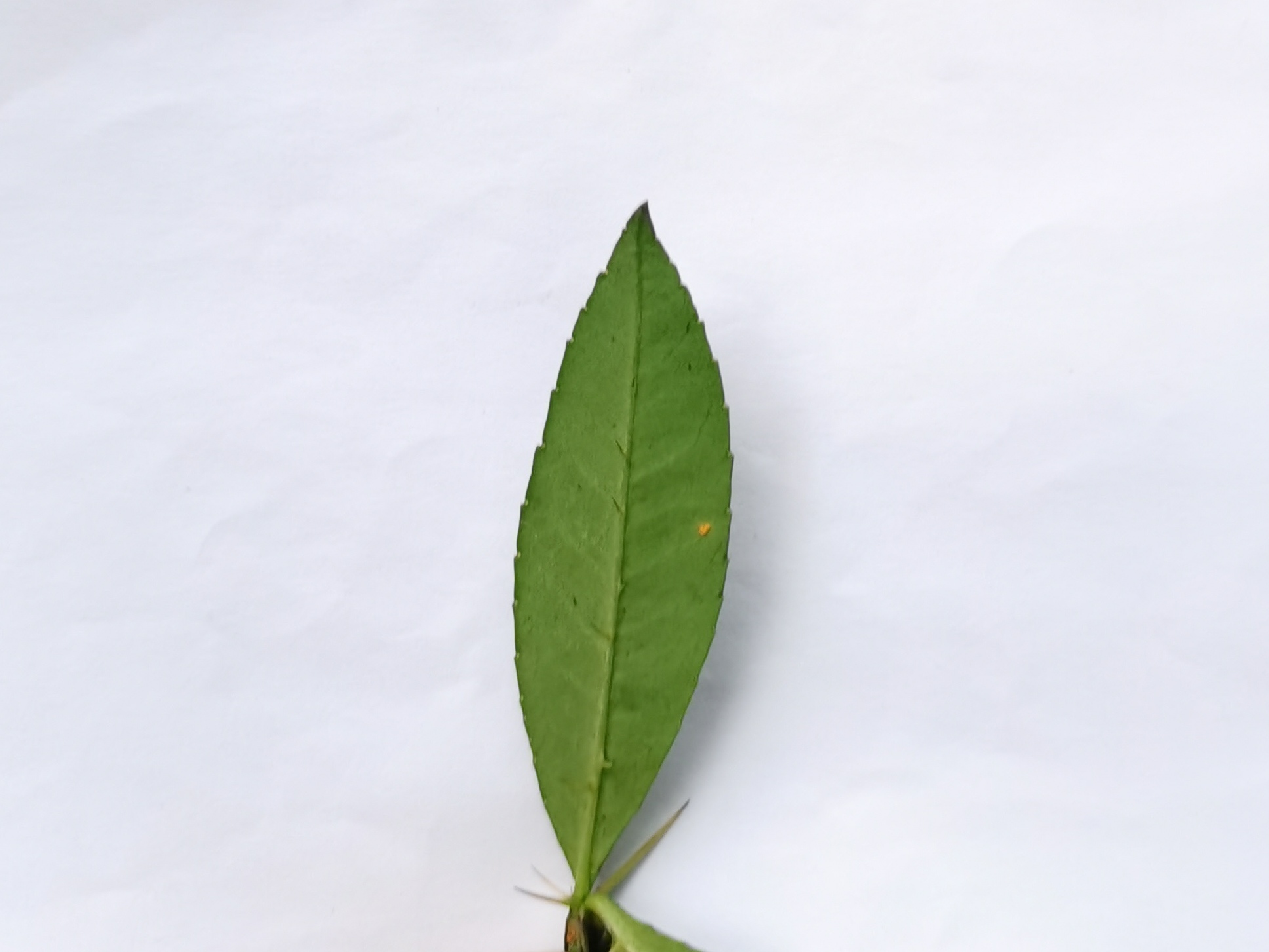

Supplement: Supplementary file 1 [file ijms-24-14761-s001.zip › Figure 1/Youkang-inoculated with C. zanthoxyli/IMG_20211125_171033_edit_535930942139054.jpg]

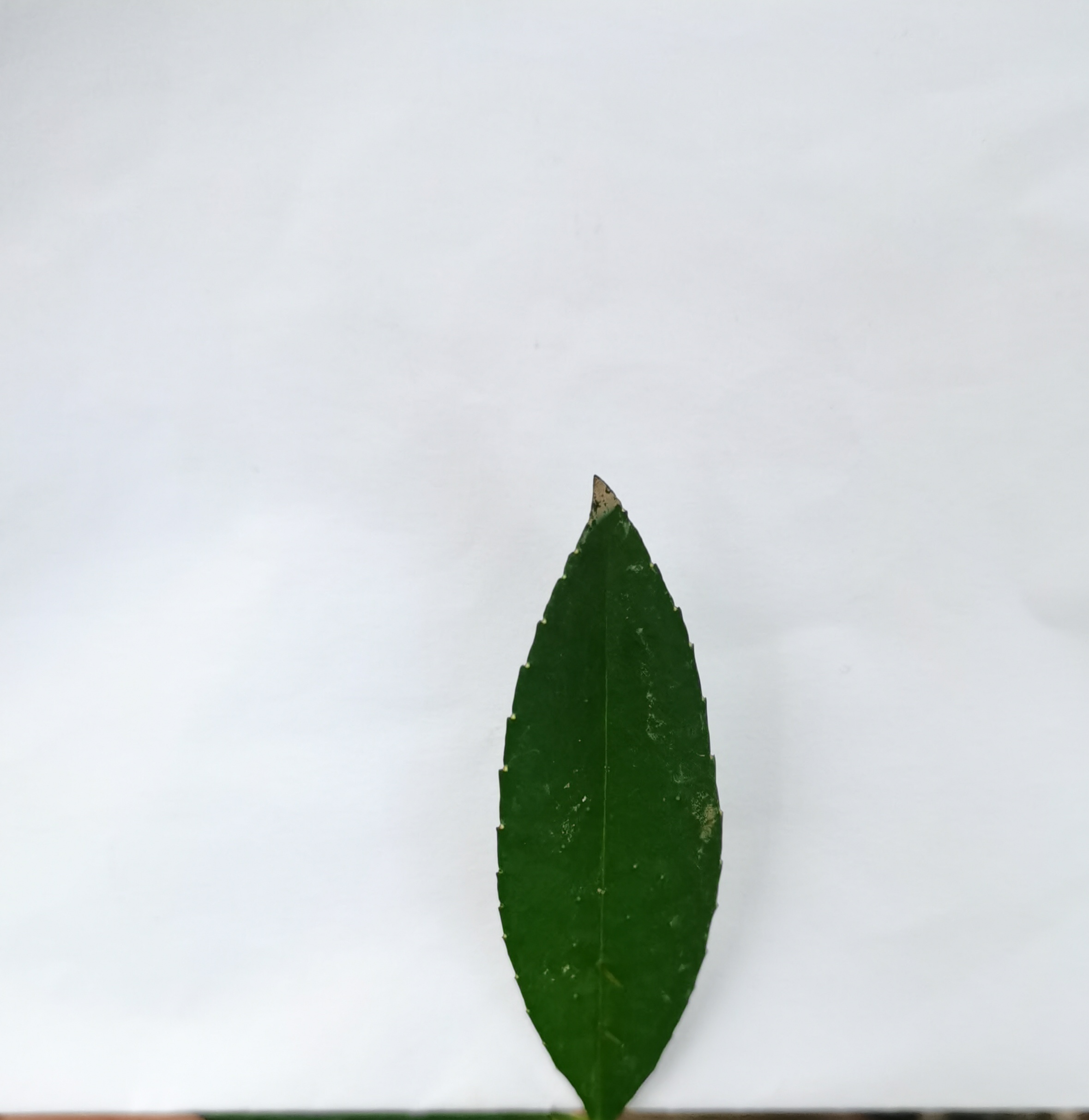

Supplement: Supplementary file 1 [file ijms-24-14761-s001.zip › Figure 1/Youkang-inoculated with C. zanthoxyli/IMG_20211126_173305_edit_571725120321093.jpg]

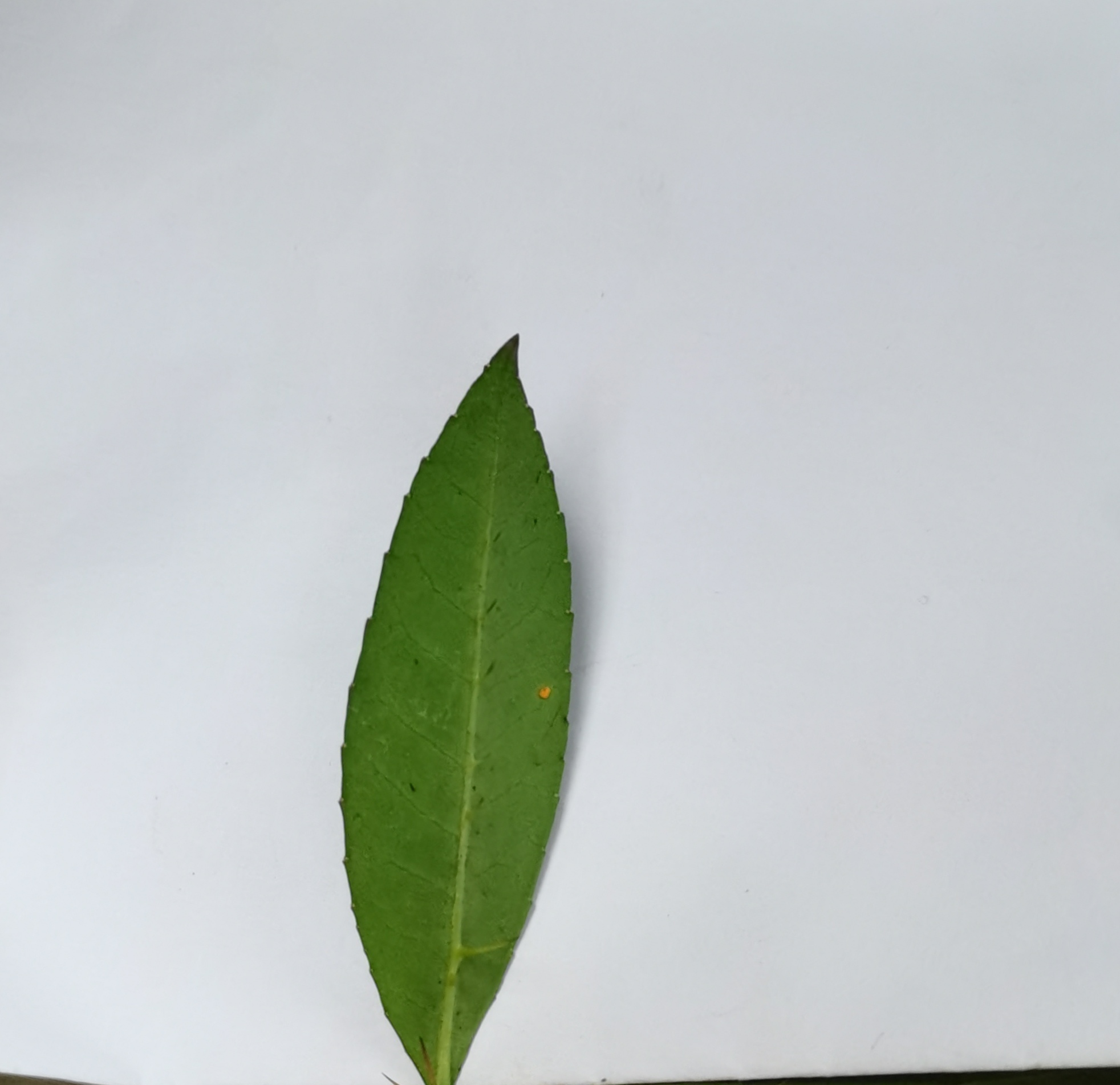

Supplement: Supplementary file 1 [file ijms-24-14761-s001.zip › Figure 1/Youkang-inoculated with C. zanthoxyli/IMG_20211126_173311_edit_571738368155986.jpg]

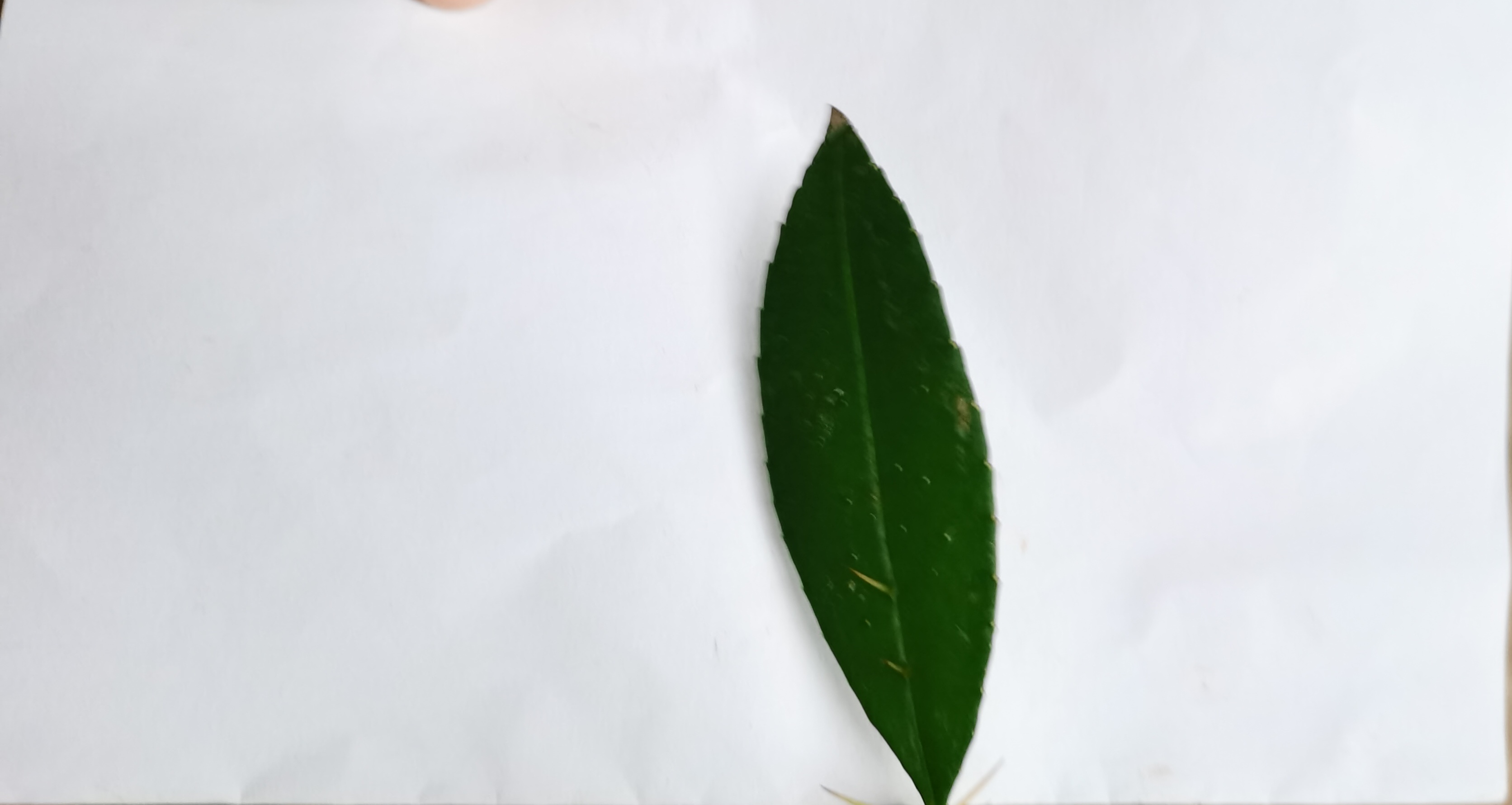

Supplement: Supplementary file 1 [file ijms-24-14761-s001.zip › Figure 1/Youkang-inoculated with C. zanthoxyli/IMG_20211127_172339_edit_586208192292841.jpg]

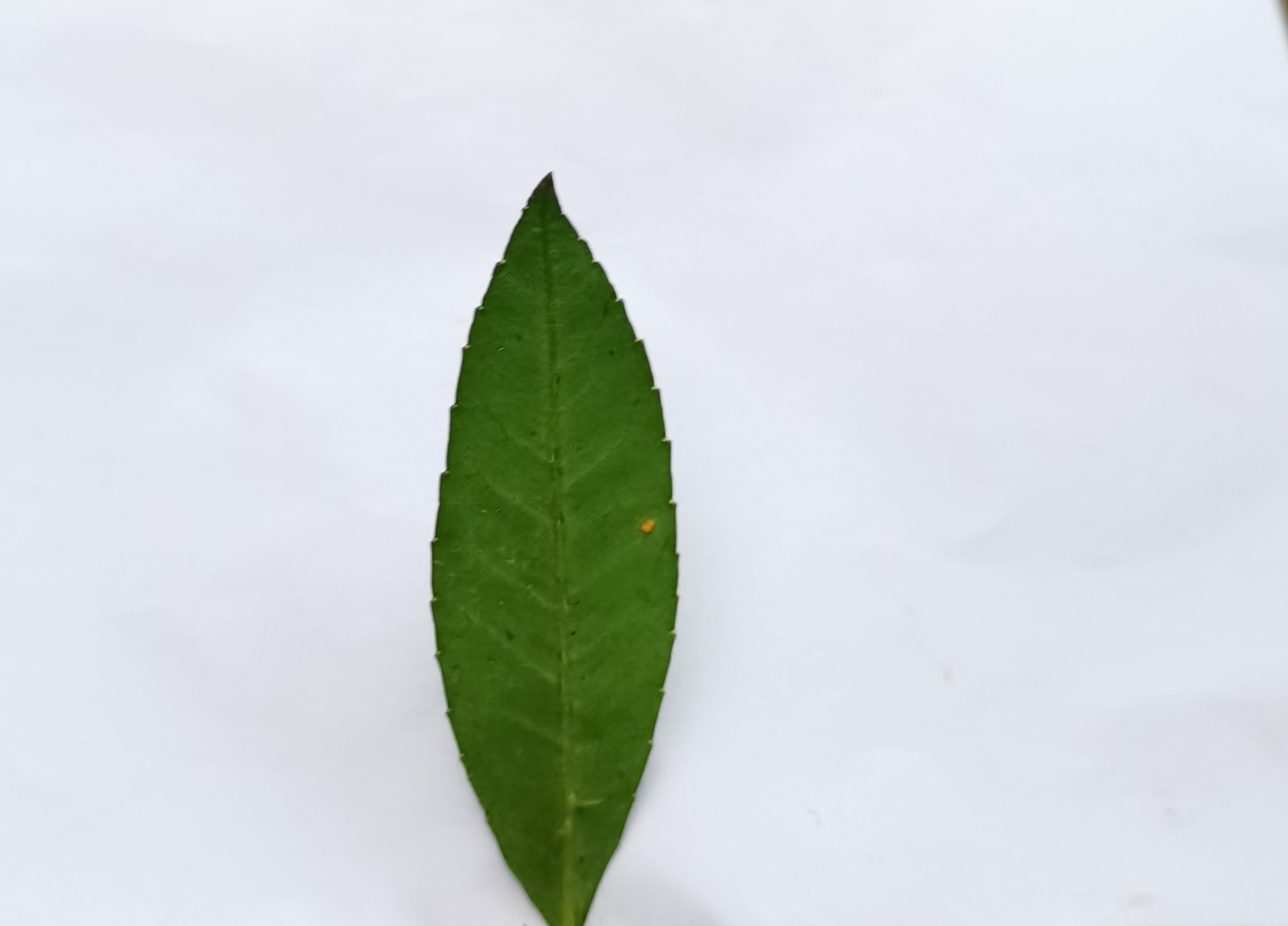

Supplement: Supplementary file 1 [file ijms-24-14761-s001.zip › Figure 1/Youkang-inoculated with C. zanthoxyli/IMG_20211127_172401_edit_586218137601173.jpg]

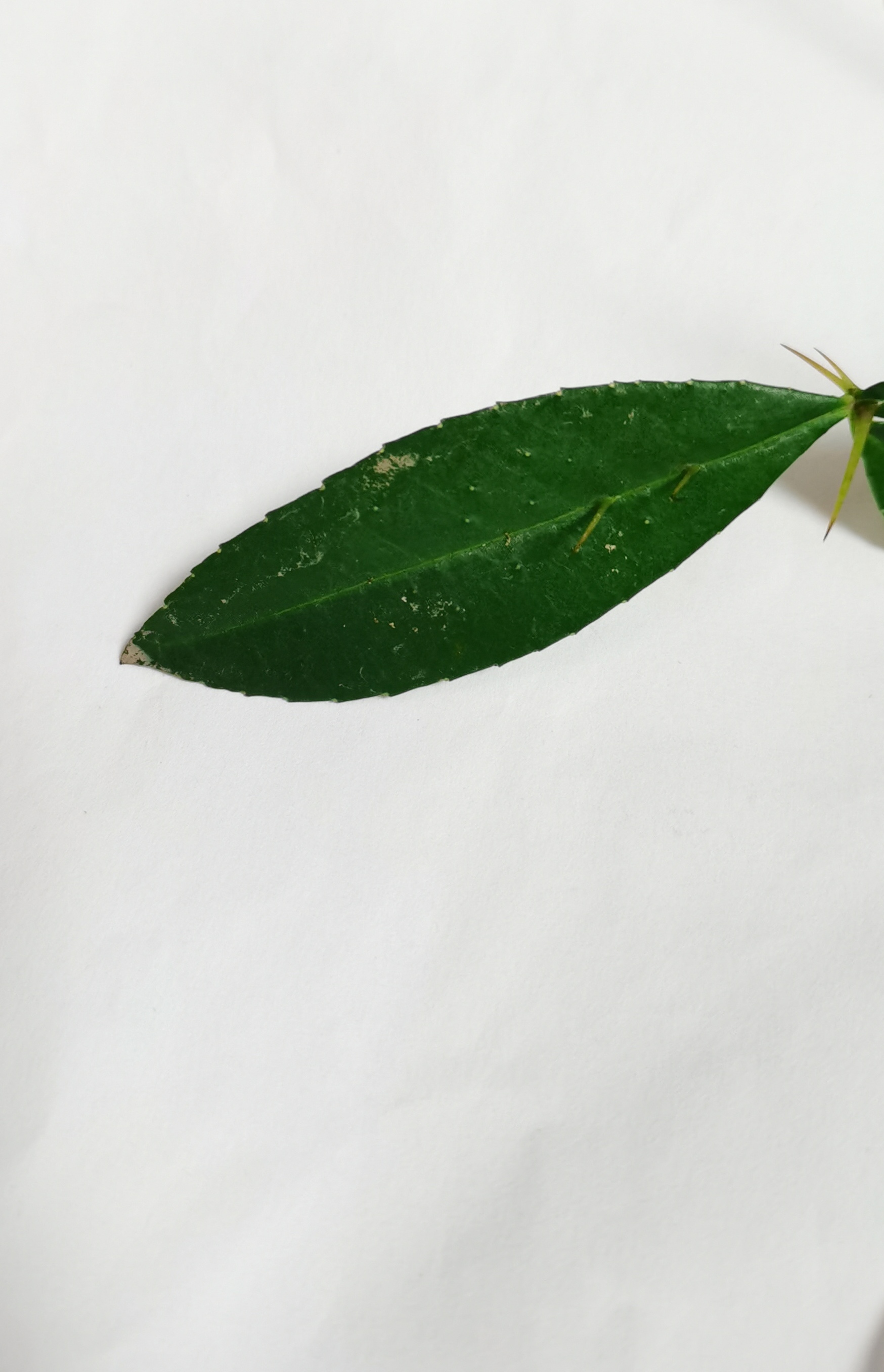

Supplement: Supplementary file 1 [file ijms-24-14761-s001.zip › Figure 1/Youkang-inoculated with C. zanthoxyli/IMG_20211128_195804_edit_642011195505680.jpg]

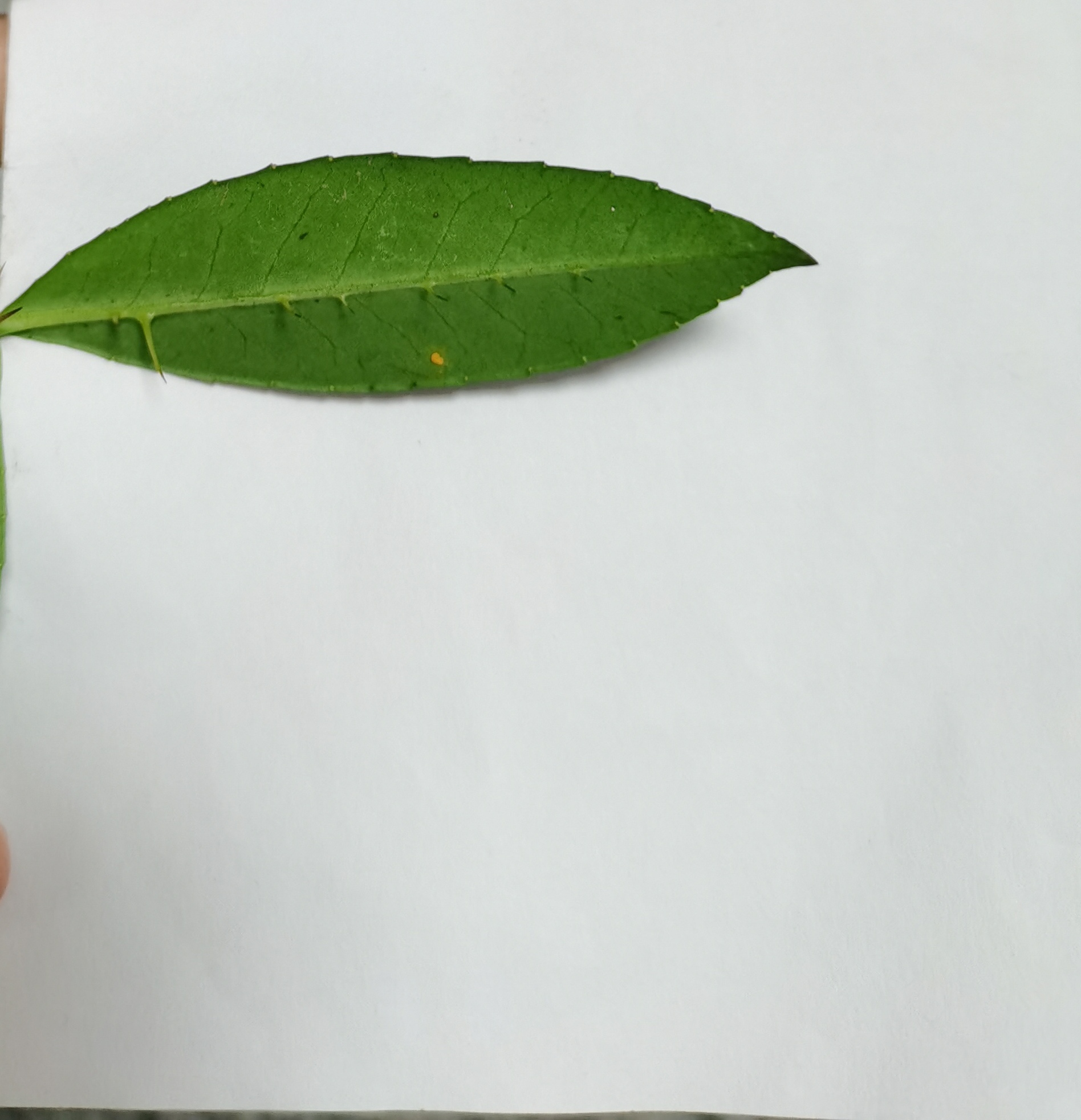

Supplement: Supplementary file 1 [file ijms-24-14761-s001.zip › Figure 1/Youkang-inoculated with C. zanthoxyli/IMG_20211128_195844_edit_642020047682242.jpg]

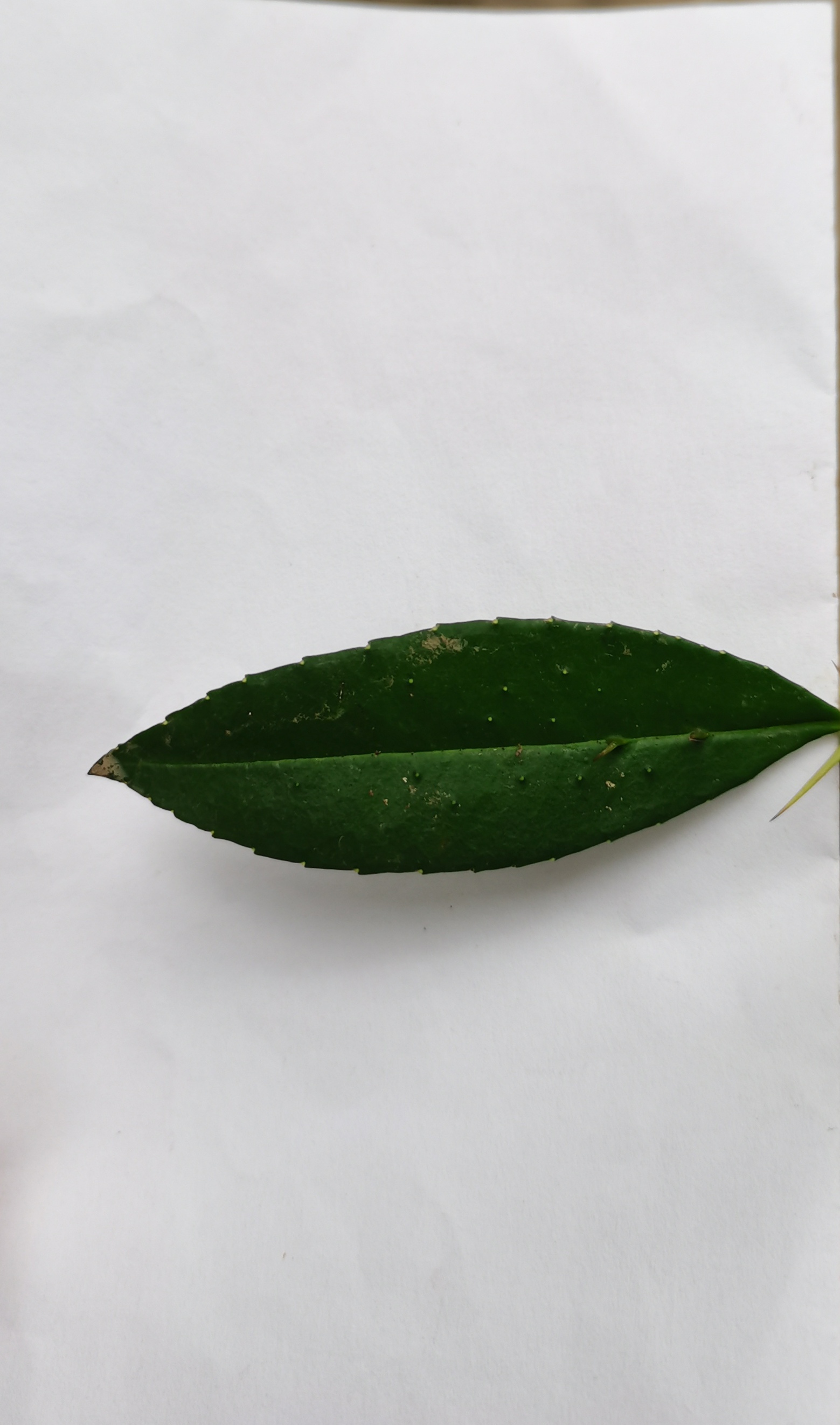

Supplement: Supplementary file 1 [file ijms-24-14761-s001.zip › Figure 1/Youkang-inoculated with C. zanthoxyli/IMG_20211129_155928_edit_657981952075118.jpg]

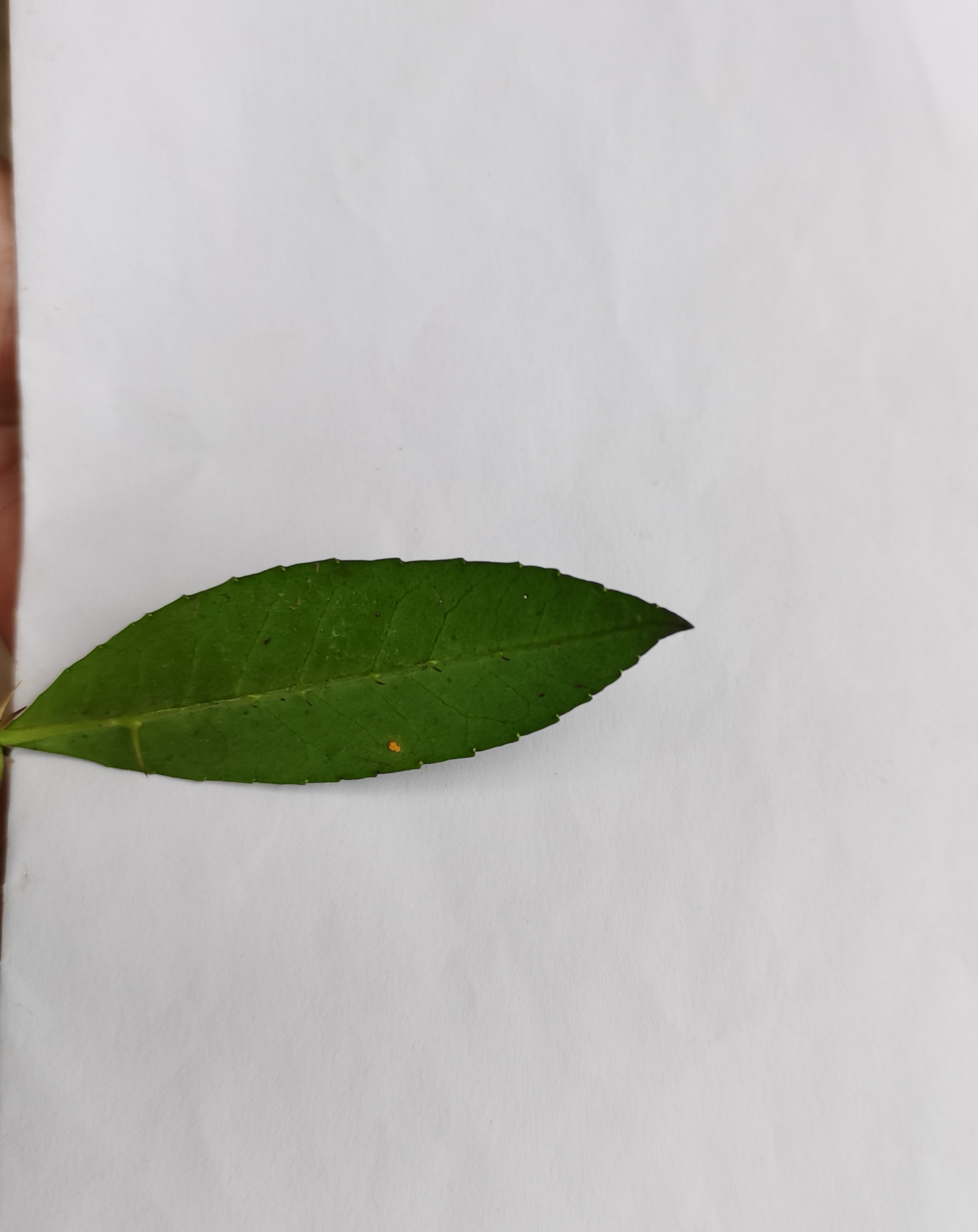

Supplement: Supplementary file 1 [file ijms-24-14761-s001.zip › Figure 1/Youkang-inoculated with C. zanthoxyli/IMG_20211129_160004_edit_657996390673033.jpg]

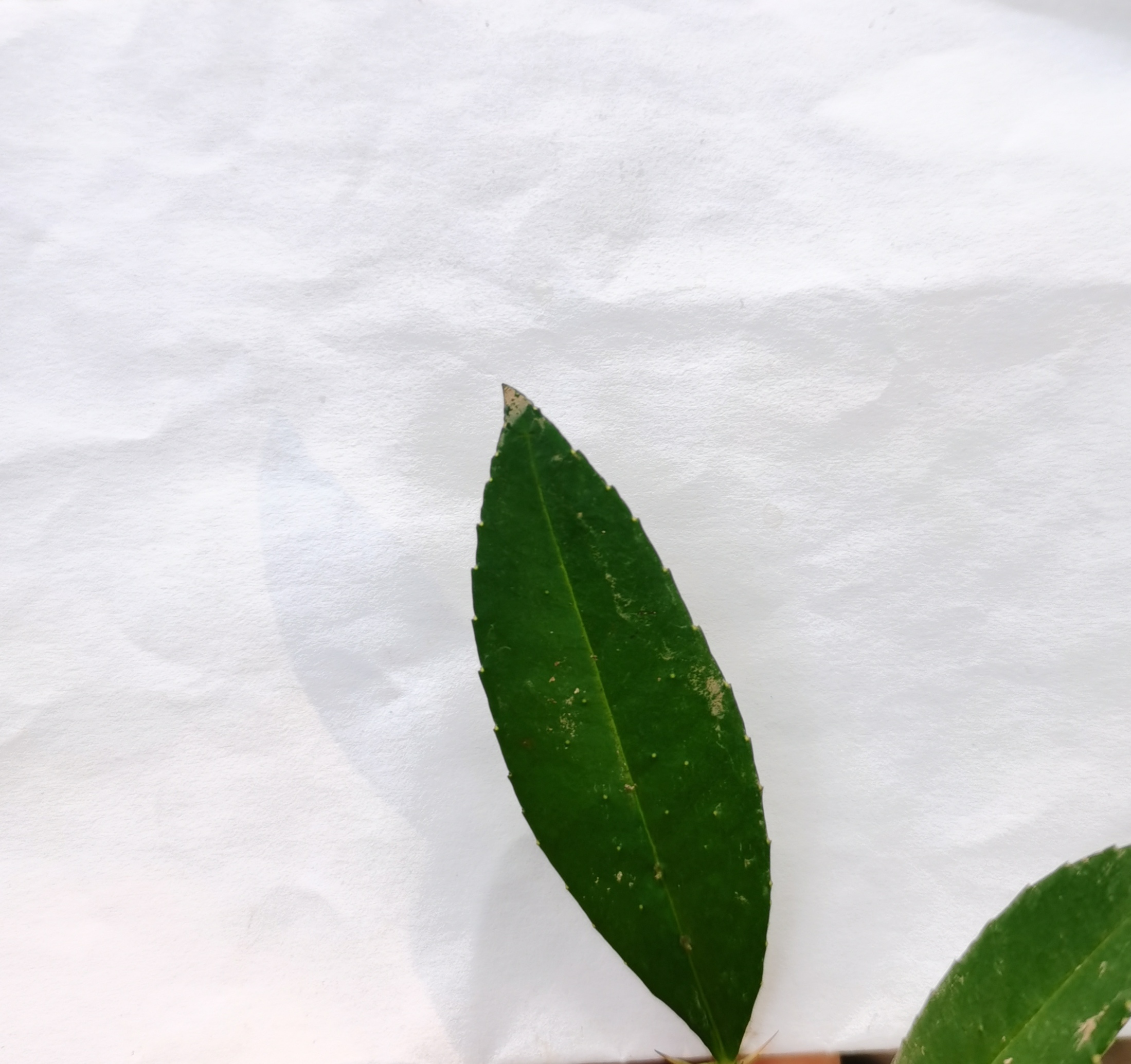

Supplement: Supplementary file 1 [file ijms-24-14761-s001.zip › Figure 1/Youkang-inoculated with C. zanthoxyli/IMG_20211202_144103_edit_756883656233985.jpg]

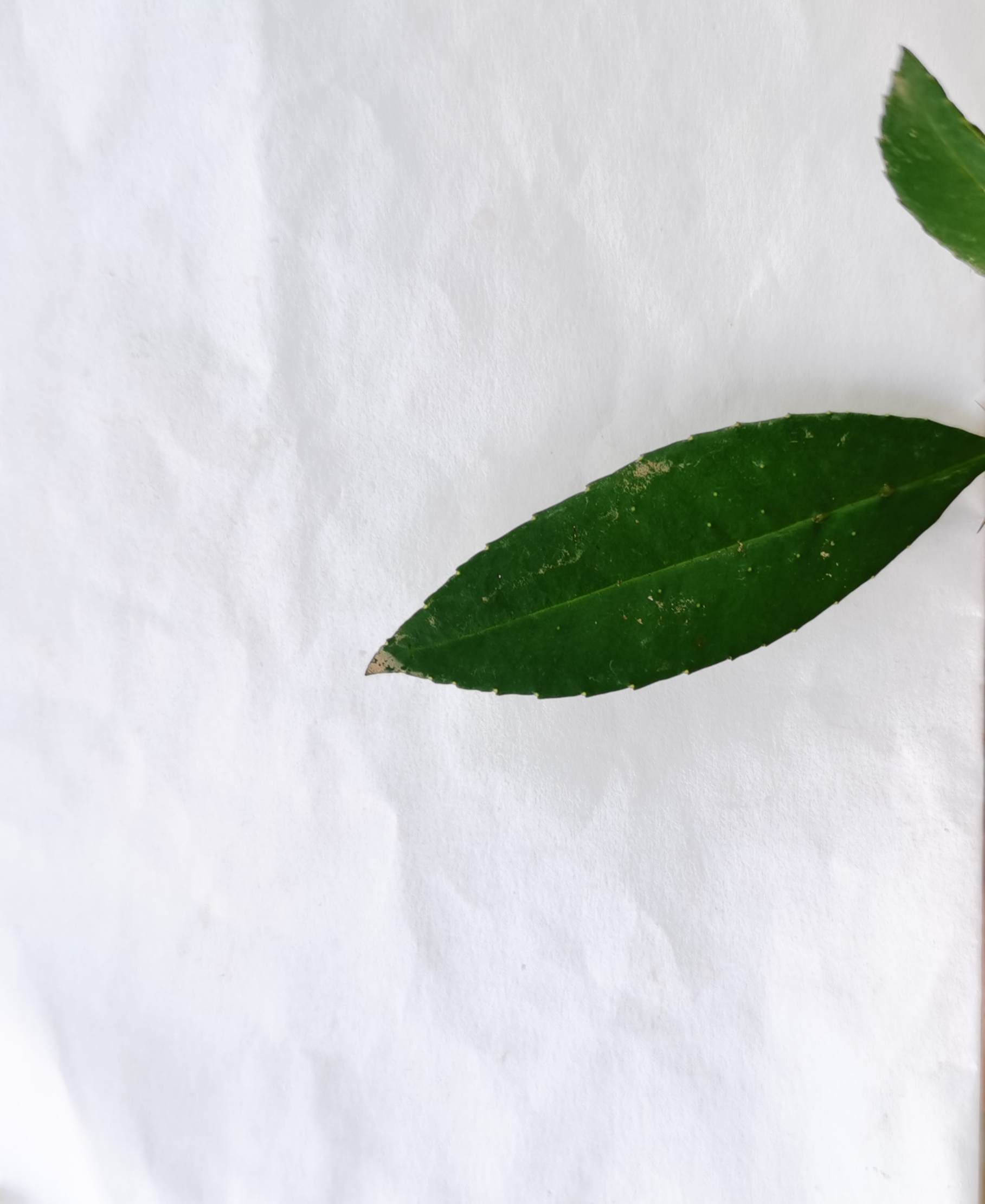

Supplement: Supplementary file 1 [file ijms-24-14761-s001.zip › Figure 1/Youkang-inoculated with C. zanthoxyli/IMG_20211202_144115_edit_757018152265735.jpg]

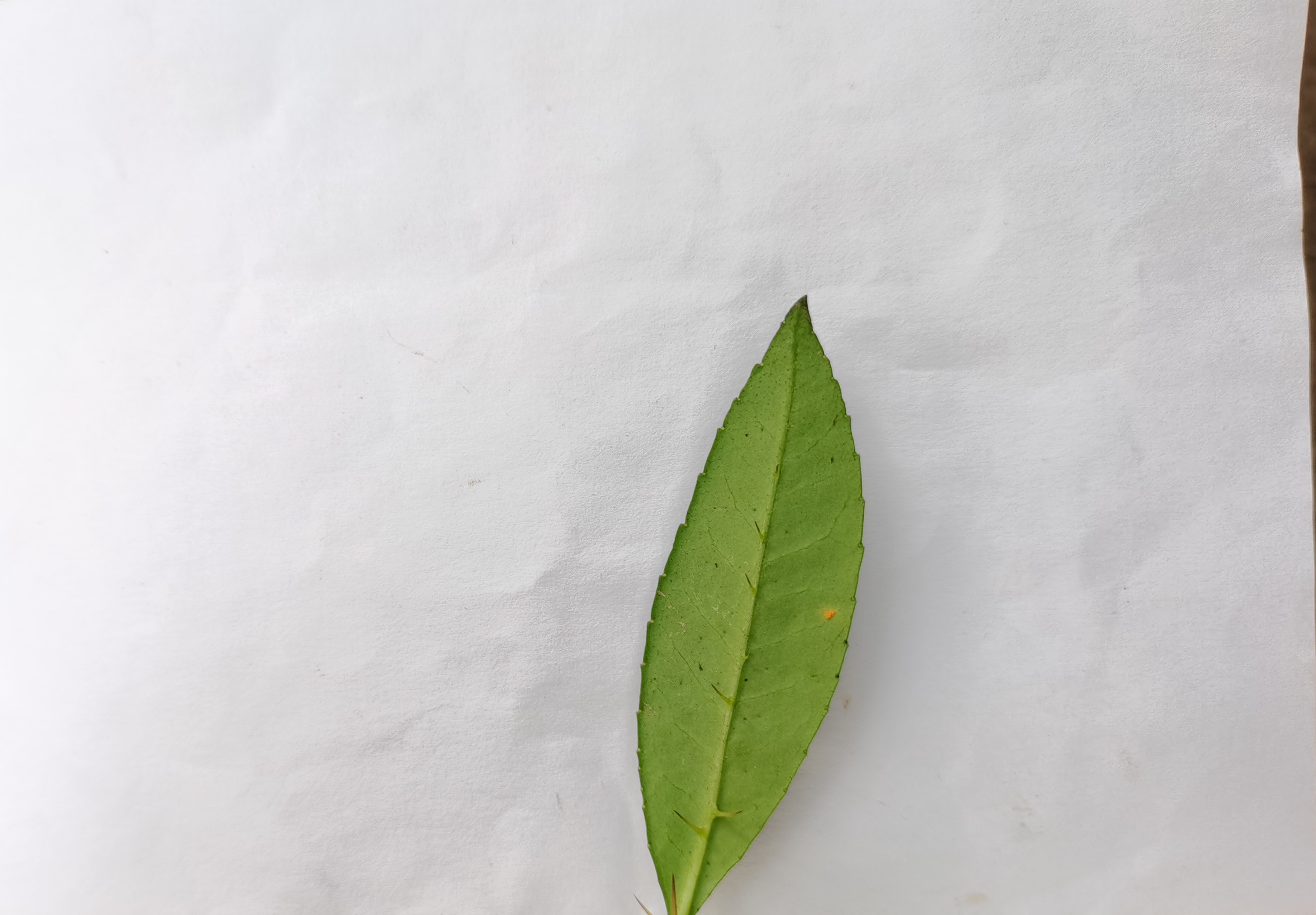

Supplement: Supplementary file 1 [file ijms-24-14761-s001.zip › Figure 1/Youkang-inoculated with C. zanthoxyli/IMG_20211202_144212_edit_757036637760524.jpg]

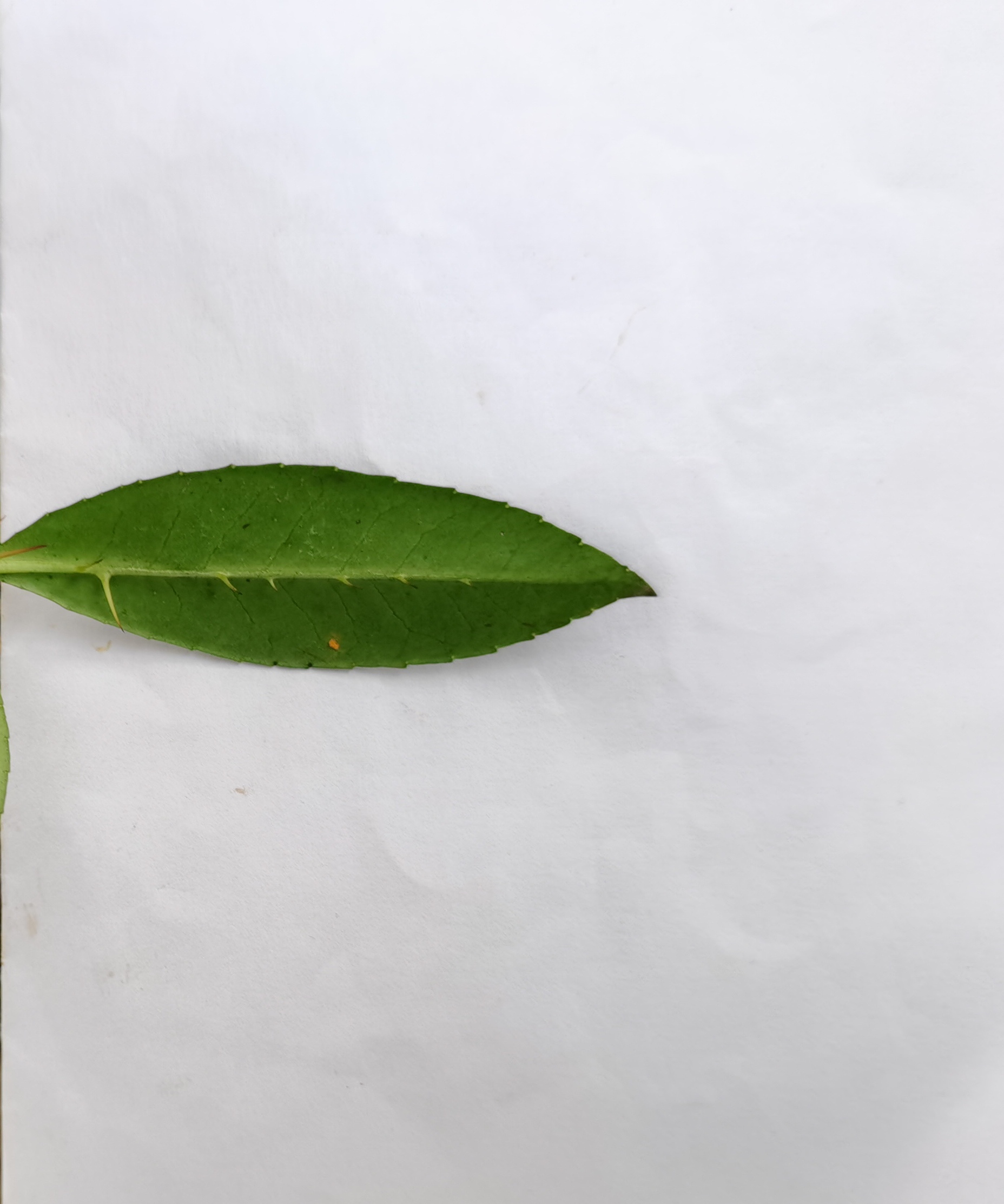

Supplement: Supplementary file 1 [file ijms-24-14761-s001.zip › Figure 1/Youkang-inoculated with C. zanthoxyli/IMG_20211202_144226_edit_757054765222500.jpg]

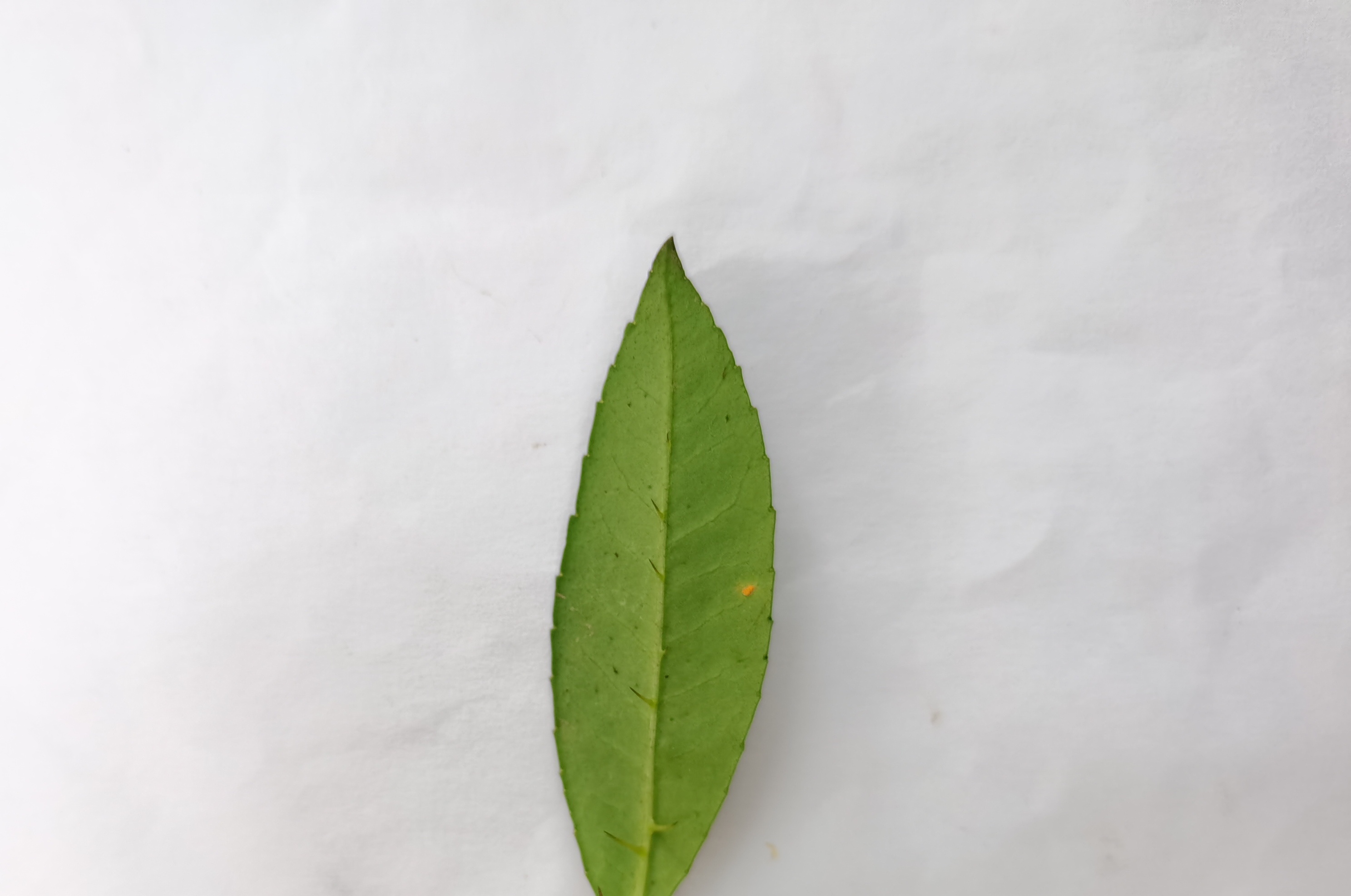

Supplement: Supplementary file 1 [file ijms-24-14761-s001.zip › Figure 1/Youkang-inoculated with C. zanthoxyli/IMG_20211202_144243_edit_757074131344893.jpg]

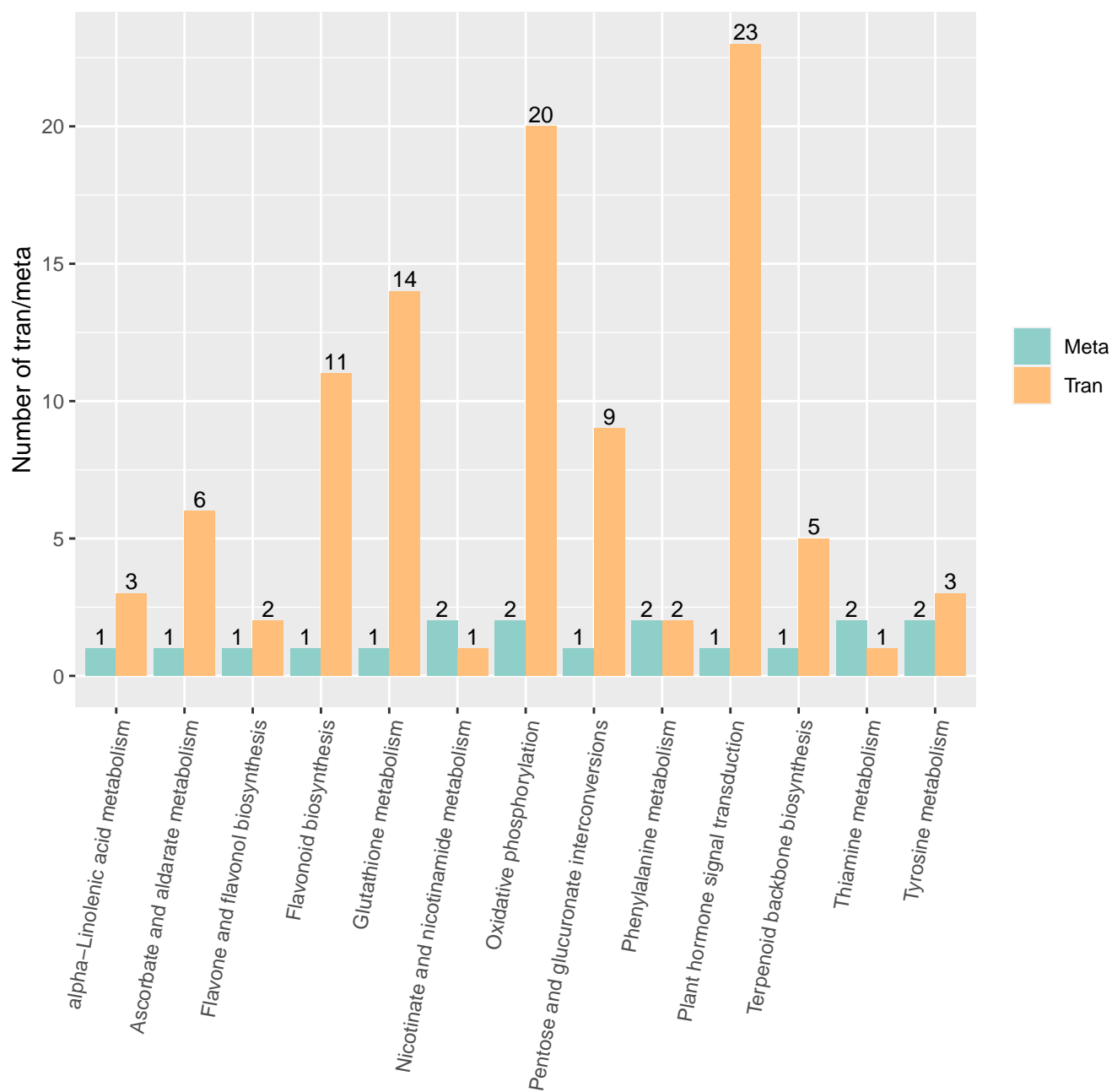

Supplement: Supplementary file 1 [file ijms-24-14761-s001.zip › Figure 10/TJ_T.vs.YK_T_neg_TJ_TvsYK_T_bar.pdf]

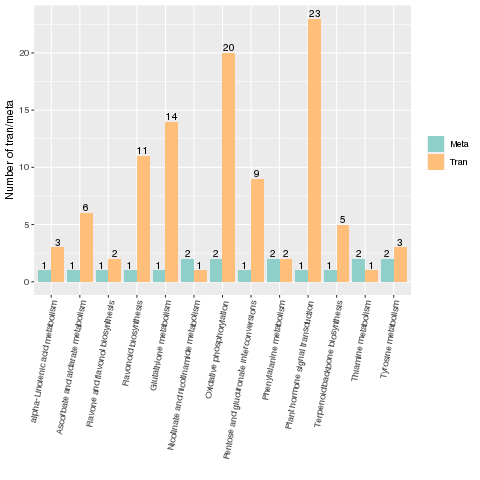

Supplement: Supplementary file 1 [file ijms-24-14761-s001.zip › Figure 10/TJ_T.vs.YK_T_neg_TJ_TvsYK_T_bar.png]

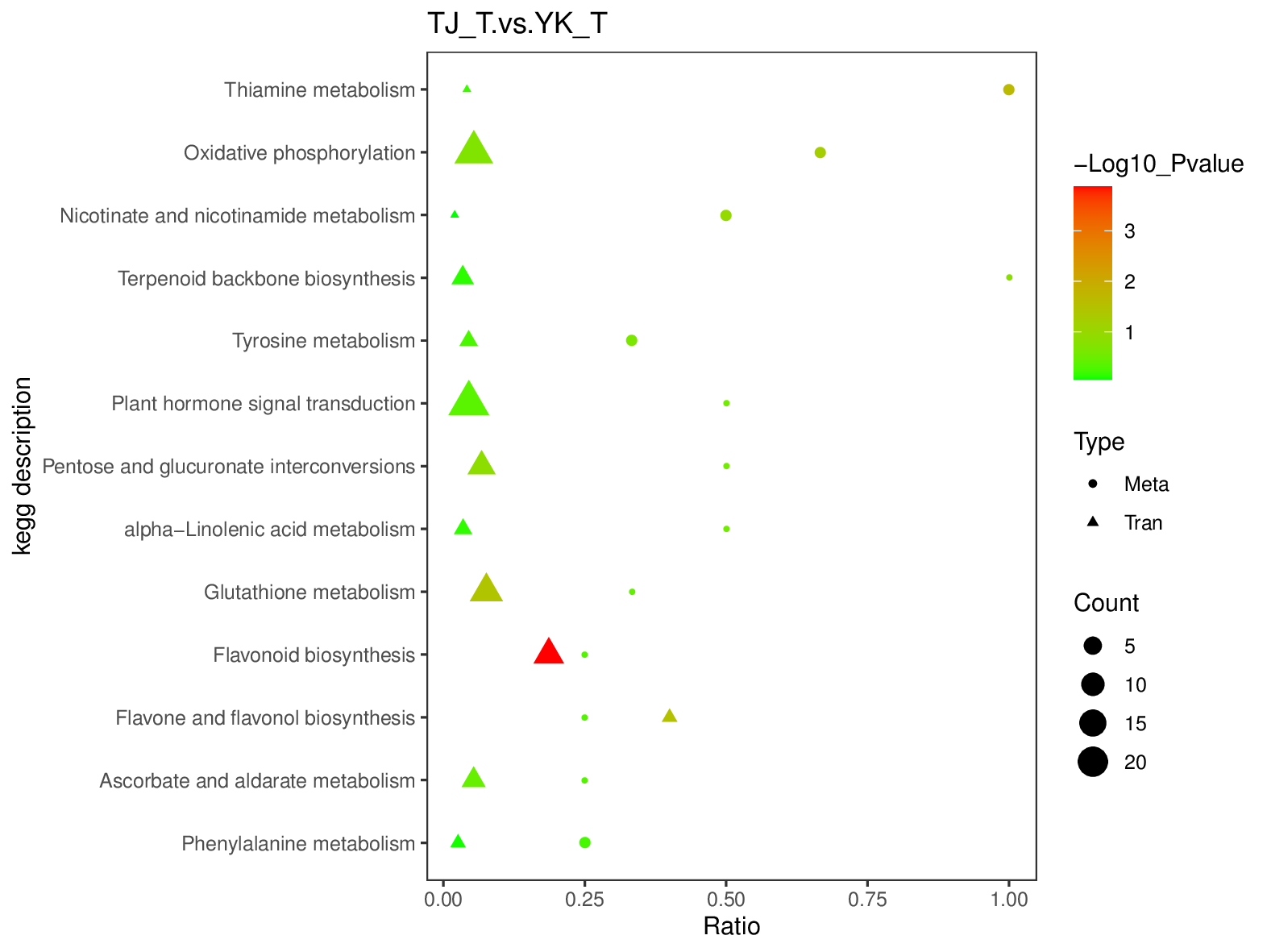

Supplement: Supplementary file 1 [file ijms-24-14761-s001.zip › Figure 10/TJ_T.vs.YK_T_neg_TJ_TvsYK_T_kegg_enrichment_share.scatterplot.dv.png]

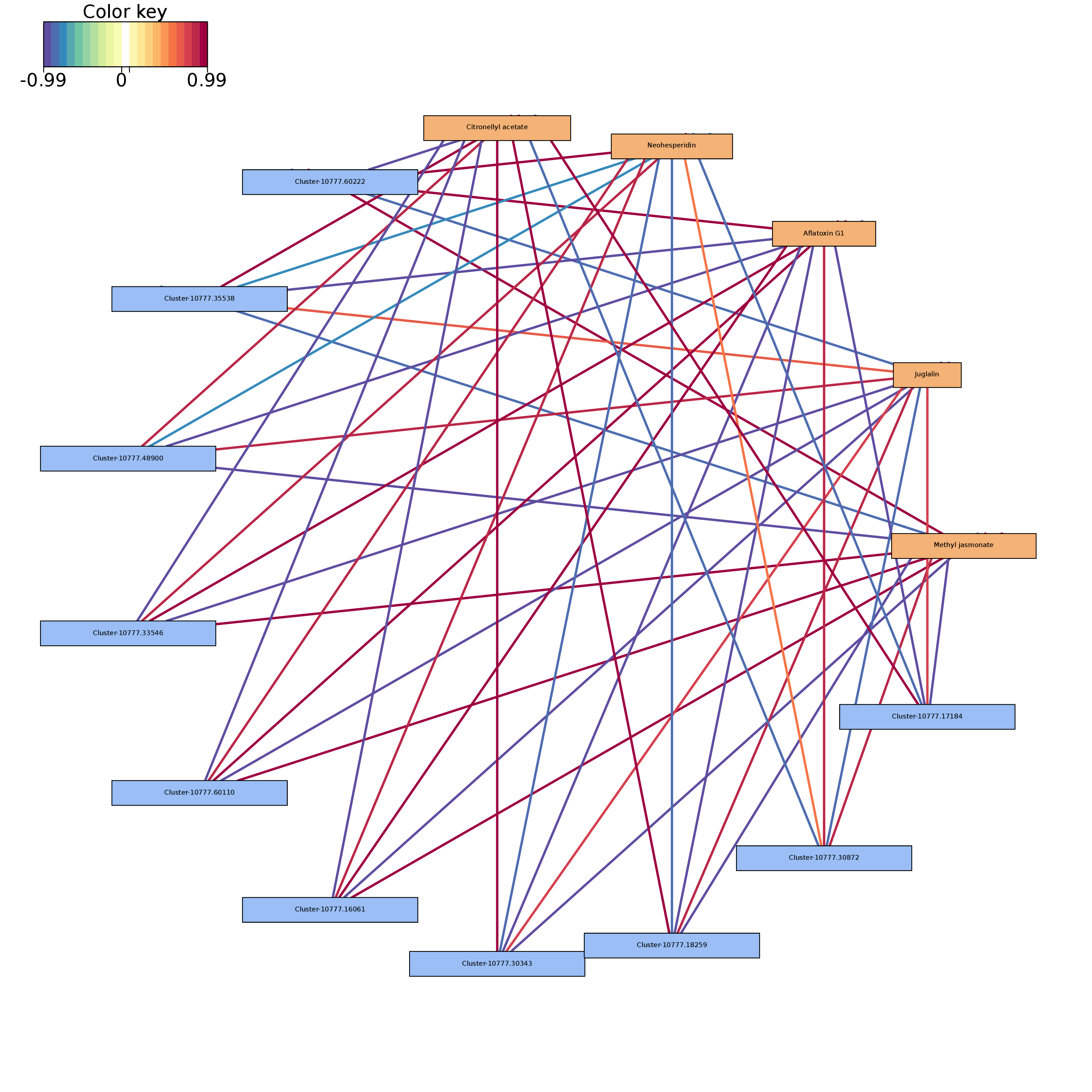

Supplement: Supplementary file 1 [file ijms-24-14761-s001.zip › Figure 10/TJ_T.vs.YK_T_neg_TJ_TvsYK_T_meta_tran_corr_net.png]

Number of tran/meta

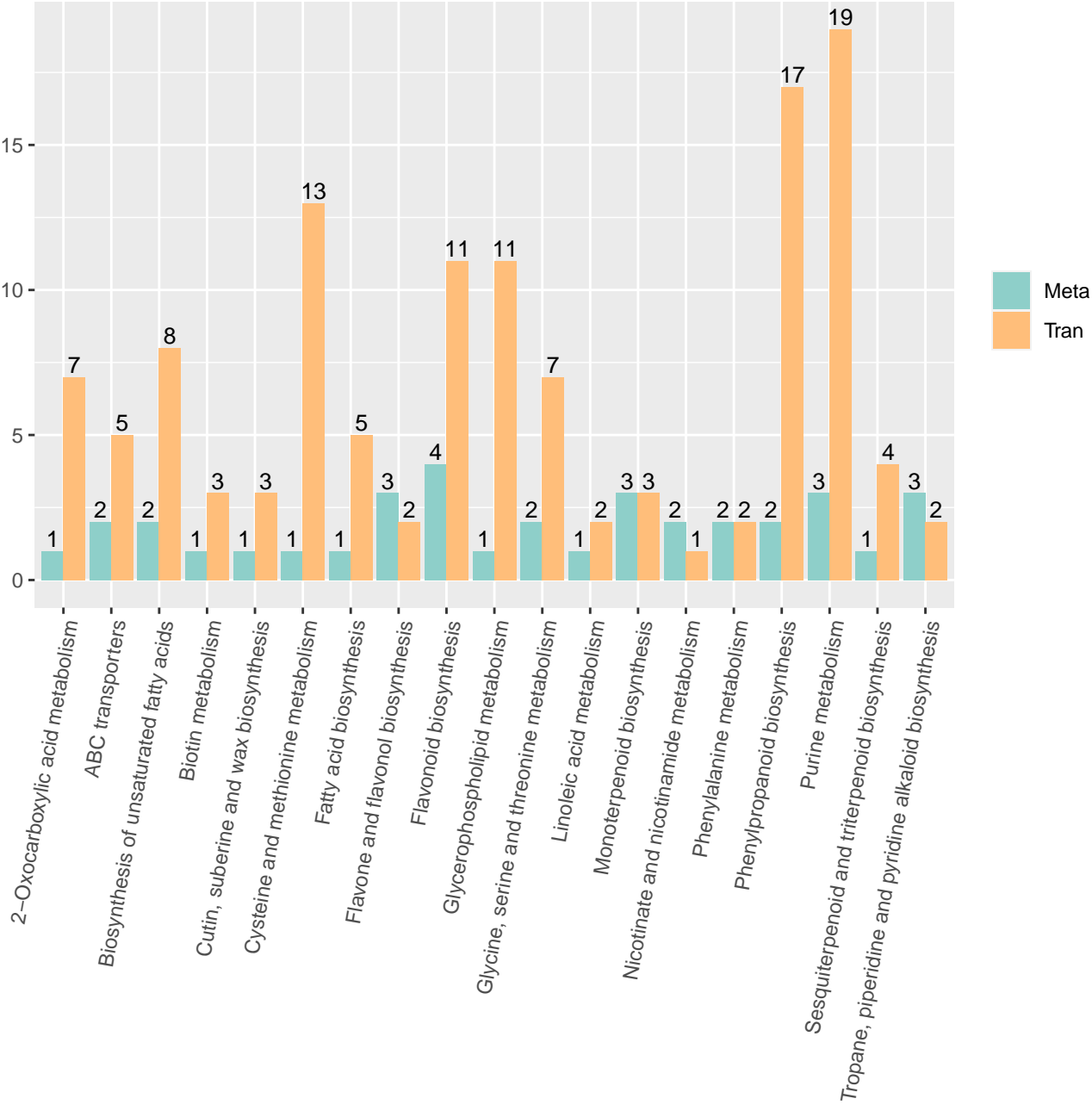

Supplement: Supplementary file 1 [file ijms-24-14761-s001.zip › Figure 10/TJ_T.vs.YK_T_pos_TJ_TvsYK_T_bar.pdf]

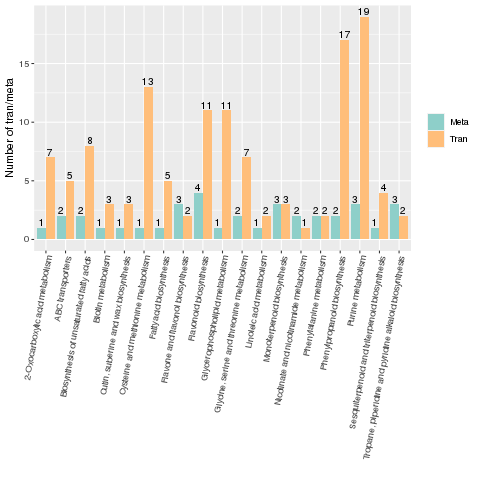

Supplement: Supplementary file 1 [file ijms-24-14761-s001.zip › Figure 10/TJ_T.vs.YK_T_pos_TJ_TvsYK_T_bar.png]

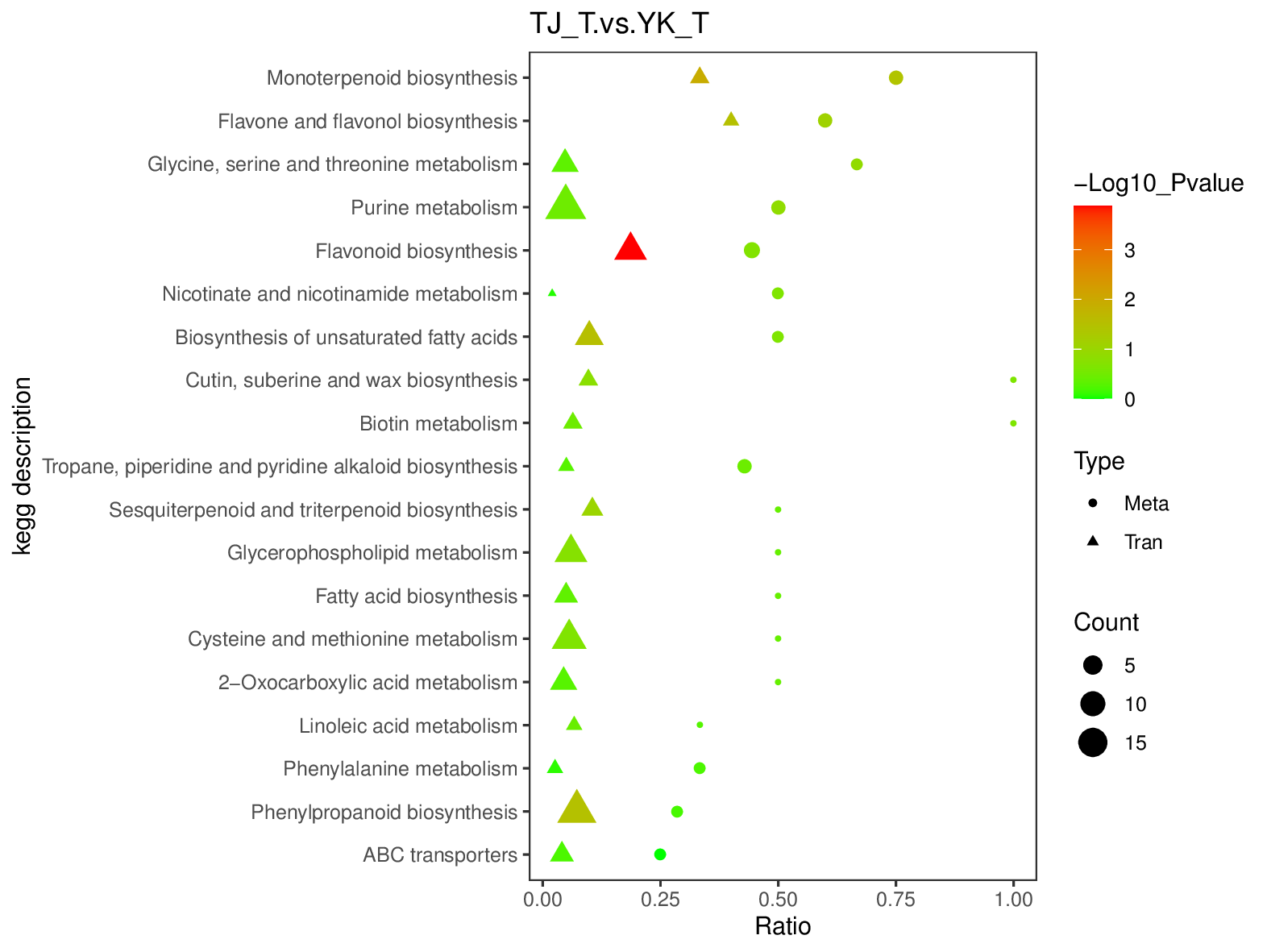

Supplement: Supplementary file 1 [file ijms-24-14761-s001.zip › Figure 10/TJ_T.vs.YK_T_pos_TJ_TvsYK_T_kegg_enrichment_share.scatterplot.dv.png]

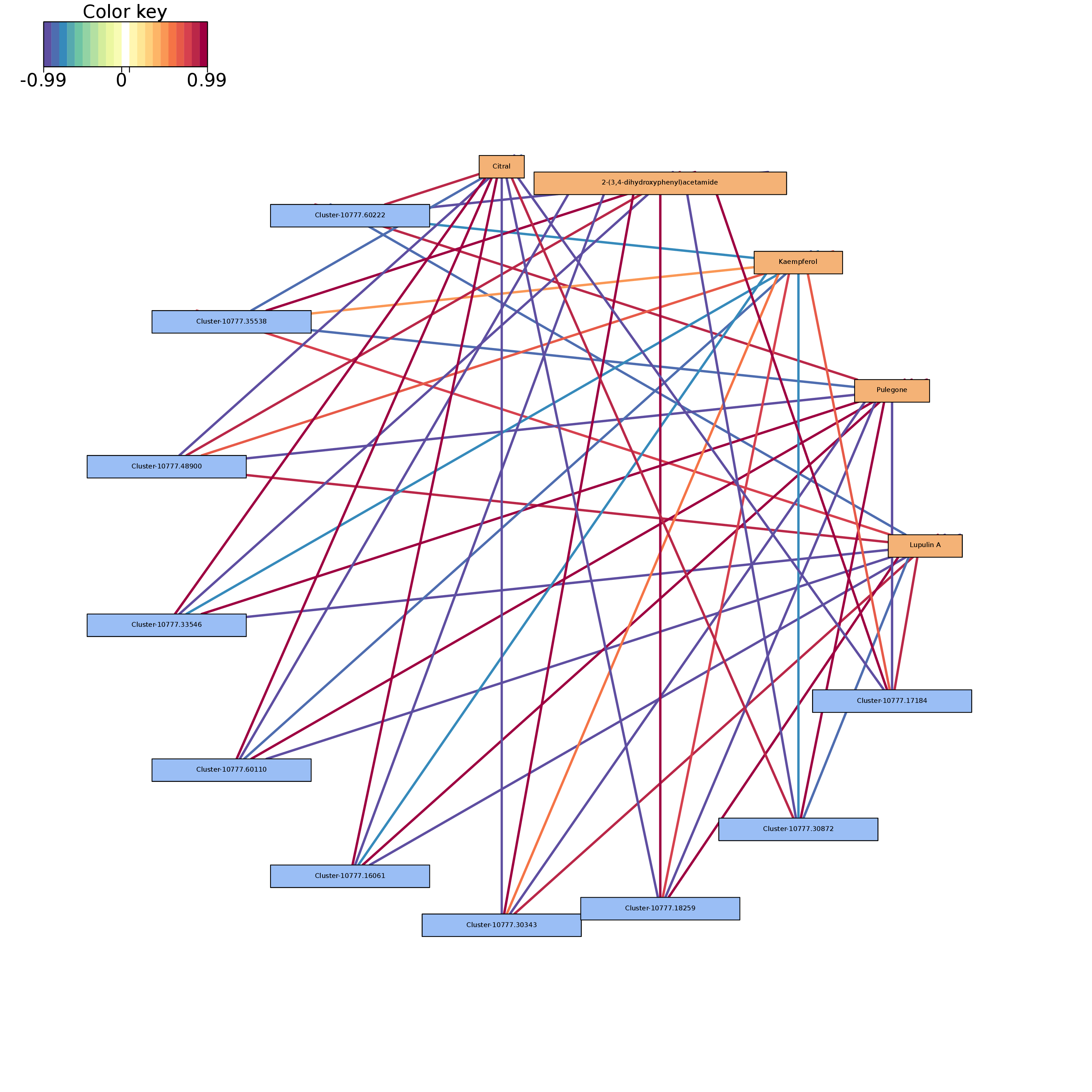

Supplement: Supplementary file 1 [file ijms-24-14761-s001.zip › Figure 10/TJ_T.vs.YK_T_pos_TJ_TvsYK_T_meta_tran_corr_net.png]

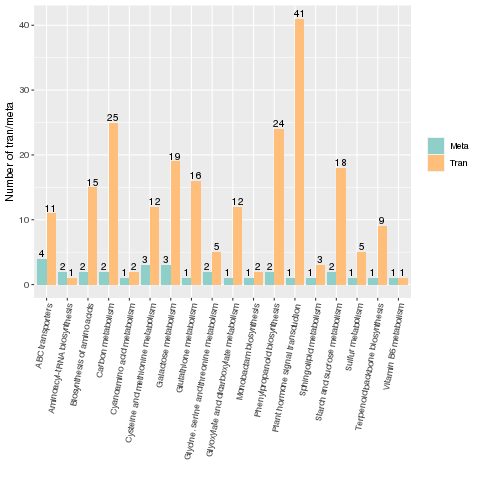

Supplement: Supplementary file 1 [file ijms-24-14761-s001.zip › Figure 10/YK_CK.vs.YK_T_neg_YK_CKvsYK_T_bar.png]

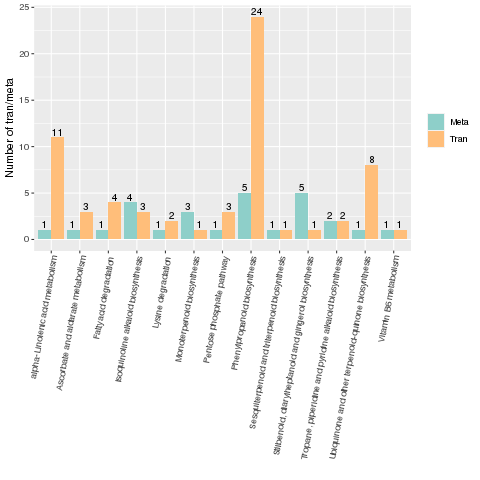

Supplement: Supplementary file 1 [file ijms-24-14761-s001.zip › Figure 10/YK_CK.vs.YK_T_pos_YK_CKvsYK_T_bar.png]

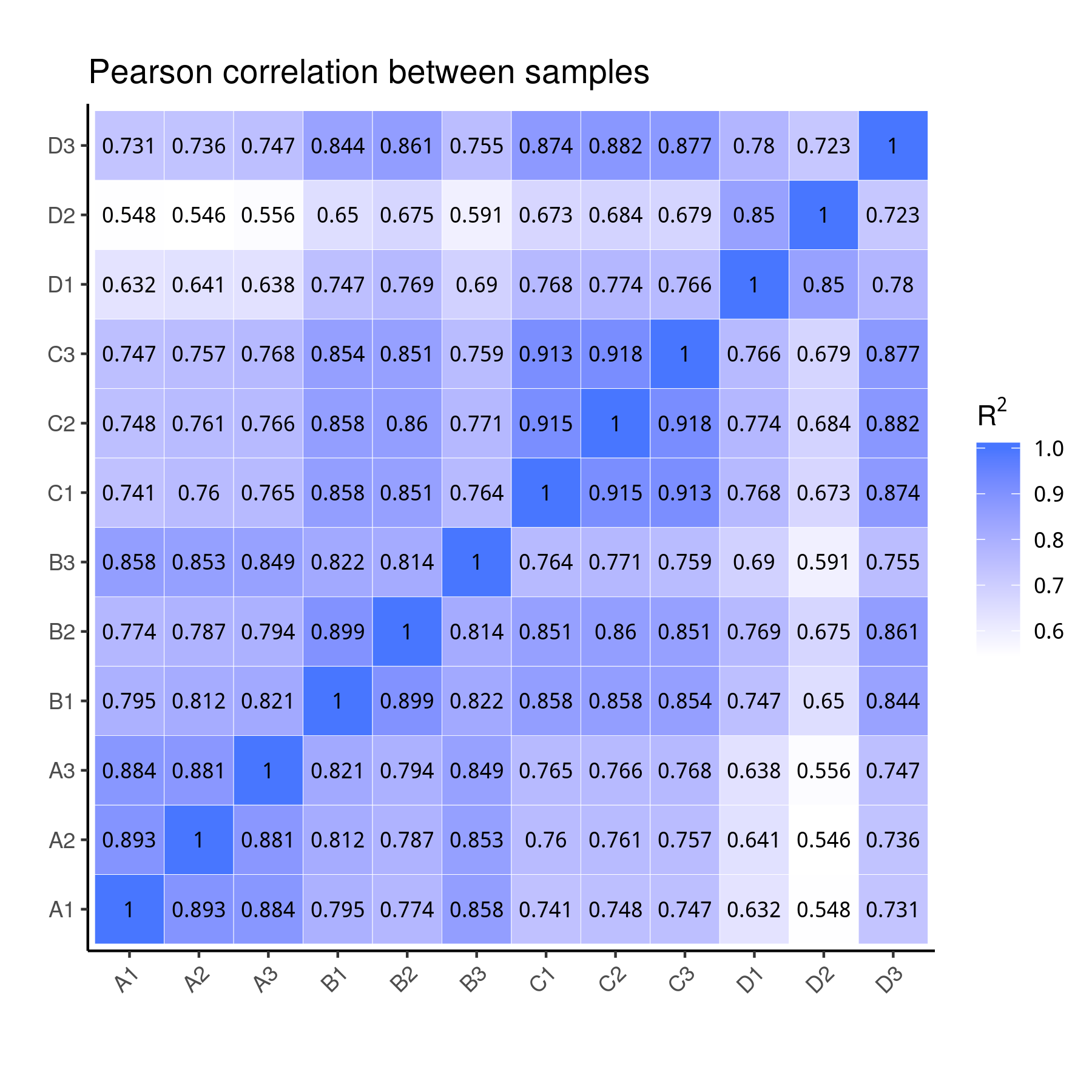

Supplement: Supplementary file 1 [file ijms-24-14761-s001.zip › Figure 2/correlation.png]

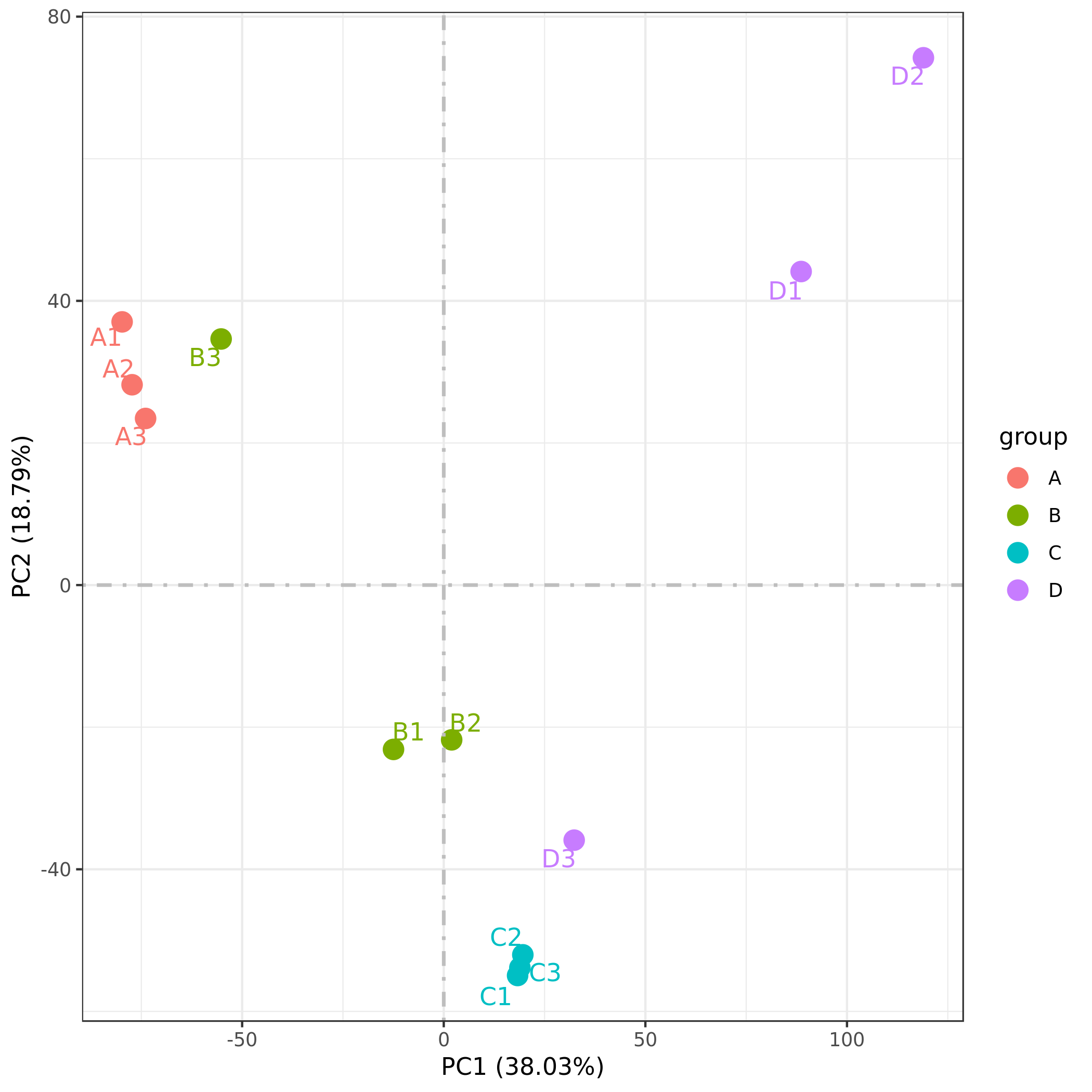

Supplement: Supplementary file 1 [file ijms-24-14761-s001.zip › Figure 2/PCA2_PCA2D.png]

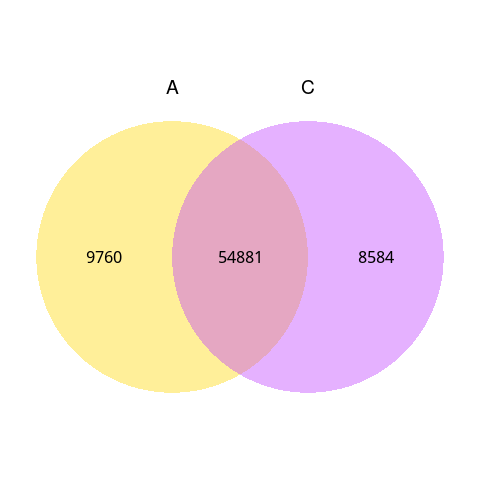

Supplement: Supplementary file 1 [file ijms-24-14761-s001.zip › Figure 3/A_C.png]

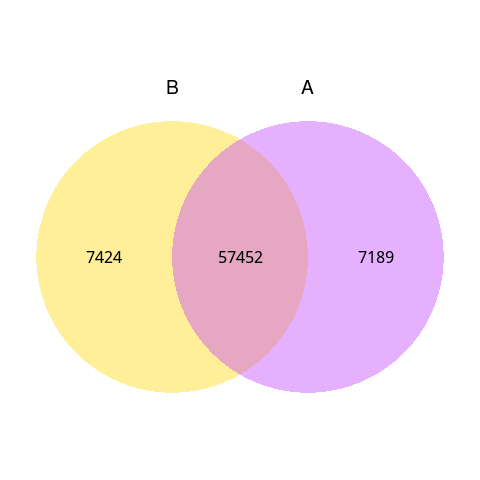

Supplement: Supplementary file 1 [file ijms-24-14761-s001.zip › Figure 3/B_A.png]

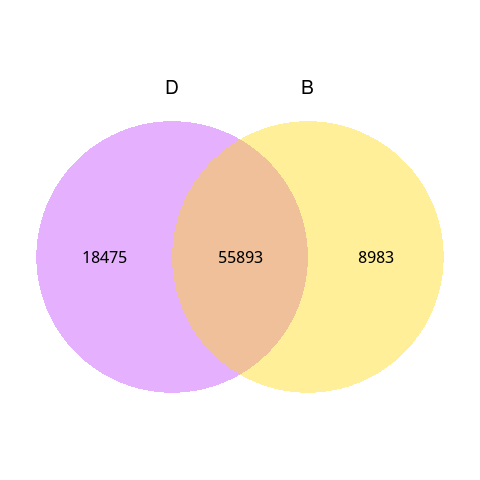

Supplement: Supplementary file 1 [file ijms-24-14761-s001.zip › Figure 3/B_D.png]

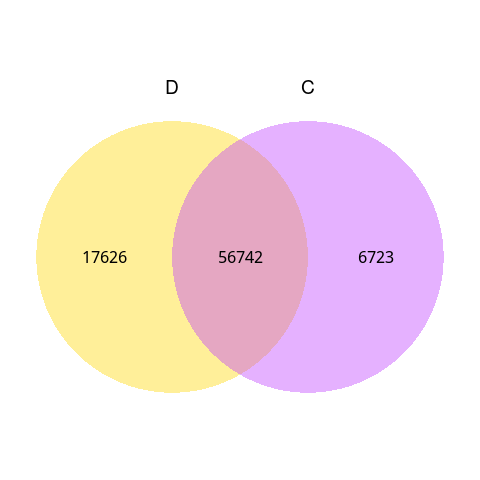

Supplement: Supplementary file 1 [file ijms-24-14761-s001.zip › Figure 3/D_C.png]

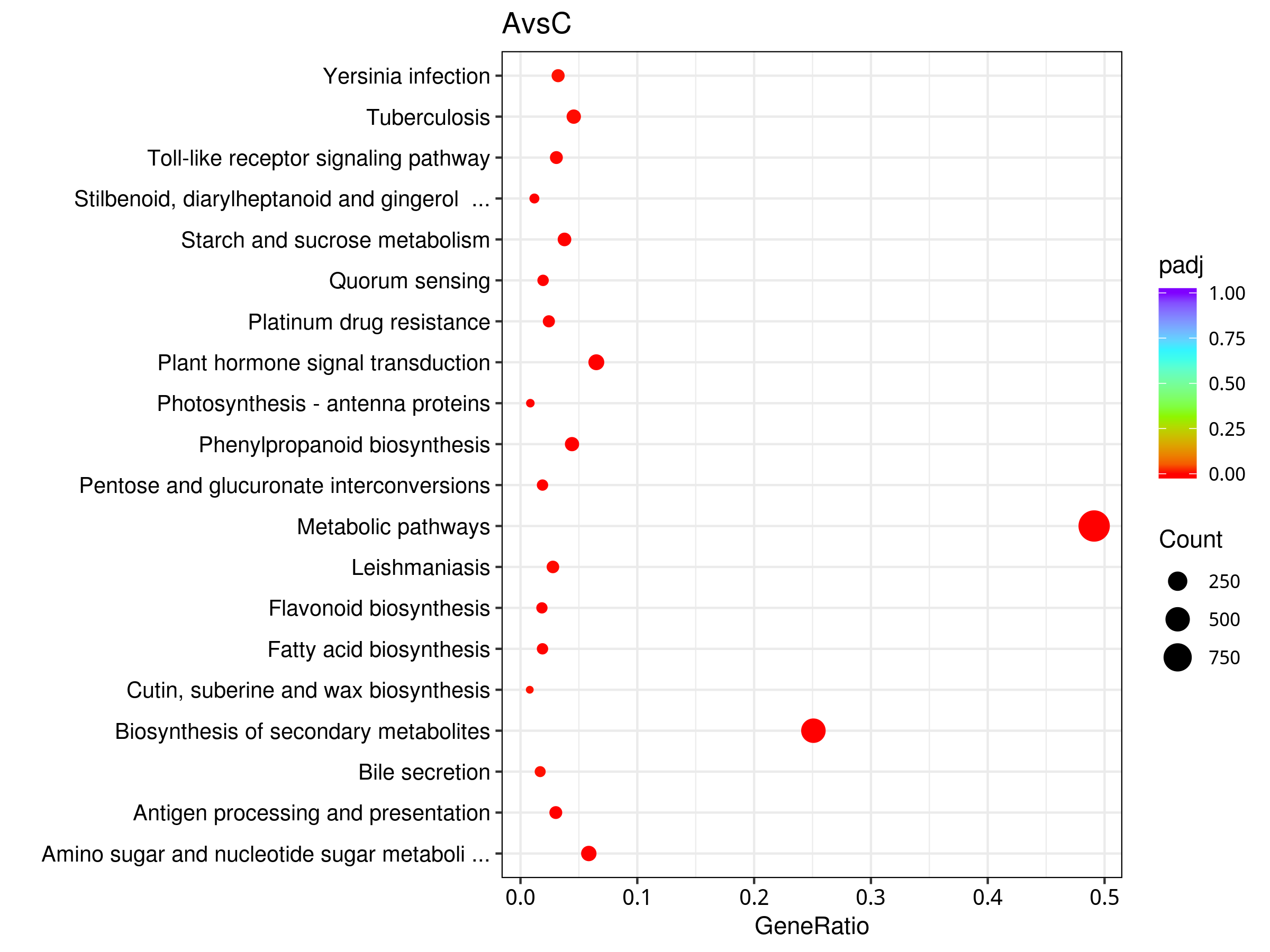

Supplement: Supplementary file 1 [file ijms-24-14761-s001.zip › Figure 4/AvsC.all_KEGG_dot.png]

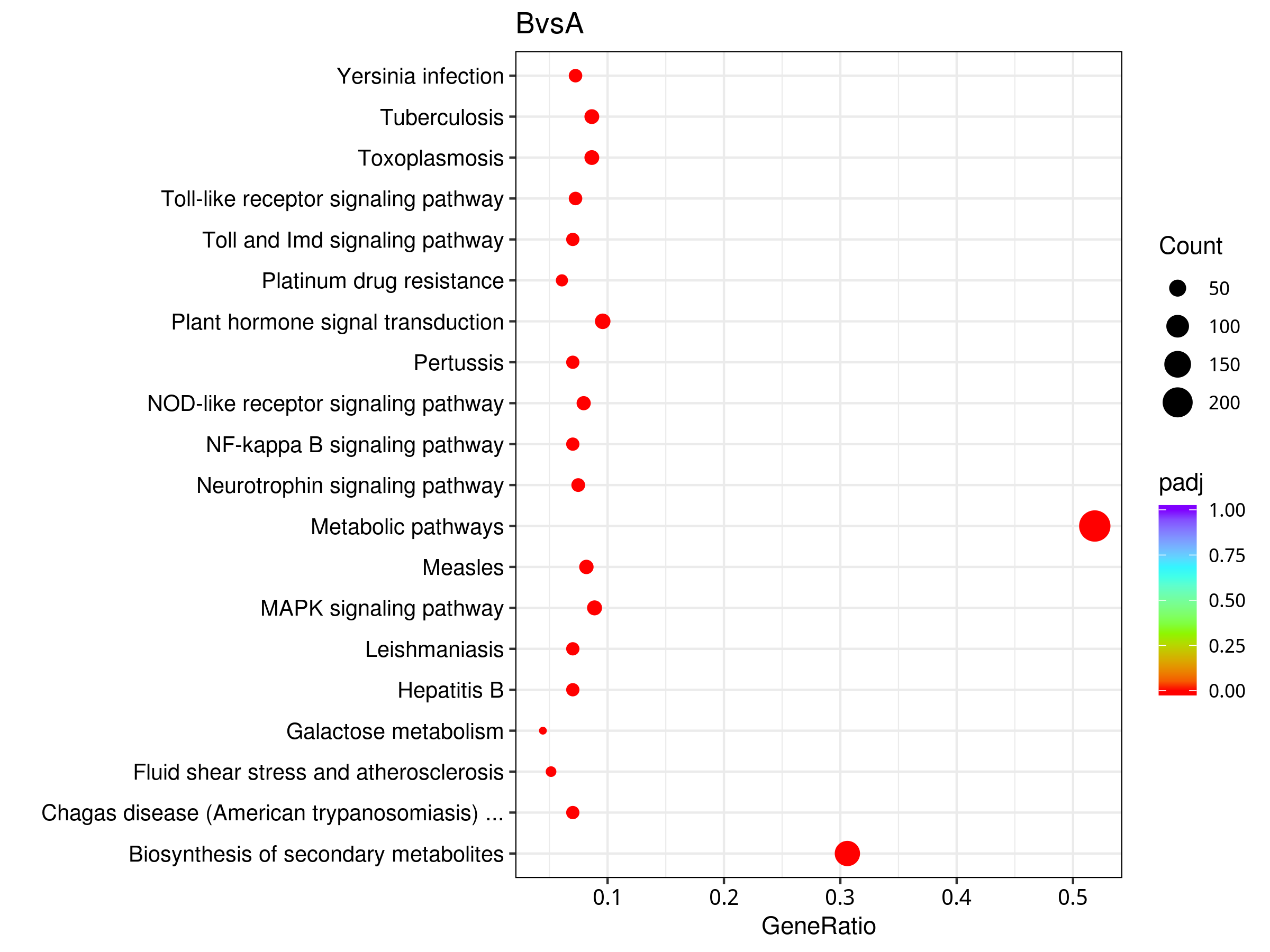

Supplement: Supplementary file 1 [file ijms-24-14761-s001.zip › Figure 4/BvsA.all_KEGG_dot.png]

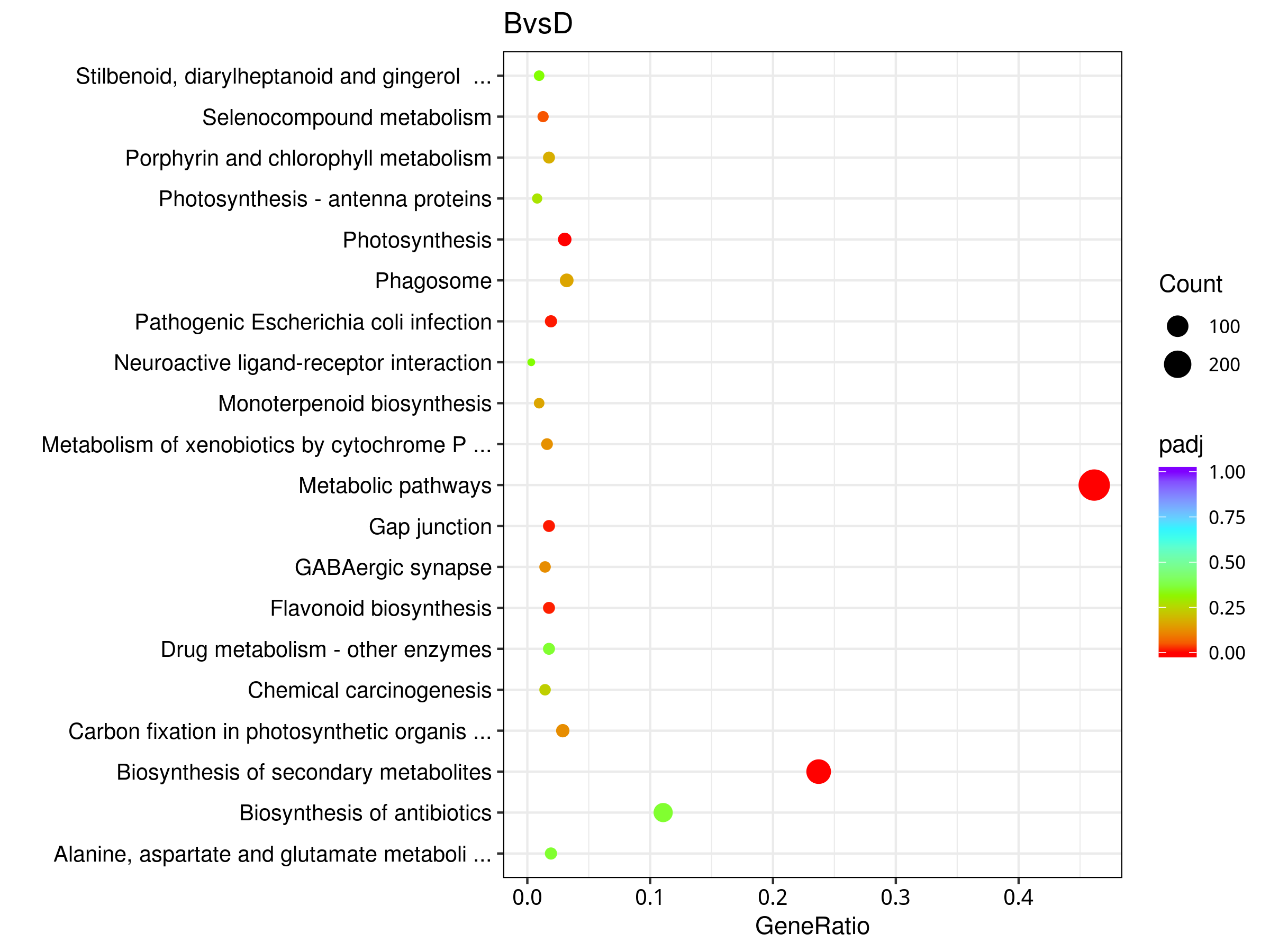

Supplement: Supplementary file 1 [file ijms-24-14761-s001.zip › Figure 4/BvsD.all_KEGG_dot.png]

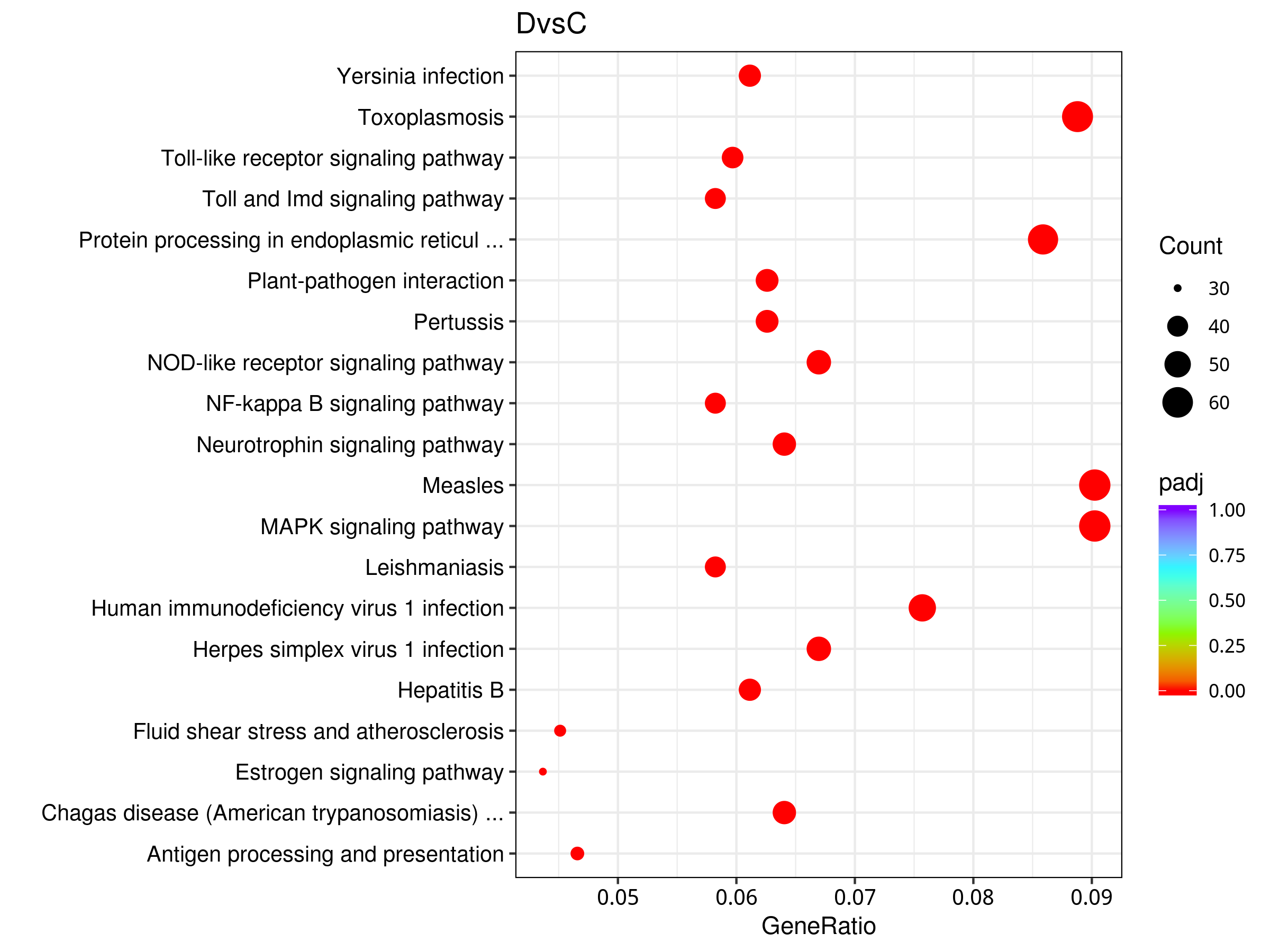

Supplement: Supplementary file 1 [file ijms-24-14761-s001.zip › Figure 4/DvsC.all_KEGG_dot.png]

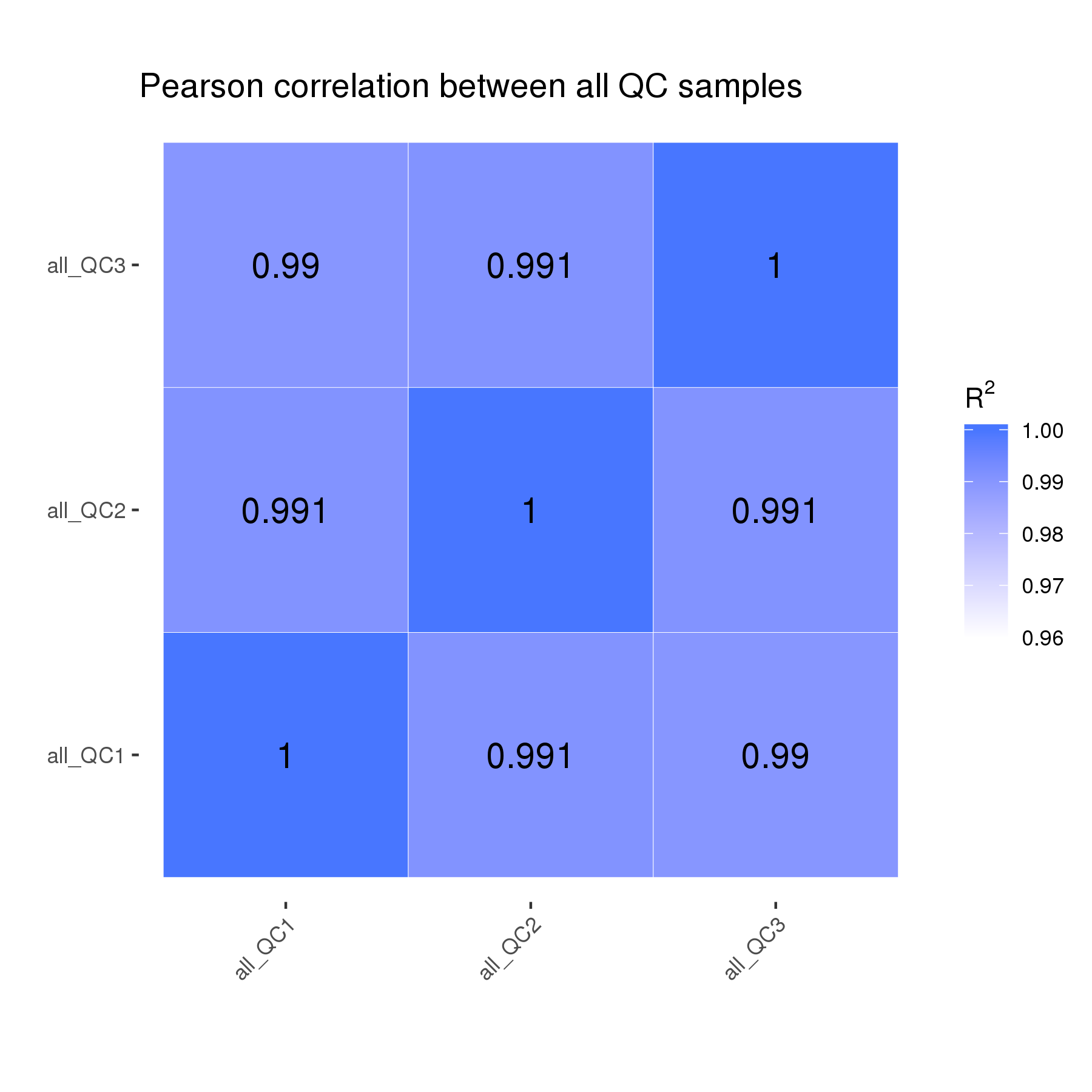

Supplement: Supplementary file 1 [file ijms-24-14761-s001.zip › Figure 6/cor_pearson_all.png]

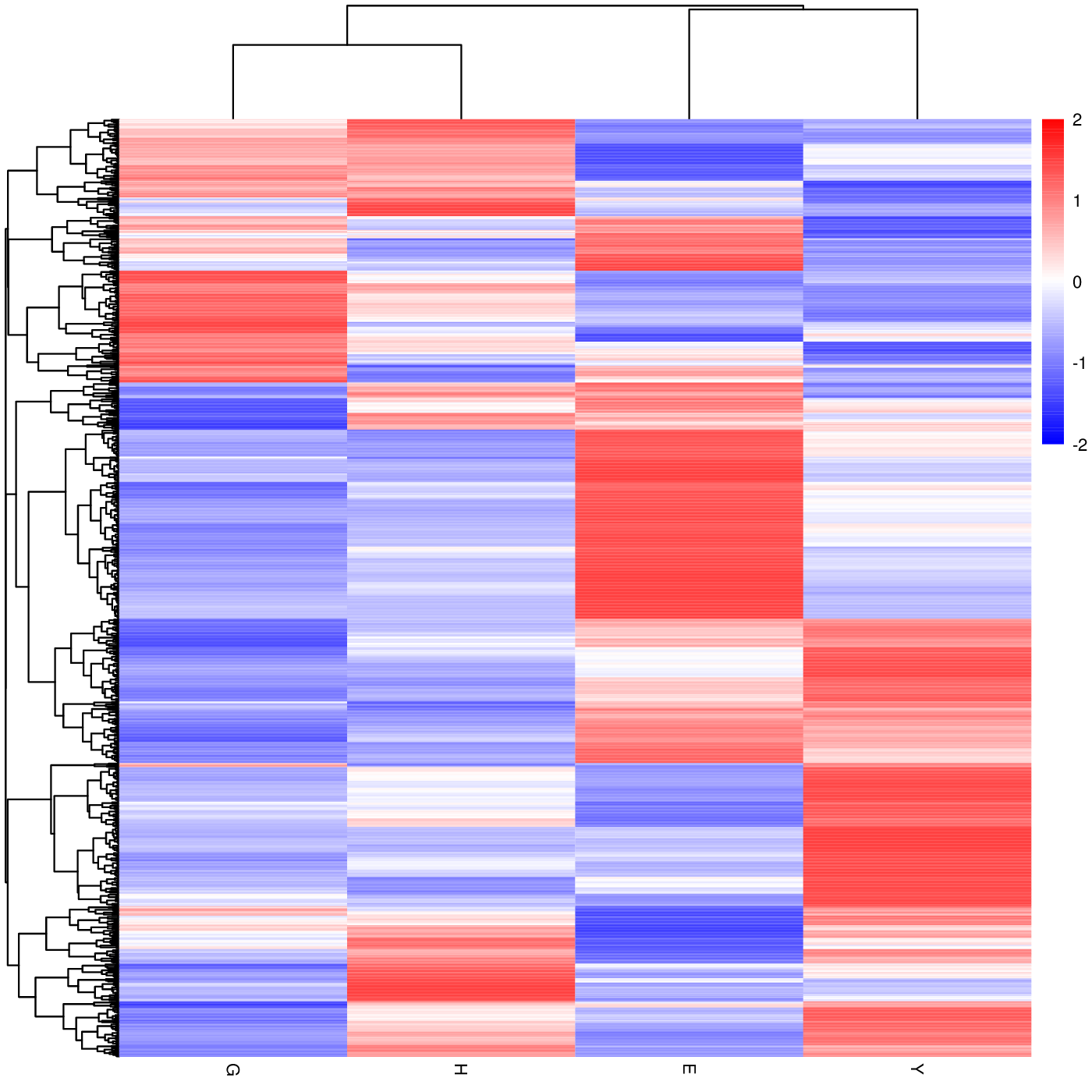

Supplement: Supplementary file 1 [file ijms-24-14761-s001.zip › Figure 6/Diff_Heatmap_all.cluster.png]

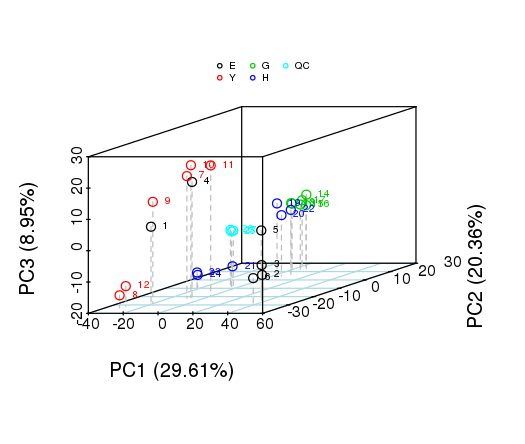

Supplement: Supplementary file 1 [file ijms-24-14761-s001.zip › Figure 6/Samples_QC_all-PCA.3D.png]

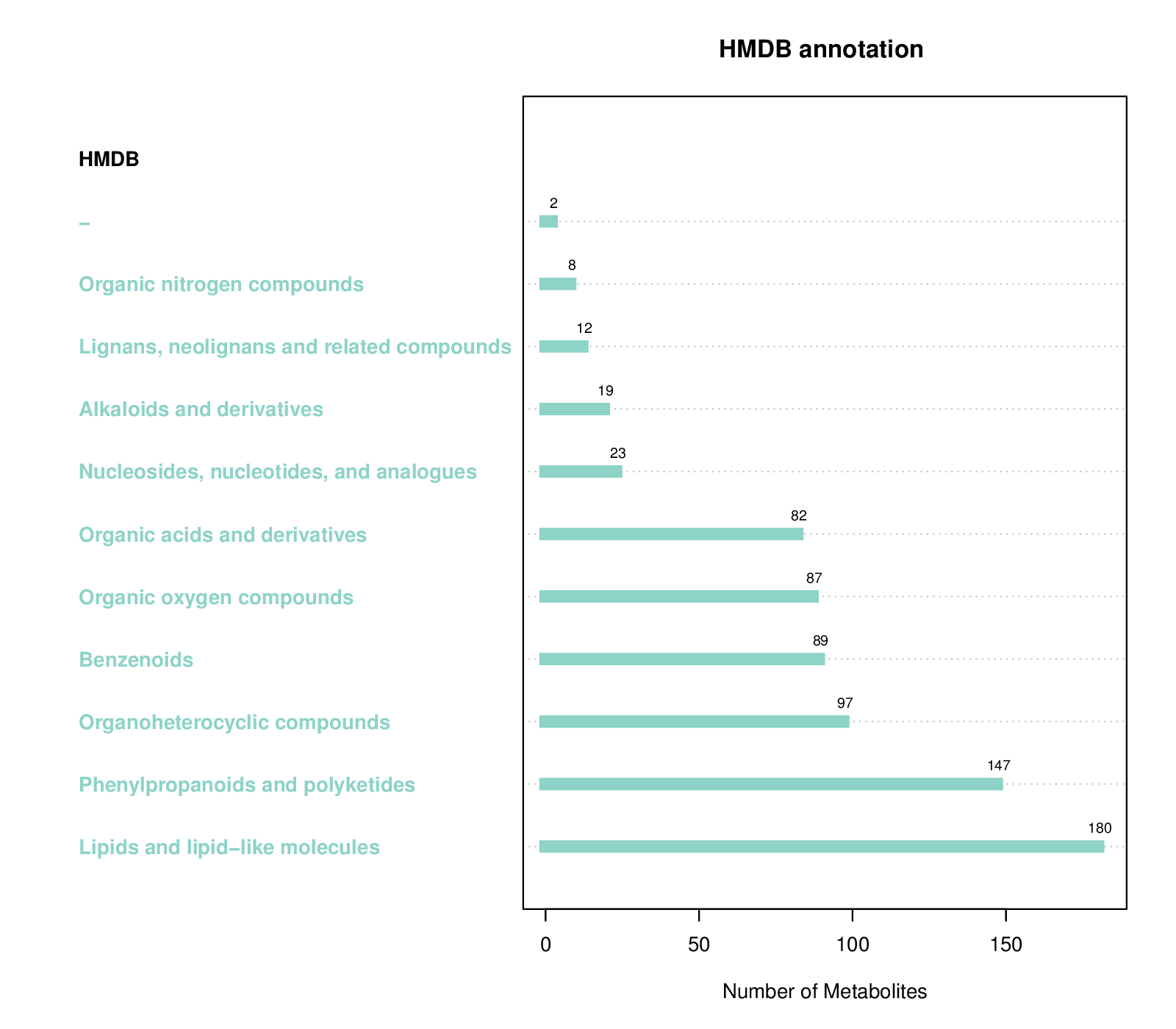

Supplement: Supplementary file 1 [file ijms-24-14761-s001.zip › Figure 7/meta_all.HMDB.Anno.png]

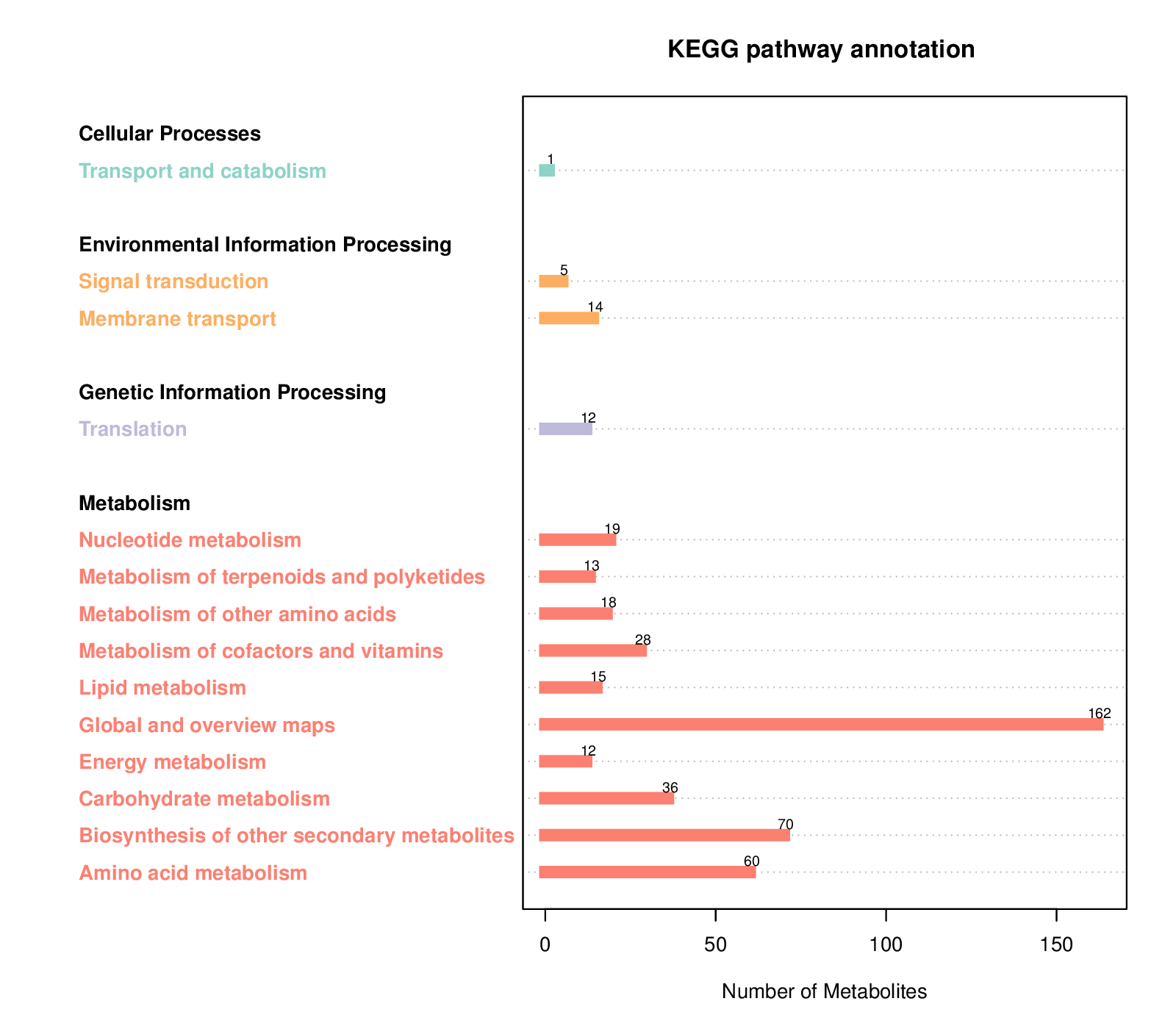

Supplement: Supplementary file 1 [file ijms-24-14761-s001.zip › Figure 7/meta_all.KEGG.Anno.png]

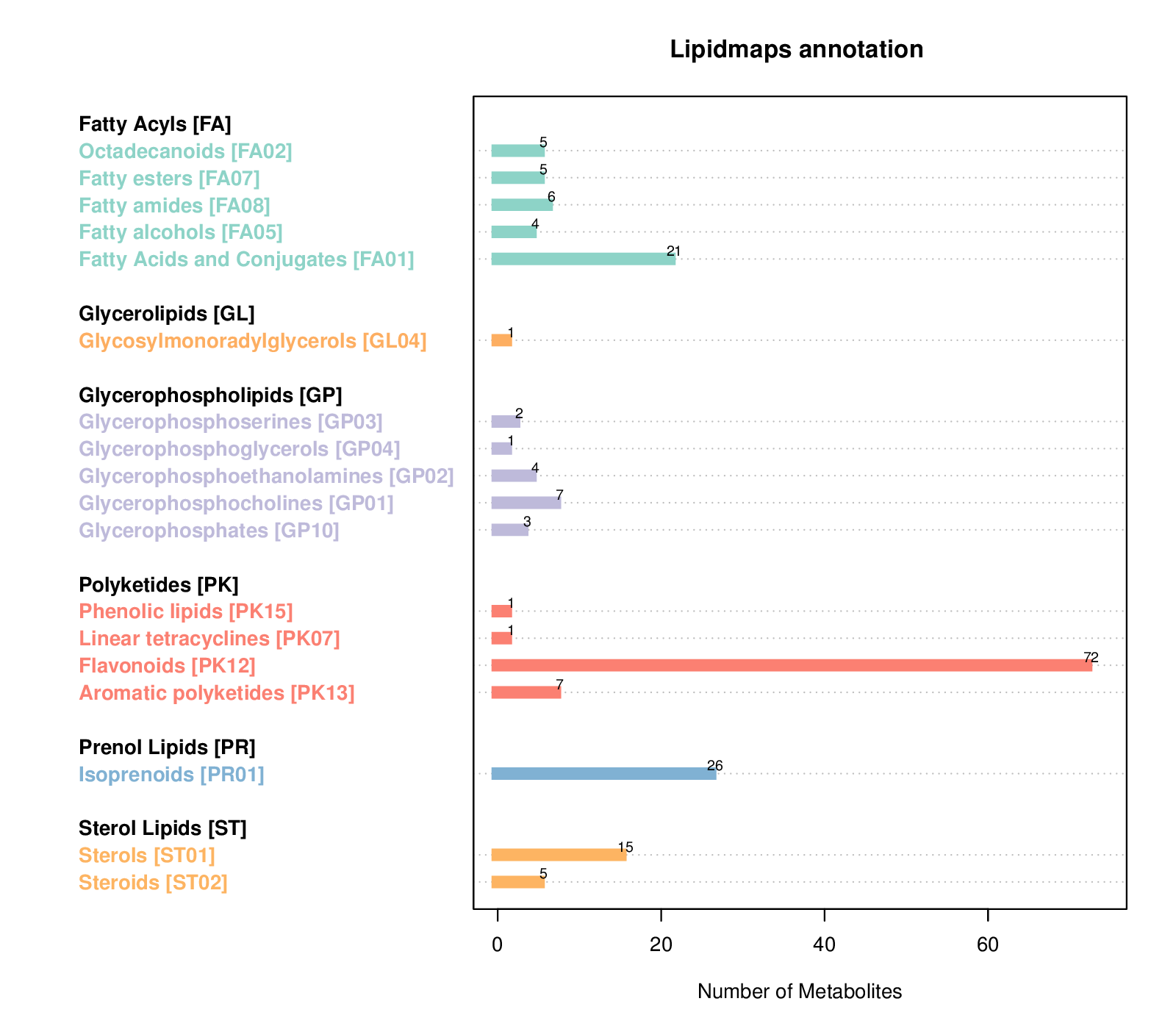

Supplement: Supplementary file 1 [file ijms-24-14761-s001.zip › Figure 7/meta_all.Lipidmaps.Anno.png]

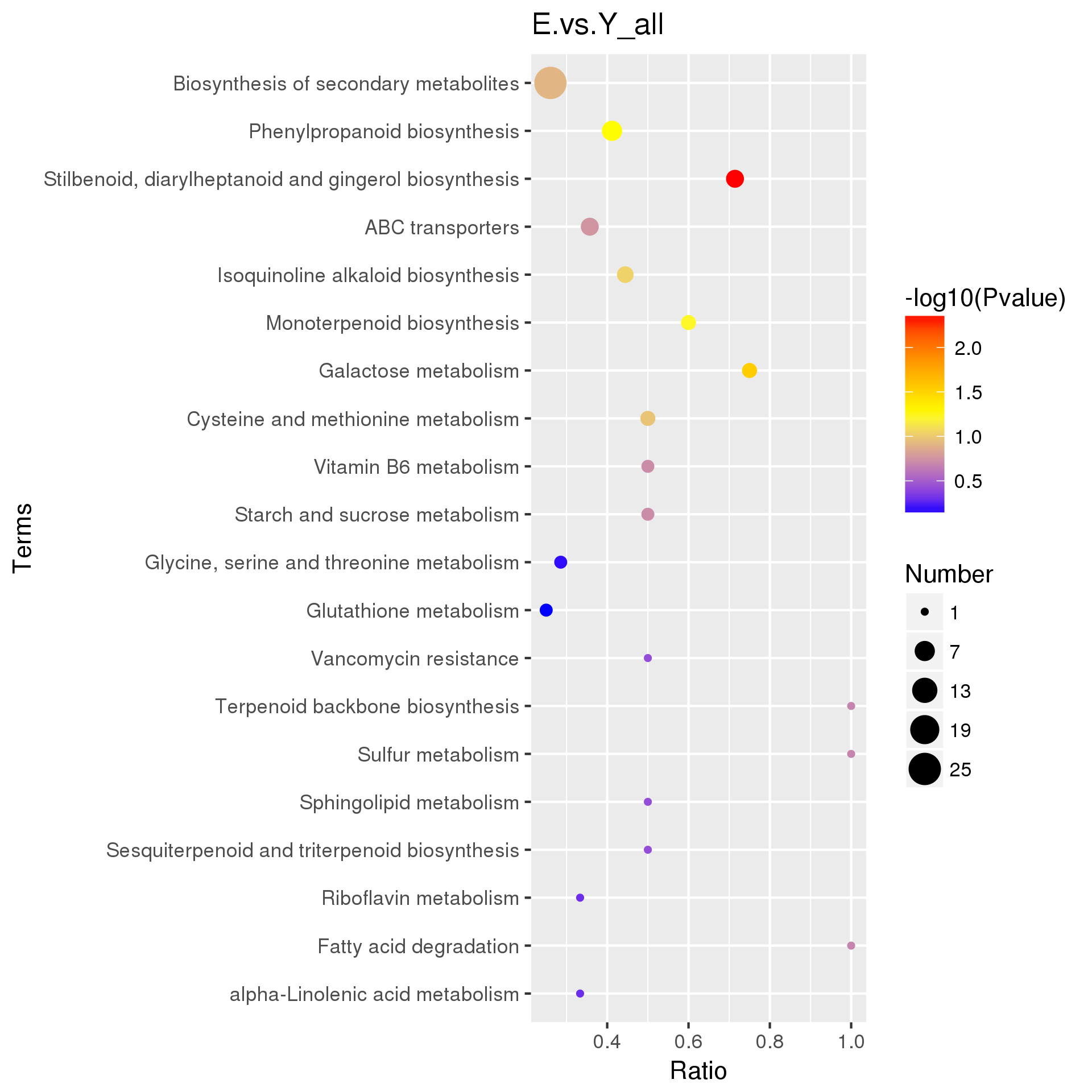

Supplement: Supplementary file 1 [file ijms-24-14761-s001.zip › Figure 8/E.vs.Y_all.KEGG_Enrich.scatterplot.png]

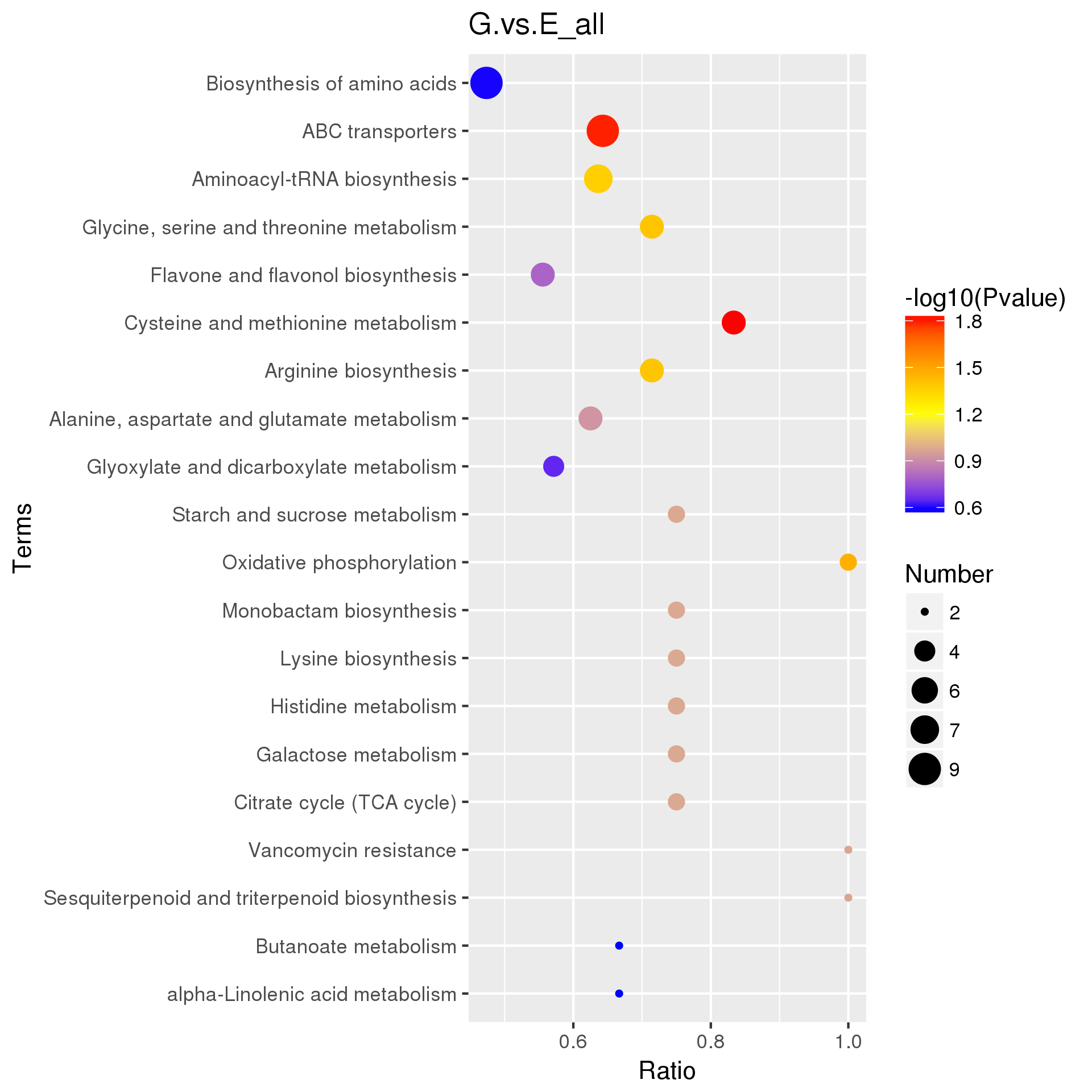

Supplement: Supplementary file 1 [file ijms-24-14761-s001.zip › Figure 8/G.vs.E_all.KEGG_Enrich.scatterplot.png]
